# Multiobjective differential evolution-based multifactor dimensionality reduction for detecting gene–gene interactions

**DOI:** 10.1038/s41598-017-12773-x

**Published:** 2017-10-09

**Authors:** Cheng-Hong Yang, Li-Yeh Chuang, Yu-Da Lin

**Affiliations:** 1 Department of Electronic Engineering, National Kaohsiung University of Applied Sciences, Kaohsiung, 80778 Taiwan; 2 Graduate Institute of Clinical Medicine, Kaohsiung Medical University, Kaohsiung, 80708 Taiwan; 3 Department of Chemical Engineering and Institute of Biotechnology and Chemical Engineering, I-Shou University, Kaohsiung, 84004 Taiwan

## Abstract

Epistasis within disease-related genes (gene–gene interactions) was determined through contingency table measures based on multifactor dimensionality reduction (MDR) using single-nucleotide polymorphisms (SNPs). Most MDR-based methods use the single contingency table measure to detect gene–gene interactions; however, some gene–gene interactions may require identification through multiple contingency table measures. In this study, a multiobjective differential evolution method (called MODEMDR) was proposed to merge the various contingency table measures based on MDR to detect significant gene–gene interactions. Two contingency table measures, namely the correct classification rate and normalized mutual information, were selected to design the fitness functions in MODEMDR. The characteristics of multiobjective optimization enable MODEMDR to use multiple measures to efficiently and synchronously detect significant gene–gene interactions within a reasonable time frame. Epistatic models with and without marginal effects under various parameter settings (heritability and minor allele frequencies) were used to assess existing methods by comparing the detection success rates of gene–gene interactions. The results of the simulation datasets show that MODEMDR is superior to existing methods. Moreover, a large dataset obtained from the Wellcome Trust Case Control Consortium was used to assess MODEMDR. MODEMDR exhibited efficiency in identifying significant gene–gene interactions in genome-wide association studies.

## Introduction

Single-nucleotide polymorphism (SNP) is a genetic variation of DNA sequences within a population. Genome-wide association studies (GWAS) covering a large quantity of SNPs provide an unbiased means of identifying disease-associated variants in genetic epidemiology^1–3^. Epistasis is the interaction effect between genes and could reveal the causes of complex diseases traits^4^. Investigating the gene–gene interactions of diseases and cancers could facilitate the understanding of epistasis in populations in the field of systems biology^5,6^. Statistical method, data mining, and machine learning have been used to detect epistasis in family-based and case-control studies, such as co-information based *n*-order eistasis detection and visualizer (CINOEDV)^7^, support vector machine-based method (EpiMiner)^8^, and so on^9^.

Multifactor-dimensionality reduction (MDR)^10^ and the predictive rule learning approach (SNPRuler)^11^ are proposed to facilitate epistatic investigation. MDR is a nonparametric data mining approach combining a contingency table measure [*k*-fold cross-validation (CV)] and a dimensionality reduction technique to detect gene–gene interactions in case–control studies^10,12^. SNPRuler is a nonparametric learning approach based on a predictive rule learning algorithm for identifying gene–gene interactions^11^. These methods have been applied to detect significant gene–gene interactions and investigate the effects of drugs^13^ on breast cancer^14^, oral cancer^15^, hypertension^16^, and other human diseases^5,17^.

Differential evolution (DE) is a powerful evolutionary algorithm^18^ that is popular for pattern recognition and optimization in engineering^19^. Multiobjective DE (MODE) is an improved DE modified to fit multiobjective problems^20^, in which *n* (*n* > 1) objectives are considered to synchronously search for optimal solutions^21^; for example, maximization objectives can be formulated as maximize(*f*_1_(*X*), …, *f*_*i*_(*X*)), where *X* ∈ $\widehat{X}$, *i* is the number of objectives, $\widehat{X}$ is the feasible solution set, and *f*(*X*) is an objective function. In maximization problems, solution *X*_1_ dominates solution *X*_2_ if *f*_*j*_(*X*_1_) > *f*_*j*_(*X*_2_) for all indices *j* ∈ (1, …, *n*). Pareto optimal solution sets (Pareto sets) represent a powerful technique for collecting good solutions not dominated by one another. These good solutions are the results of MODE.

Several contingency table measures, such as chi-square, likelihood ratio, normalized mutual information (NMI), and *et al*., have been applied to score model quality in MDR^22,23^, and these measures can be regarded as various objectives in MODE. Currently, MDR-based methods focus only on a single measure to determine gene–gene interactions. Various simulation dataset types have been adopted to evaluate which contingency table measures can significantly improve MDR performance^22^, revealing that MDR performance could be measured based on the correct classification rate (CCR)^10^ or NMI^22^. However, no optimal measure for determining gene–gene interactions involving various dataset types has yet been found. Each measure may fit specific dataset types; however, deriving data distributions from real datasets is difficult, especially for complex diseases. Therefore, developing a method that can synchronously consider multiple measures to detect gene–gene interactions is essential.

In this study, a multiobjective DE (hereafter MODEMDR) was proposed to merge various contingency table measures based on MDR and detect significant gene–gene interactions. Two objectives involving the aforementioned two measures of CCR and NMI were selected for MODEMDR. Several epistatic models with and without marginal effects and with various parameter settings (heritability (*h* ^2^) and minor allele frequencies (*MAF*)) were selected to generate high-dimensional simulation datasets. In addition, a large real dataset was obtained from the Wellcome Trust Case Control Consortium (WTCCC)^24^. The results of the simulation and real datasets indicated that MODEMDR can effectively detect gene–gene interactions.

## Results

### Simulation data experiments

The goal of the simulation datasets was to successfully detect the specific two-locus SNP combination (target) in each artifact epistasis model. Epistatic models with and without marginal effects were simulated to compare the epistatic interaction identification ability of SNPRuler^11^, MDR^25^, single measure DE MDR (DEMDR), and MODEMDR.

### Comparison between MODEMDR and existing methods on disease loci with marginal effects

The eight epistatic models with marginal effects were used to evaluate the performance of SNPRuler, MDR, DEMDR (CCR), and MODEMDR. Models 1–6 were obtained from Namkung *et al*.^23^ and models 7 and 8 were obtained from Bush *et al*.^22^. These models reflect the strength of genetic effects and were proposed according to the interaction structure, *MAF*, and prevalence. The details of the multilocus penetrances of the eight models are shown in Table 1 in the supplementary file. The penetrances of the eight models were computed under the Hardy–Weinberg equilibrium (HWE) assumption for each SNP. In each model, 100 datasets were simulated under identical settings with uniform *MAF* of [0.05, 0.5). The detection success rate was computed as the proportion of the generated datasets, in which a target of epistatic interaction was detected. GAMETES software was used to simulate the simulation datasets^26^.

**Figure 1:**
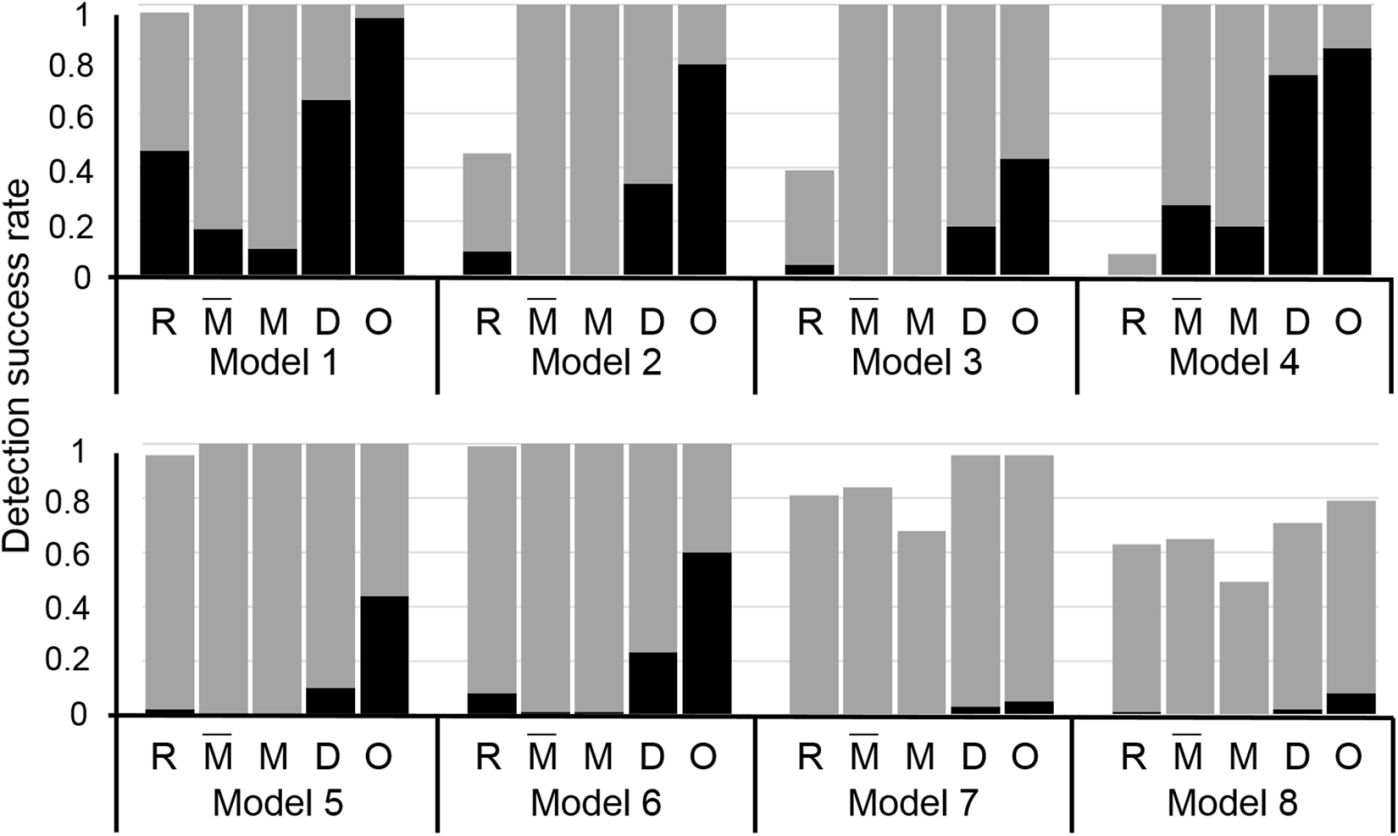
Comparison between SNPRuler (R), MDR(CVC ≥ 3) $(\bar{{\rm{M}}})$, MDR(CVC ≥ 4) (M), DEMDR (D), and MODEMDR (O) across eight pure epistatic models with marginal effects. For each model, the detection success rate was calculated as the proportion of 100 datasets in which the specific disease-associated epistatic interaction was detected. Each dataset contained 1,000 SNPs. The gray bars represent the detection success rate for 1,000 cases and 1,000 controls. The black bars represent the detection success rate for 200 cases and 200 controls. No bars indicates a detection success rate of zero.

**Figure 2:**
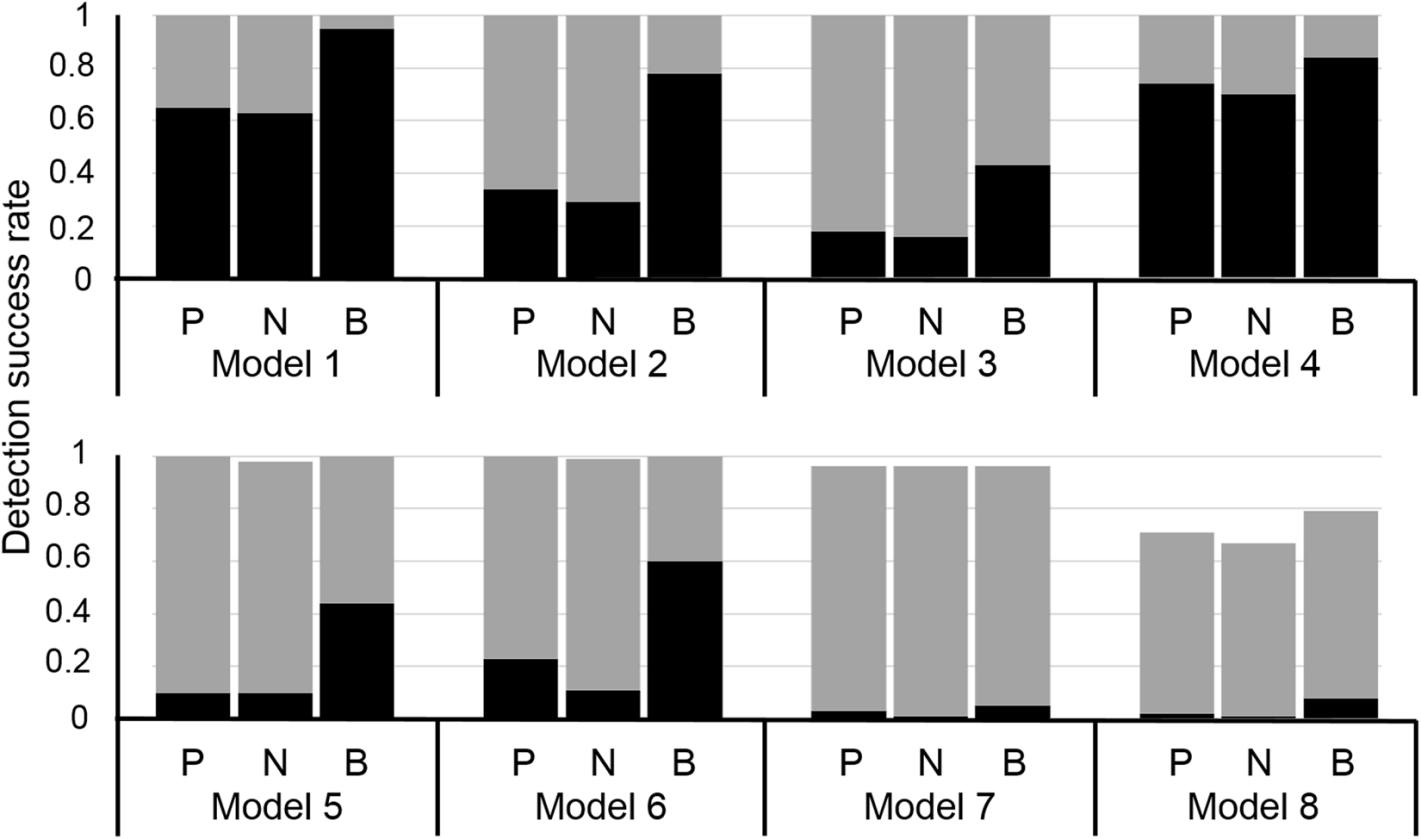
Comparison between the CCR (P), NMI (N), and both measures (B) across eight pure epistatic models with marginal effects. Under each setting, the detection success rate was calculated as the proportion of 100 datasets in which a specific disease-associated epistatic interaction was detected. Each dataset contained 1,000 SNPs. The gray bars represent the detection success rate for 1,000 cases and 1,000 controls. The black bars represent the detection success rate for 200 cases and 200 controls. The absence of bars indicates a detection success rate of zero.

In the eight models, MDR, DEMDR, and MODEMDR outperformed SNPRuler in the large samples (Fig. 1; 1,000 cases and 1,000 controls), in which MODEMDR outperformed MDR and DEMDR in models 7 and 8. Regarding the small samples (200 cases and 200 controls), SNPRuler, MDR, and DEMDR had difficulties identifying the specific two-locus SNP combinations in the epistatic models with marginal effects. Clearly in the small samples, MODEMDR outperformed MDR, DEMDR, and SNPRuler in the eight epistatic models with marginal effects. The generated datasets of eight epistatic models with marginal effects were used to compare DEMDR (CCR) (P), DEMDR (NMI) (N), and MODEMDR (two objectives merging CCR and NMI) (B). DEMDR (CCR) achieved higher detection success rates than DEMDR (NMI) in all epistatic models with marginal effects (Fig. 2). Moreover, MODEMDR outperformed DEMDR (CCR) and DEMDR (NMI), indicating that multiple contingency table measures are superior to single contingency table measures in detecting epistatic interactions with marginal effects. MODE effectively improves MDR with respect to performing evaluations to facilitate the identification of significant gene–gene interactions.

### Comparison between MODEMDR and existing methods on disease loci without marginal effects

A total of 60 two-locus epistatic models were obtained from Wan *et al*.^11^ and used to assess the performance of SNPRuler, MDR, DEMDR (CCR), and MODEMDR. These models are pure epistatic models (i.e., they have no marginal effects). The multilocus penetrances are shown in Supplementary Table 2. The parameter settings (*h* ^2^ and *MAF*) were selected to generate simulation data by using GAMETES software^26^. The *h* ^2^ controlled the phenotypic variation of the 60 models and ranged from 0.025 to 0.4. The *MAF* ranged from 0.2 to 0.4. For each epistatic model, the 100 datasets consisting of 1,000 SNPs, 200 cases, and 200 controls were generated. The detection success rate was calculated as the proportion of the 100 datasets in which the specific disease-associated two-locus SNP combination was detected.

**Figure 3:**
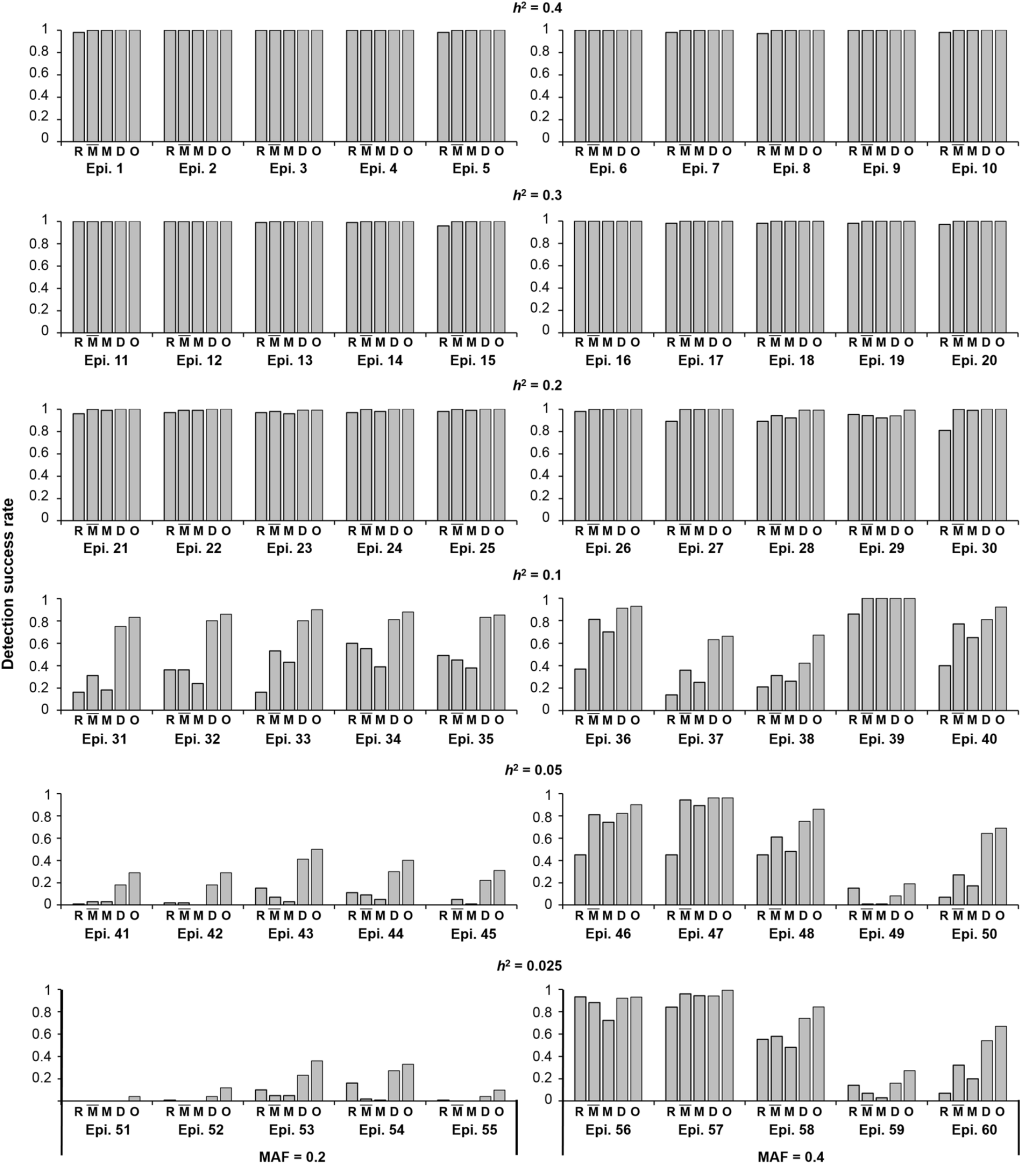
Comparison between SNPRuler (R), MDR(CVC ≥ 3) $(\bar{{\rm{M}}})$, MDR(CVC ≥ 4) (M), DEMDR (D), and MODEMDR(O) across 60 pure epistasis models without marginal effects. Under each setting, the detection success rate was calculated as the proportion of 100 datasets in which the specific disease-associated epistatic interaction was detected. Each dataset contained 1,000 SNPs. The gray bars represent the detection success rate for 200 cases and 200 controls. No bars indicates a detection success rate of zero.

**Figure 4:**
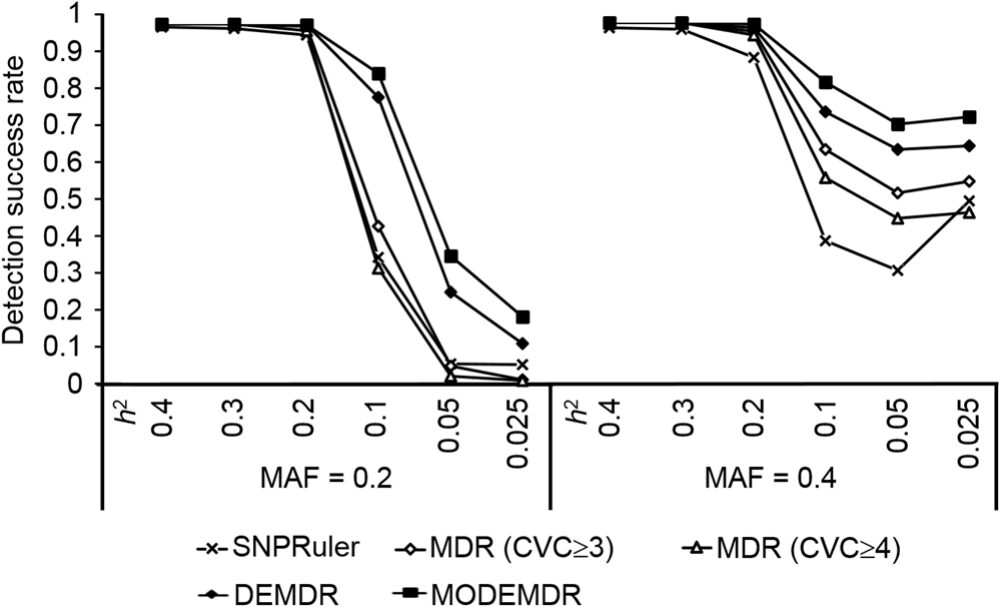
Comparison of the impact of MAF and h2 on the detection success rates of SNPRuler, MDR(CVC ≥ 3), MDR(CVC ≥ 4), DEMDR, and MODEMDR across 60 pure epistasis models. Under each setting, the detection success rate was calculated from 100 datasets containing 1,000 SNPs genotyped from 200 cases and 200 controls.

In the 60 models, MODEMDR outperformed SNPRuler, MDR, and DEMDR in detecting epistatic interactions without marginal effects (Fig. 3). The results of Wilcoxon signed-rank testing (Table 1) showed that MODEMDR achieved the highest R^+^ (number of victories), lowest R^−^ (number of losses), and a *p* value of < 0.05, indicating that MODEMDR is significantly superior to the other methods. In the epistatic models with *MAF* = 0.2 or 0.4 and *h* ^2^ ≥ 0.2, all detection success rates of SNPRuler, MDR, DEMDR, and MODEMDR were ≥ 80%, which degraded as *h* ^2^ was decreased. When *MAF* = 0.2 and *h* ^2^ ≤ 0.05, DEMDR and MODEMDR achieved detection success rates of approximately 30% and 40%, respectively. By contrast, SNPRuler and MDR almost completely lost their detection abilities. MODEMDR achieved the highest detection success rates for all settings, especially *h* ^2^ ≤ 0.01 (Fig. 4). All the test results show that MODEMDR outperformed SNPRuler, MDR, and DEMDR in the epistatic models with no marginal effects.

**Table 1:** Comparison of SNPRuler, MDR, DEMDR, and MODEMDR across 60 epistasis models using Wilcoxon signed-rank testing.

		N	Mean Rank	Sum of Ranks	Z-test	*P* value
MODEMDR *vs*. SNPRuler	R^−^	3	9.67	29	−5.952	2.65E-09
	R^+^	48	27.02	1297		
	R^=^	9				
	Total	60				
MODEMDR *vs*. MDR(CVC ≥ 3)	R^−^	1	4	4	−4.784	1.72E-06
	R^+^	30	16.4	492		
	R^=^	29				
	Total	60				
MODEMDR *vs*. MDR(CVC ≥ 4)	R^−^	0	0	0	−5.161	2.46E-07
	R^+^	35	18	630		
	R^=^	25				
	Total	60				
MODEMDR *vs*. DEMDR	R^−^	0	0	0	−4.71	2.47E-06
	R^+^	29	15	435		
	R^=^	31				
	Total	60				

R^−^: number of epistasis models when MODEMDR lost another algorithm; R^+^: number of epistasis models when MODEMDR won another algorithm; R^=^: number of epistasis models when two algorithms tied; N: number of R^−^, R^+^, and R^=^. *P* < 0.05 indicates a significant difference between two algorithms.

**Figure 5:**
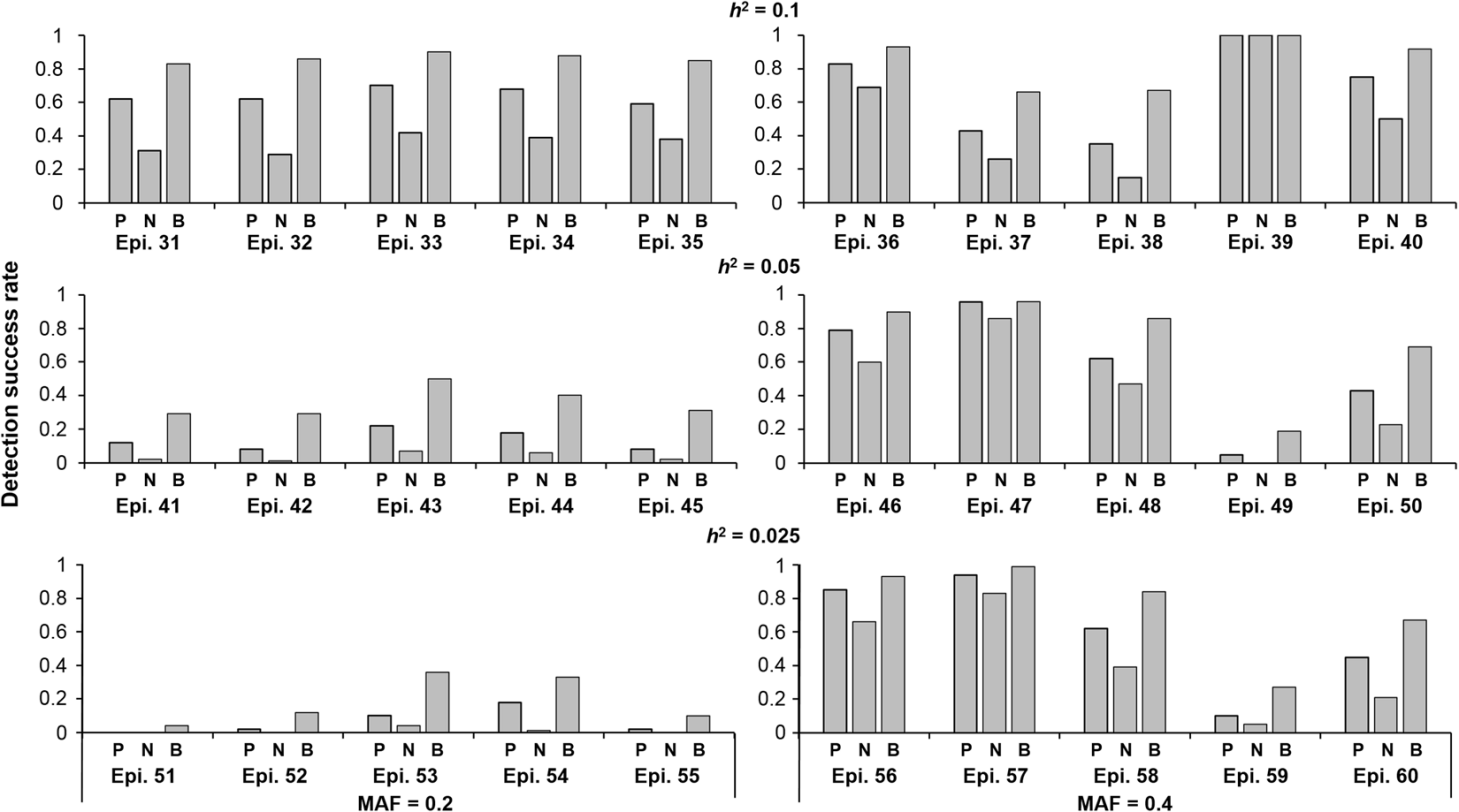
Comparison between the CCR measure (P), NMI measure (N), and both measures (B) across epistasis models 31–60 without marginal effects. Under each setting, the detection success rate was calculated as the proportion of 100 datasets in which the specific disease-associated epistatic interaction was detected. Each dataset contained 1,000 SNPs. The gray bars represent the detection success rate for 200 cases and 200 controls. No bars indicates a detection success rate of zero.

The generated datasets of models 31–60 were used to compare the DEMDR (CCR) (D), DEMDR (NMI) (N), and MODEMDR (two objectives merging CCR and NMI) (O) in detecting epistatic interactions without marginal effects. Detection success rates were calculated as the proportion of the 100 datasets in which the specific disease-associated two-locus SNP combination was identified. DEMDR (CCR) achieved a higher detection success rate than DEMDR (NMI) in all epistatic models without marginal effects (Fig. 5). However, MODEMDR outperformed DEMDR (CCR) and DEMDR (NMI), indicating that multiple contingency table measures are superior to single contingency table measures for identifying gene–gene interactions.

### Results of WTCCC data

To evaluate the ability of MODEMDR to handle large datasets, a large dataset was obtained from the WTCCC^24^, consisting of 500,569 SNPs, including 1,988 cases of coronary artery disease (CAD) and 1,500 controls obtained from people living in Great Britain who self-identified as white Europeans.

The epistatic interactions detected by MODEMDR are shown in Table 2. The gene names in Table 2 were obtained from dbSNP at the National Center for Biotechnology Information. The designation “UNKNOWN” in the table refers to SNP not being located on a gene. The minimum and maximum numbers of detected significant epistatic interactions in each chromosome were 1 and 6, respectively. The *p* value was estimated through an χ^2^ test using the raw datasets to determine the significance level for epistatic interaction between two SNPs^11^. All epistatic interactions detected by MODEMDR in 24 chromosomes yielded a *p* value of <0.0001, indicating a strong significant interaction between two SNPs. When the CCR was larger than 0.5, the frequency of chance can be significantly reduced^27^, indicating that our results identified significant epistatic interactions. The high NMI values indicate that uncertainty was reduced in the model in a true state. The CCR and NMI values show the multiobjective optimization property. The epistatic interaction with the highest NMI value (rs41399650, rs41397248) was not the epistatic interaction with the highest CCR value (rs41399650, rs17163057) in chromosome 1. Further detection of significant epistatic interactions may provide an etiological understanding of epistasis in systems biology^28^. The MODEMDR for CCR measures was located between 0.588 and 0.959 and the mean CCR was 0.750 (standard deviation (SD) = 0.096). The NMI measures were located between 0.033 and 0.759 and the mean NMI was 0.267 (SD = 0.182). Notably, the epistatic interaction of SNPs rs16926425 and rs7299571 (chromosome 12) obtained the highest CCR (0.959) and NMI values (0.759). The ten detected epistatic interactions indicate the beneficial measures of NMI > 0.4 and CCR > 0.8 (marked by stars in Table 2). The details of all epistatic interactions are shown in Supplementary Fig. 1. In all figures, the (black) left bar in a class represents the frequency of cases and the (white) right bar represents the frequency of controls. Gray classes indicate being in the high-risk group.

**Table 2:** Summary of MODEMDR results for CAD based on WTCCC data.

Location^a^	SNP Groups	Related Genes	CCR	NMI	Times^b^ (s)
Chr1	rs41399650, rs17163057	UNKNOWN, UNKNOWN	0.798	0.319	63.6
	rs41399650, rs853747	UNKNOWN, PROX1-AS1	0.768	0.351	
	rs41399650, rs41397248	UNKNOWN, UNKNOWN	0.767	0.353	
Chr2	rs41509345, rs41453947	NCKAP5, UNKNOWN	0.798	0.282	63.1
Chr3	rs41367044, rs10866051*	GTF2E1, UNKNOWN	0.846	0.438	61.5
	rs4552351, rs10866051*	UNKNOWN, UNKNOWN	0.826	0.451	
	rs16828468, rs10866051*	UNKNOWN, UNKNOWN	0.824	0.451	
Chr4	rs41426946, rs41529544	PPA2, UNKNOWN	0.810	0.313	62.2
Chr5	rs41505353, rs41421845	SPOCK1, UNKNOWN	0.705	0.137	62.2
Chr6	rs41509944, rs41489047	UNKNOWN, BAI3	0.784	0.248	62.0
	rs41421547, rs41489047	UNKNOWN, BAI3	0.773	0.254	
	rs16885600, rs41489047	CASC15, BAI3	0.777	0.252	
Chr7	rs41437948, rs41468749	POU6F2, WBSCR17	0.683	0.102	62.7
	rs7777155, rs41437948	ZNF92, POU6F2	0.683	0.106	
Chr8	rs35120859, rs17480050	UNKNOWN, CSGALNACT1	0.754	0.197	61.1
	rs17480050, rs16883114	CSGALNACT1, LINC01288	0.683	0.202	
Chr9	rs41354745, rs41424148	KANK1, UNKNOWN	0.727	0.199	61.7
Chr10	rs41370151, rs2944490	FAM107B, TCERG1L	0.791	0.338	62.0
Chr11	rs41518446, rs41381045	MAML2, SHANK2	0.672	0.088	61.9
Chr12	rs16926425, rs7299571**	SOX5, UNKNOWN	0.959	0.759	62.2
Chr13	rs7328649, rs9540734	FAM155A, PCDH9	0.825	0.338	61.9
Chr14	rs41324950, rs1884094	UNKNOWN, UNKNOWN	0.807	0.292	60.9
	rs41491051, rs41324950	SLC35F4, UNKNOWN	0.804	0.300	
Chr15	rs41418548, rs41418744	SHC4, UNKNOWN	0.701	0.123	61.3
	rs41418548, rs41467146	SHC4, UBE2Q2P1	0.701	0.123	
Chr16	rs235633, rs41483646	UNKNOWN, UNKNOWN	0.768	0.226	61.2
Chr17	rs3785579, rs12939469*	CACNG1, UNKNOWN	0.878	0.552	61.7
	rs3785579, rs4795043*	CACNG1, UNKNOWN	0.878	0.555	
	rs3785579, rs180171*	CACNG1, UNKNOWN	0.877	0.560	
	rs3785579, rs1870998*	CACNG1, UNKNOWN	0.877	0.559	
	rs3785579, rs3902104*	CACNG1, BCAS3	0.877	0.56	
Chr18	rs41470446, rs3794931	UNKNOWN, ZNF516	0.756	0.200	61.4
	rs3794931, rs4799934	ZNF516, CELF4	0.746	0.303	
Chr19	rs375299, rs41370444	UNKNOWN, UNKNOWN	0.641	0.062	61.4
	rs375299, rs11671119	UNKNOWN, MEF2BNB-MEF2B	0.579	0.074	
Chr20	rs2748666, rs41405046*	UNKNOWN, UNKNOWN	0.884	0.488	61.7
	rs16988533, rs2748666*	UNKNOWN, UNKNOWN	0.845	0.493	
Chr21	rs2837906, rs41451052	UNKNOWN, UNKNOWN	0.600	0.033	61.3
	rs429380, rs41451052	DSCAM, UNKNOWN	0.588	0.041	
Chr22	rs10212068, rs41416344	HMGXB4, CHCHD10	0.648	0.068	60.2
	rs10212068, rs41431147	HMGXB4, TXNRD2	0.616	0.080	
	rs10212068, rs5748617	HMGXB4, UNKNOWN	0.625	0.078	
	rs10212068, rs1054055	HMGXB4, CHCHD10	0.646	0.071	
	rs10212068, rs41459445	HMGXB4, HMGXB4	0.645	0.075	
	rs10212068, rs16992075	HMGXB4, UNKNOWN	0.596	0.080	
ChrX	rs1419930, rs41500547	UNKNOWN, DMD	0.665	0.095	67.9

^a^Chr chromosome; ^b^MODEMDR running time; time unit: hour (h); **optimal epistatic interaction; *top ten epistatic interactions.

The running times of chromosomes in the WTCCC dataset are shown in Table 2. MDR required approximately 18.3 h to analyze the chromosome with the largest data (chromosome 2), whereas MODEMDR required only approximately 63 s. Regarding the average running times for all large datasets, MDR required approximately 6.15 h, whereas MODEMDR required only approximately 62.05 s, indicating that MODEMDR has the shortest running time for analyzing large datasets.

## Discussion

MODE enables MDR to use multiple measures to detect gene–gene interactions. Although the CCR in MDR-based methods is a powerful measure for determining such interactions, it could fail to determine interactions in some epistatic models (e.g., models 31–60 without marginal effects (Fig. 5)). Furthermore, the NMI could not always determine specific targets within models 31–60 without marginal effects. Therefore, both measures were considered for synchronous use to effectively determine the targets (Table 2). MODEMDR can effectively detect gene–gene interactions because the MODE fits the joint effect property^29^, which consists of the main effect, overall effect, and high-order interaction effect. The main effect refers to any effect that could serve as a guide to identifying the correct multilocus interaction. The overall effect refers to an effect that commonly appears among *n* risk factors. The high-order interaction effect refers to the least proper subset of the loci that interacts epistatically. SNPs strongly associated with diseases or cancers are often likely to be significant factors in high-locus interactions. High CCR and NMI values in MODEMDR indicate a more significant risk of *n*-factor effects. In the MODEMDR selection operation, promising SNPs can be retained for the next generation. These SNPs are subsequently combined through mutation and recombination operations to produce better SNP combinations, enabling MODEMDR to detect the significant epistatic interactions.

In MDR, combinations of high-dimensional factors can be reduced by assigning multilocus genotypes to high- or low-risk groups, enabling gene–gene interaction quality to be measured through two-way contingency table analysis^10^. CCR is the measure most commonly applied in MDR-based methods^30^. Bush *et al*. (2008) compared the ten general measures in the text classification field to evaluate the degree of improvement in the ability of MDR to detect gene–gene interactions. CCR and NMI were suggested as being able to improve MDR identification in the simulation^22^. The results of the present study exhibited the most successful gene–gene interaction identification when the NMI and CCR were used to synchronously determine significant gene–gene interactions.

The WTCCC dataset is well-known in GWAS analyses, in which large SNPs in chromosomes are collected. MODEMDR efficiently identifies gene–gene interactions from combinatorially explosive search spaces (running time in Table 2), and uses the rational performing time (population size × generation size) to calculate MDR measures, enables it to handle GWAS analysis. MODEMDR has the advantages of MDR because the fitness functions of multiple objectives are designed based on MDR. The advantages of MODEMDR include the following: (i) suitability for application in small sample datasets, (ii) suitability for application in unbalanced datasets, (iii) ability to describe the loci genotype combinations associated with high and low risk of disease, (iv) the model-free method, (v) ability to detect a higher-order gene–gene interactions, and (vi) the nonparametric method.

MODEMDR was applied in this study for synchronous consideration of the multiple measures used to detect significant gene–gene interactions. To our knowledge, MODEMDR is the first MDR-based method that accounts for multiple measures. The experimental results demonstrate that multiple measures engender an identification performance superior to that of the MDR-based single measure method. In the WTCCC analysis, MODEMDR successfully handled the large-scale dataset in terms of speed and identification of significant gene–gene interactions. Furthermore, MODEMDR provides a multiobjective method for identifying gene–gene interactions. Improvements could be made by using more combinations among various measures in a two-way contingency table.

## Methods

### Definitions of multiobjective optimization in gene–gene interaction identification

Consider a multiobjective maximization problem with *m* parameters (decision variables) and *n* objectives without the loss of generality: Maximize $\vec{f}(X)=[{f}_{1}({x}_{1},\,\mathrm{...},{x}_{m}),\mathrm{...},{f}_{n}({x}_{1},\,\mathrm{...},{x}_{m})]$ where $X=({x}_{1},\mathrm{...},{x}_{m})$ and $\vec{f}=({f}_{1},\mathrm{...},{f}_{n})$ where *X* is the decision vector and $\vec{f}$ is the objective vector. For *X*_*i*_, all objectives $\vec{f}$ that are not dominated by any other vector *X*_*j*_ (*j* = 1, …, *k* | *i*≠*j*) where *k* is the population size are called nondominated points. For gene–gene interaction identification, we defined “gene–gene interaction” (i.e., solution) as a decision vector and “measures” as the corresponding objective vector. Here, CCR^10^ and NMI^22^ were defined as *f*_1_ and *f*_2_, respectively. Therefore, in this study, the objective was defined as follows:

1 $${\rm{Maximize}}\{\begin{array}{c}{f}_{1}({X}_{i})=CCR({X}_{i})\\ {f}_{2}({X}_{i})=NMI({X}_{i})\end{array}$$

where *X* is the solution space and *X*_*i*_ ∈ *X*.

### MODEMDR

In MODEMDR, the MDR operation process is modified to apply MDR as a fitness function in MODE. In addition, a balance strategy is introduced in data preprocessing within cross-validation in MDR to improve the accuracy of fitness evaluation. The balance strategy can effectively increase the CCR in the training and testing. In MODE operations, target vector *X*, mutant vector *V*, and trial vector *U* are used to seek the optimal multiobjective set. A target vector is a feasible solution for identifying gene–gene interactions. Pareto operations generate extra storage and use Pareto set filter operators to save all nondominated individuals in each generation. During initialization, the target vectors are randomly generated in the feasible problem space. A Pareto set is initialized in an empty space because the individuals have not been evaluated. The first operation is mutation operation, which generates the mutant vectors of individuals based on the sum of the weighted difference between two vectors and a third vector, which are randomly selected from the population or Pareto set. Subsequently, recombination operation generates the trial vectors of individuals by mixing the mutant vectors with the parameters of other predetermined target vectors. Boundary constraint operation is used to verify that the trial vectors are feasible solutions. If a trial vector is not a feasible solution, its parameters are adjusted to render it feasible. In selection operation, the target vector is updated if the trial vector yields to dominate the target vector. Finally, the Pareto set is updated if the target vectors dominate the individuals in the set. Thus, the Pareto optimal solution set, called the “Pareto front,” can be improved throughout the generation. The MODEMDR process is shown in Algorithm 1, the steps of which include data preprocessing, Pareto operation, and the following four basic DE operations: mutation, recombination, boundary constraint, and selection.
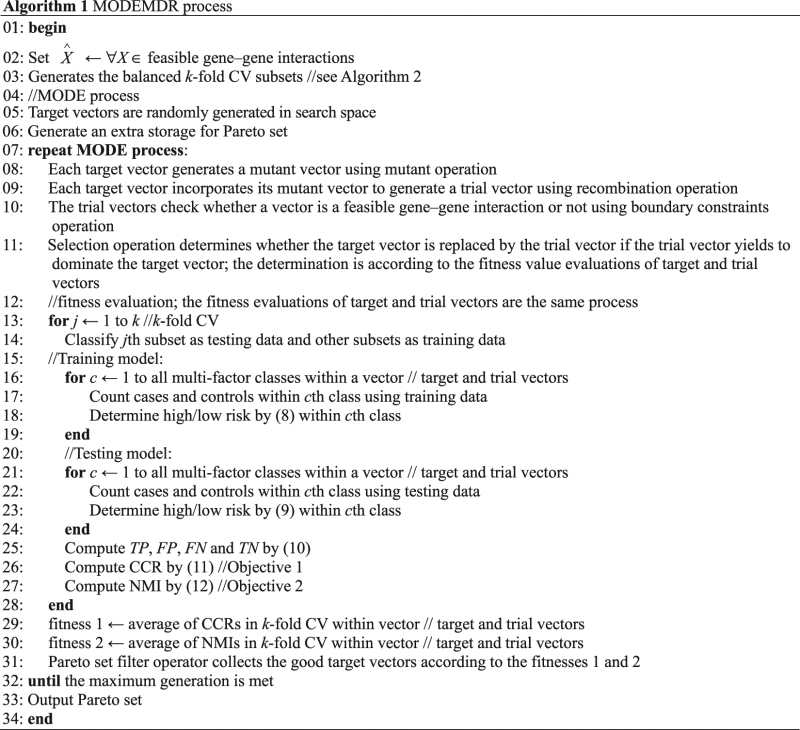
 
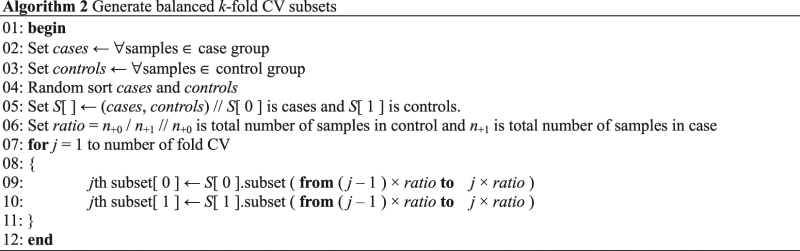


### Data preprocessing

Data preprocessing uses the balance strategy in MDR to handle the balanced *k*-fold CV subsets that are divided by the original dataset for objective evaluation. For *k*-fold CV operation, the balanced *k*-fold CV subsets are generated through the following five steps of the balance strategy:

1. Step 1. Divide the samples into case sets (cases) and control sets (controls).
2. Step 2. Randomly shuffle the case and control samples.
3. Step 3. Count the total numbers of cases and controls.
4. Step 4. Compute the ratio between cases and controls.
5. Step 5. Assign the case and control samples to subset *j* (*j* = 1, …, *k*) according to the ratio, where *j* is the CV index and *k* is the total number of CV subsets.

### Pareto operation

The Pareto operation uses a Pareto set filter operator to collect good individuals (target vectors) according to the multiobjective values, where the individuals do not dominate one another. These individuals are saved in extra storage *S* = (*s*_1_, …, *s*_*i*_), where *s* is the target vector and *i* is the registration size, which is the maximum number of individuals in storage. The Pareto set filter operator consists of the following two steps:

1. Step 1. Comparison between an *X* ∈ population and *s*_*j*_ for all indices *j* ∈ (1, …, *i*) in *S*. If *X* is not dominated by any *s*_*j*_, *X* is added into *S*.
2. Step 2. Comparison between an s_*j*_ for index *j* ∈ (1, …, *i*) and s_*k*_ for indices *k* and *k* ∈ (1, …, *i | k* *≠* *j*) in *S*. If s_*j*_ is dominated by any s_*k*_, s_*j*_ is discarded.

### Target vector definition

Let *X*_*i*,*g*_ = (*x*_1,*i*,*g*_, .., *x*_*d*,*i*,*g*_) be the *i*th target vector in the population for the *g*th generation in the *d*-dimensional search space. A target vector is a gene–gene interaction in which the parameters are the SNP indices and are all different in a target vector. Given that *y*-SNPs and *d*-order gene–gene interaction identification are considered in case–control studies, the target vector *X*_*i*_ is represented as follows:

$${X}_{i,g}=({x}_{1,i,g},\ldots ,{x}_{d,i,g}|x\in (1,\ldots ,y)),$$

where *g* is the *g*th generation.

For initialization (i.e., *g* = 0), the parameter *x*_*j*_ (*j* = 1, …, *d*) in the target vector *X*_*i*_ is randomly generated by (2):

2 $${x}_{j,i,0}=ran{d}_{j,i}\times (upper-lower)+lower$$

where *upper* and *lower* represent the upper and lower boundaries of the indices of independent variables, respectively. The rand_*j*,*i*_ is the random number generator, which returns a uniformly distributed random value from within the range [0, 1).

### Mutation

Each target vector generates a mutant vector *V*_*i*,*g*+1_, which is a vector sum of the weighted difference between two vectors and a third vector, expressed as follows:

3 $${V}_{i,g+1}={X}_{r1,g}+F\cdot ({X}_{r2,g}-{X}_{r3,g}),i=1,\ldots ,n$$

and

4 $${X}_{ri,g}=\{\begin{array}{cc}{S}_{ri,g} & {\rm{if}}\,randb\le PV,\\ {X}_{ri,g} & {\rm{if}}(randb(j) > PV)\,{\rm{or}}S={\rm{empty}},\end{array}\quad \quad i=1,\,2,\,3$$

In (3), *n* is the population size; *r*_1_, *r*_2_, and *r*_3_ ∈ (1, …, *n*) are the random indices of the storage (Pareto operation) or population; *g* is the *g*th generation; *X*_*r*1,*g*_, *X*_*r*2,*g*_, and *X*_*r*3,*g*_ are the selected three target vectors from the storage or population, where all selected target vectors are different; and *F* is a real and constant factor ∈ [0, 2) that controls the amplification of the differential variation (*X*_*r*2,*g*_ − *X*_*r*3,*g*_). In (4), S_*ri*,g_ is the *r*_*i*_th target vector [*r*_*i*_ ∈ (1, …, *n*)] in the storage and *PV* is a mutation constant ∈ [0, 1) that controls the probability of the vector selected from either the storage or population.

### Recombination

Recombination operation can increase the vector diversity in the population. The trial vector *U*_*i*,*g*+1_ is expressed as (5) and the parameters of the trial vector are computed by (6), which incorporates the mutant vector *V*_*i*,*g*+1_ and current target vector *X*_*i*,*g*_ at the *i*th target vector, expressed as follows:

5 $${U}_{i,g+1}=({u}_{1,i,g+1},\ldots ,{u}_{d,i,g+1})$$

and

6 $${u}_{j,i,g+1}=\{\begin{array}{c}{v}_{j,i,g+1}\,{\rm{if}}\,(randb(j)\le CR)\,{\rm{or}}\,j=rnbr(i),\\ {x}_{j,i,g}\,{\rm{if}}\,(randb(j) > CR)\,{\rm{or}}\,j\ne rnbr(i),\,\,\,\,\,\,\,\,\,\,\,\,j=1,2,\ldots ,d\end{array}$$

In (5), *i* is the trial vector index in the population, *d* is the dimension size, and *g* is the *g*th generation. In (6), *j* is the index of the dimension in the mutant vector *V*_*i*,*g*_ and target vector *X*_*i*,*g*_, where the two *i*s represent the indices of the mutant vector and target vector in the population; *randb*(*j*) is the *j*th evaluation of a uniform random number generator with the outcome ∈ [0, 1); *CR* is the crossover constant ∈ [0, 1); and *rnbr*(*i*) is a randomly chosen index ∈ (1,…, *d*) that ensures that *U*_*i*,*g*+1_ obtains at least one parameter from *V*_*i*,*g*+1_.

### Boundary constraints

Boundary constraints can ensure that trial vectors are feasible combinations. Equation (7) guarantees that trial vector parameters do not violate boundary constraints with random values generated by (2), expressed as follows:

7 $${u}_{j,i,g+1}=\{\begin{array}{ll}ran{d}_{j}\times (upper-lower)+lower, & {\rm{if}}\,({u}_{j,i,g} < lower\,{\rm{or}}\,{u}_{j,i,g} > upper)\,{\rm{or}}\,(\exists {!}{u}_{j,i,g}\in {U}_{i,g})\\ {u}_{j,i,g}, & {\rm{otherwise}}\end{array}$$

where *j* is an index of the dimension in the trial vector *U*_*i*,*g*_, *i* is the index of the trial vector in the population, *g* is the *g*th generation, *upper* and *lower* are the upper and lower bounds of the indices of independent variables, respectively, and ∃!*u*_*j*,*i*,*g*_ represents a variable at the *j*th parameter only existing in the *i*th trial vector for the *g*th generation.

### Selection

Selection operation determines whether the target vector *X*_*i*,*g*_ is dominated by the trial vector *U*_*i*,*g*_; in other words, *f*_*j*_(*U*_*i*,*g*_) > *f*_*j*_(*X*_*i*,*g*_) for all indices *j* ∈ (1, 2), where *j* is the index of the objective function. If the trial vector *U*_*i*,*g*+1_ dominates the target vector *X*_*i*,*g*_, *X*_*i*,*g*+1_ is set to *U*_*i*,*g*+1_, otherwise *X*_*i*,*g*_ is retained as *X*_*i*,*g*+1_. In (1), *f*_1_(•) is the CCR function and *f*_2_(•) is the NMI function, both of which are explained in the following section.

### Multiobjective evaluation

Two objective functions (fitness functions) are used to evaluate the values of target and trial vectors. The objective function can be divided into six steps based on MDR. Let *X* = (*x*_1_, .., *x*_*d*_) represent a gene–gene interaction, where *d* is the order number of gene–gene interactions. The genotype combinations between SNP factors (i.e., (*x*_1_, .., *x*_*d*_)) contain *d* ^3^ multifactor cells, each of which contains the total quantities of cases and controls for the corresponding genotype combination.

Step 1. Determine high or low risk within multifactor cells by using the training data.

Each multifactor cell is deemed high or low risk by evaluating the ratio between total quantities of cases and controls in that cell. A cell is deemed high-risk if *ratio* ≤ 1 and low risk otherwise. In the training data, ${\widehat{\theta }}_{a}$ represents a ratio value and is computed by (8) to provide a more accurate ratio to determine whether a cell is high or low risk. Thus, accurate objective evaluations can be improved when the total quantities of cases and controls are unbalanced. Equation (8) is expressed as follows:

8 $${\widehat{\theta }}_{a}=\frac{{n}_{+0}\times {n}_{a1}}{{n}_{+1}\times {n}_{a0}}$$

where *n*_*ab*_ is the total number of samples within the *a*th multifactor cell in the *b* outcome risk status in the training data, and *n*_+*b*_ represents the total number of samples in the *b* outcome risk status, where *b* = 1 for cases and 0 for controls.

Step 2. Determine high or low risk within multifactor cells by using the testing data.

To use the testing data to determine whether multifactor cells are high or low risk, the ratio *θ*_*a*_ is computed by (9), expressed as follows:

9 $${\theta }_{a}=\frac{{n}_{+0}\times {t}_{a1}}{{n}_{+1}\times {t}_{a0}}$$

where *t*_*ab*_ is the number of samples within the *a*th multifactor cell in the *b* outcome risk status in the testing data, where *b* = 1 for cases and 0 for controls. Both *n*_+0_ and *n*_+1_ are the same as in (8).

Step 3. Evaluate the true positive (*TP*), false positive (*FP*), false negative (*FN*), and true negative (*TN*) values by comparing the level of risk in multifactor cells as determined by the training and testing data.

A comparison of the risk level of a single cell as determined by training and testing data can be used to compute the *TP*, *FP*, *FN*, and *TN*. Thus, all multifactor cells can be reduced to four dimensions (*TP*, *FP*, *FN*, and *TN*). Equation (10) expresses the evaluation functions of the four dimensions as follows:

10 $$\begin{array}{c}TP=\sum _{a\in \{{\widehat{\theta }}_{a},{\theta }_{a}\ge 1\}}{t}_{a1}\cap {n}_{a1},FP=\sum _{a\in \{{\widehat{\theta }}_{a},{\theta }_{a}\ge 1\}}{t}_{a0}\cap {n}_{a0},\\ FN=\sum _{a\in \{{\widehat{\theta }}_{a},{\theta }_{a} < 1\}}{t}_{a1}\cap {n}_{a1},TN=\sum _{a\in \{{\widehat{\theta }}_{a},{\theta }_{a} < 1\}}{t}_{a0}\cap {n}_{a0}\end{array}$$

where *t*_*ab*_ is the number of samples within the *a*th multifactor cell in the *b* outcome risk status; *n*_+*b*_ is the total number of samples in the *b* outcome risk status, where *b* = 1 for cases and 0 for controls; *TP* is the number of correctly classified samples in the testing data within the high-risk range as determined by training data; *FP* is the number of incorrectly classified samples in the testing data within the low-risk range as determined by the training data; *FN* is the number of incorrectly classified samples in the testing data within the high-risk range as determined by the training data; *TN* is the number of correctly classified samples in the testing data within the low-risk range as determined by the training data.

Step 4. Evaluate the fitness functions of objectives.

Objective 1:

Objective 1 is the CCR (11), which is used to determine the proportion of correctly classified individuals. The CCR is computed using the *TP* ratio for cases and *TN* ratio for controls, where the maximum value indicates the optimal solution. Equation (11) is expressed as follows:

11 $$CCR=0.5\times (\frac{TP}{TP+FN}+\frac{TN}{FP+TN})$$

where *TP*, *FP*, *FN*, and *TN* are computed using (10).

Objective 2:

Bush *et al*. used the NMI to evaluate MDR. NMI is a measure of information transmission based on Shannon entropy, interpreted as the proportion of information contained in the row variable transferred or transmitted to the column variable; more concisely, it is the amount by which the model reduces our uncertainty about the true state^22^. In the 2 × 2 contingency table, the row entropy *H*(*x*), column entropy *H*(*y*), and conditional entropy *H*(*y*|*x*) are defined as (12), (13), and (14), respectively, and expressed as follows:

12 $$H(x)=-\sum _{i}{p}_{i}{\mathrm{log}}_{2}{p}_{i}$$

13 $$H(y)=-\sum _{j}{p}_{j}{\mathrm{log}}_{2}{p}_{j}$$

14 $$H(y|x)=\sum _{i}{p}_{i}[-\sum _{j}\frac{{p}_{ij}}{{p}_{i}}{\mathrm{log}}_{2}\frac{{p}_{ij}}{{p}_{i}}]$$

where *p*_*i*_ and *p*_*j*_ are the frequencies of the predicted and true class states, respectively, and *p*_*ij*_ is the joint probability. Thus, NMI is calculated as follows:

15 $$\begin{array}{c}NMI=\frac{H(y)-H(y|x)}{H(y)}\\ =\frac{\begin{array}{c}2\{(TP+FN+TN+FP){\mathrm{log}}_{2}(TP+FN+TN+FP)+TP{\mathrm{log}}_{2}TP+FN{\mathrm{log}}_{2}FN\\ +TN{\mathrm{log}}_{2}TN+FP{\mathrm{log}}_{2}FP-(TP+FP){\mathrm{log}}_{2}(TP+FP)-(TP+FN){\mathrm{log}}_{2}(TP+FN)\\ -(TN+FP){\mathrm{log}}_{2}(TN+FP)-(TN+FN){\mathrm{log}}_{2}(TN+FN)\}\end{array}}{\begin{array}{c}2\{(TP+FN+TN+FP){\mathrm{log}}_{2}(TP+FN+TN+FP)\\ -(TP+FN{)\mathrm{log}}_{{\rm{2}}}(TP+FN)-(TN+FP{)\mathrm{log}}_{{\rm{2}}}(TN+FP)\}\end{array}}\end{array}$$

where *TP*, *FP*, *FN*, and *TN* are computed using (10), with the maximum value indicating the optimal solution.

Step 5. Repeat steps 1–4 until all CV folds have been completed.

Step 6. Compute the averages of the CCR and NMI values in all CV folds.

### Illustrative example of MODEMDR

The supplemental material in this paper provides an example of how MODEMDR works.

### Parameter settings

The SNPRuler parameter is set to the default settings. The parameter “updateRatio” is set to 0.2, which is the step size used for updating a rule. MDR, DEMDR, and MODEMDR use the five-fold CV test. DEMDR and MODEMDR have the following four common parameters: population size (*pop-size*), generation size (*gen-size*), scaling factor (*F*), and crossover constant (*CR*). For the simulation datasets, the following values were set in all experiments: *pop-size* = 100, *gen-size* = 300, *F* = 0.5, and *CR* = 0.5. For the real datasets, the values were set as follows: *pop-size* = 500, *gen-size* = 1,000, *F* = 0.5, and *CR* = 0.5. The parameter settings were based on Price *et al*.^31^. For MODEMDR, the maximum size of the Pareto set is 20% of *pop-size*.

### Ethnics Statements

The protocol for the study was approved by the Committee on Human Research at WTCCC using the Affymetrix GeneChip 500 K Mapping Array Set^24^ for data review. All experiments were performed in accordance with WTCCC guidelines and regulations.

## Electronic supplementary material

Supplementary File
