# Publisher Correction: Multiobjective differential evolution-based multifactor dimensionality reduction for detecting gene–gene interactions

**DOI:** 10.1038/s41598-017-16210-x

**Published:** 2017-11-23

**Authors:** Cheng-Hong Yang, Li-Yeh Chuang, Yu-Da Lin

**Affiliations:** 1 Department of Electronic Engineering, National Kaohsiung University of Applied Sciences, Kaohsiung, 80778 Taiwan; 2 Graduate Institute of Clinical Medicine, Kaohsiung Medical University, Kaohsiung, 80708 Taiwan; 3 Department of Chemical Engineering and Institute of Biotechnology and Chemical Engineering, I-Shou University, Kaohsiung, 84004 Taiwan

*Scientific Reports* **7**:12869; doi:10.1038/s41598-017-12773-x; Article published online 09 October 2017

The original HTML version of this Article contained an error in the footnote of Table 2.

“^a^Chr chromosome; ^b^MODEMDR running time; time unit: comment = “Style change has been done. Please check.“hour (h); **optimal epistatic interaction; *top ten epistatic interactions”.

now reads:

“^a^Chr chromosome; ^b^MODEMDR running time; time unit: hour (h); **optimal epistatic interaction; *top ten epistatic interactions”.

This error has now been corrected in the HTML version of the Article; the PDF version was correct from the time of publication.

