# Supplementary material for: Multiobjective differential evolution-based multifactor dimensionality reduction for detecting gene–gene interactions
Source: Sci Rep. 2017 Oct 9;7:12869. doi: 10.1038/s41598-017-12773-x (PMC5634479; doi:10.1038/s41598-017-12773-x)
Supplement: Supplementary file 1 — Supplementary File [file 41598_2017_12773_MOESM1_ESM.doc]

**Multiobjective differential evolution-based multifactor dimensionality reduction for detecting gene–gene interactions**

Cheng-Hong Yang, Li-Yeh Chuang, Yu-Da Lin

**Supplementary information**

**Table 1.** Model with marginal effects.

| *MAF* = 0.1, Prevalence = 0.050, *h*^2^ = 0.031 | | | | *MAF* = 0.1, Prevalence = 0.026, *h*^2^ = 0.009 | | | |
| --- | --- | --- | --- | --- | --- | --- | --- |
| Model 1 | AA | Aa | aa | Model 5 | AA | Aa | aa |
| BB | 0.060 | 0.010 | 0.010 | BB | 0.030 | 0.010 | 0.020 |
| Bb | 0.010 | 0.208 | 0.208 | Bb | 0.010 | 0.090 | 0.050 |
| bb | 0.010 | 0.208 | 0.208 | bb | 0.020 | 0.050 | 0.070 |
| *MAF* = 0.1, Prevalence = 0.050, *h*^2^ = 0.014 | | | | *MAF* = 0.1, Prevalence = 0.017, *h*^2^ = 0.008 | | | |
| Model 2 | AA | Aa | aa | Model 6 | AA | Aa | aa |
| BB | 0.061 | 0.017 | 0.017 | BB | 0.020 | 0.007 | 0.003 |
| Bb | 0.017 | 0.136 | 0.136 | Bb | 0.005 | 0.070 | 0.080 |
| bb | 0.017 | 0.136 | 0.136 | bb | 0.020 | 0.001 | 0.090 |
| *MAF* = 0.1, Prevalence = 0.050, *h*^2^ = 0.01 | | | | *MAF* = 0.2, Prevalence = 0.052, *h*^2^ = 0.005 | | | |
| Model 3 | AA | Aa | aa | Model 7 | AA | Aa | aa |
| BB | 0.060 | 0.021 | 0.021 | BB | 0.044 | 0.069 | 0.042 |
| Bb | 0.021 | 0.116 | 0.116 | Bb | 0.066 | 0.021 | 0.073 |
| bb | 0.021 | 0.116 | 0.116 | bb | 0.073 | 0.007 | 0.054 |
| *MAF* = 0.1, Prevalence = 0.046, *h*^2^ = 0.016 | | | | *MAF* = 0.4, Prevalence = 0.048, *h*^2^ = 0.006 | | | |
| Model 4 | AA | Aa | aa | Model 8 | AA | Aa | aa |
| BB | 0.030 | 0.090 | 0.070 | BB | 0.025 | 0.051 | 0.087 |
| Bb | 0.080 | 0.010 | 0.040 | Bb | 0.061 | 0.044 | 0.029 |
| bb | 0.090 | 0.010 | 0.000 | bb | 0.057 | 0.051 | 0.016 |

**Table 2. Models 1 to 10 without marginal effects**

| *h*^2^ = 0.4, *MAF* = 0.2 | | | | *h*^2^ = 0.4, *MAF* = 0.4 | | | |
| --- | --- | --- | --- | --- | --- | --- | --- |
| Epi.1 | AA | Aa | aa | Epi.6 | AA | Aa | aa |
| BB | 0.486 | 0.960 | 0.538 | BB | 0.077 | 0.656 | 0.880 |
| Bb | 0.947 | 0.004 | 0.811 | Bb | 0.892 | 0.235 | 0.312 |
| bb | 0.640 | 0.606 | 0.909 | bb | 0.174 | 0.842 | 0.106 |
| *h*^2^ = 0.4, *MAF* = 0.2 | | | | *h*^2^ = 0.4, *MAF* = 0.4 | | | |
| Epi.2 | AA | Aa | aa | Epi.7 | AA | Aa | aa |
| BB | 0.469 | 0.956 | 0.697 | BB | 0.895 | 0.323 | 0.161 |
| Bb | 0.945 | 0.019 | 0.585 | Bb | 0.068 | 0.728 | 0.806 |
| bb | 0.786 | 0.407 | 0.013 | bb | 0.925 | 0.233 | 0.362 |
| *h*^2^ = 0.4, *MAF* = 0.2 | | | | *h*^2^ = 0.4, *MAF* = 0.4 | | | |
| Epi.3 | AA | Aa | aa | Epi.8 | AA | Aa | aa |
| BB | 0.498 | 0.954 | 0.786 | BB | 0.805 | 0.251 | 0.085 |
| Bb | 0.978 | 0.038 | 0.428 | Bb | 0.002 | 0.668 | 0.638 |
| bb | 0.590 | 0.821 | 0.380 | bb | 0.830 | 0.079 | 0.542 |
| *h*^2^ = 0.4, *MAF* = 0.2 | | | | *h*^2^ = 0.4, *MAF* = 0.4 | | | |
| Epi.4 | AA | Aa | aa | Epi.9 | AA | Aa | aa |
| BB | 0.505 | 0.988 | 0.624 | BB | 0.307 | 0.682 | 0.958 |
| Bb | 0.945 | 0.085 | 0.807 | Bb | 0.997 | 0.390 | 0.281 |
| bb | 0.969 | 0.116 | 0.159 | bb | 0.012 | 0.990 | 0.698 |
| *h*^2^ = 0.4, *MAF* = 0.2 | | | | *h*^2^ = 0.4, *MAF* = 0.4 | | | |
| Epi.5 | AA | Aa | aa | Epi.10 | AA | Aa | aa |
| BB | 0.486 | 0.963 | 0.512 | BB | 0.083 | 0.891 | 0.037 |
| Bb | 0.941 | 0.006 | 0.899 | Bb | 0.619 | 0.271 | 0.691 |
| bb | 0.691 | 0.541 | 0.614 | bb | 0.853 | 0.079 | 0.742 |

**Table 2. *Cont*.**

| *h*^2^ = 0.3, *MAF* = 0.2 | | | | *h*^2^ = 0.3, *MAF* = 0.4 | | | |
| --- | --- | --- | --- | --- | --- | --- | --- |
| Epi.11 | AA | Aa | aa | Epi.16 | AA | Aa | aa |
| BB | 0.500 | 0.926 | 0.615 | BB | 0.891 | 0.362 | 0.480 |
| Bb | 0.895 | 0.131 | 0.647 | Bb | 0.213 | 0.829 | 0.601 |
| bb | 0.858 | 0.160 | 0.999 | bb | 0.925 | 0.267 | 0.685 |
| *h*^2^ = 0.3, *MAF* = 0.2 | | | | *h*^2^ = 0.3, *MAF* = 0.4 | | | |
| Epi.12 | AA | Aa | aa | Epi.17 | AA | Aa | aa |
| BB | 0.413 | 0.851 | 0.535 | BB | 0.077 | 0.689 | 0.417 |
| Bb | 0.831 | 0.008 | 0.580 | Bb | 0.763 | 0.150 | 0.491 |
| bb | 0.692 | 0.268 | 0.736 | bb | 0.196 | 0.657 | 0.247 |
| *h*^2^ = 0.3, *MAF* = 0.2 | | | | *h*^2^ = 0.3, *MAF* = 0.4 | | | |
| Epi.13 | AA | Aa | aa | Epi.18 | AA | Aa | aa |
| BB | 0.455 | 0.848 | 0.897 | BB | 0.132 | 0.793 | 0.274 |
| Bb | 0.890 | 0.088 | 0.016 | Bb | 0.799 | 0.213 | 0.514 |
| bb | 0.562 | 0.686 | 0.467 | bb | 0.255 | 0.528 | 0.793 |
| *h*^2^ = 0.3, *MAF* = 0.2 | | | | *h*^2^ = 0.3, *MAF* = 0.4 | | | |
| Epi.14 | AA | Aa | aa | Epi.19 | AA | Aa | aa |
| BB | 0.609 | 0.980 | 0.980 | BB | 0.611 | 0.104 | 0.759 |
| Bb | 0.993 | 0.300 | 0.275 | Bb | 0.180 | 0.674 | 0.019 |
| bb | 0.876 | 0.483 | 0.683 | bb | 0.532 | 0.189 | 0.681 |
| *h*^2^ = 0.3, *MAF* = 0.2 | | | | *h*^2^ = 0.3, *MAF* = 0.4 | | | |
| Epi.15 | AA | Aa | aa | Epi.20 | AA | Aa | aa |
| BB | 0.486 | 0.963 | 0.512 | BB | 0.091 | 0.827 | 0.863 |
| Bb | 0.941 | 0.006 | 0.899 | Bb | 0.869 | 0.393 | 0.415 |
| bb | 0.691 | 0.541 | 0.614 | bb | 0.738 | 0.508 | 0.363 |

**Table 2. *Cont*.**

| *h*^2^ = 0.2, *MAF* = 0.2 | | | | *h*^2^ = 0.2, *MAF* = 0.4 | | | |
| --- | --- | --- | --- | --- | --- | --- | --- |
| Epi.21 | AA | Aa | aa | Epi.26 | AA | Aa | aa |
| BB | 0.428 | 0.757 | 0.812 | BB | 0.356 | 0.891 | 0.809 |
| Bb | 0.788 | 0.132 | 0.044 | Bb | 0.955 | 0.508 | 0.611 |
| bb | 0.559 | 0.548 | 0.373 | bb | 0.617 | 0.755 | 0.63 |
| *h*^2^ = 0.2, *MAF* = 0.2 | | | | *h*^2^ = 0.2, *MAF* = 0.4 | | | |
| Epi.22 | AA | Aa | aa | Epi.27 | AA | Aa | aa |
| BB | 0.507 | 0.842 | 0.605 | BB | 0.086 | 0.536 | 0.641 |
| Bb | 0.845 | 0.162 | 0.629 | Bb | 0.677 | 0.275 | 0.096 |
| bb | 0.581 | 0.678 | 0.729 | bb | 0.219 | 0.413 | 0.712 |
| *h*^2^ = 0.2, *MAF* = 0.2 | | | | *h*^2^ = 0.2, *MAF* = 0.4 | | | |
| Epi.23 | AA | Aa | aa | Epi.28 | AA | Aa | aa |
| BB | 0.577 | 0.247 | 0.428 | BB | 0.855 | 0.339 | 0.772 |
| Bb | 0.227 | 0.928 | 0.578 | Bb | 0.513 | 0.651 | 0.607 |
| bb | 0.586 | 0.262 | 0.158 | bb | 0.25 | 0.999 | 0.154 |
| *h*^2^ = 0.2, *MAF* = 0.2 | | | | *h*^2^ = 0.2, *MAF* = 0.4 | | | |
| Epi.24 | AA | Aa | aa | Epi.29 | AA | Aa | aa |
| BB | 0.340 | 0.637 | 0.654 | BB | 0.506 | 0.838 | 0.024 |
| Bb | 0.689 | 0.017 | 0.041 | Bb | 0.603 | 0.454 | 0.957 |
| bb | 0.242 | 0.866 | 0.403 | bb | 0.729 | 0.427 | 0.753 |
| *h*^2^ = 0.2, *MAF* = 0.2 | | | | *h*^2^ = 0.2, *MAF* = 0.4 | | | |
| Epi.25 | AA | Aa | aa | Epi.30 | AA | Aa | aa |
| BB | 0.387 | 0.726 | 0.734 | BB | 0.393 | 0.764 | 0.664 |
| Bb | 0.749 | 0.09 | 0.034 | Bb | 0.85 | 0.398 | 0.733 |
| bb | 0.551 | 0.401 | 0.724 | bb | 0.406 | 0.927 | 0.147 |

**Table 2. *Cont*.**

| *h*^2^ = 0.1, *MAF* = 0.2 | | | | *h*^2^ = 0.1, *MAF* = 0.4 | | | |
| --- | --- | --- | --- | --- | --- | --- | --- |
| Epi.31 | AA | Aa | aa | Epi.36 | AA | Aa | aa |
| BB | 0.463 | 0.703 | 0.431 | BB | 0.137 | 0.484 | 0.187 |
| Bb | 0.653 | 0.277 | 0.806 | Bb | 0.482 | 0.166 | 0.365 |
| bb | 0.83 | 0.008 | 0.129 | bb | 0.193 | 0.361 | 0.43 |
| *h*^2^ = 0.1, *MAF* = 0.2 | | | | *h*^2^ = 0.1, *MAF* = 0.4 | | | |
| Epi.32 | AA | Aa | aa | Epi.37 | AA | Aa | aa |
| BB | 0.319 | 0.507 | 0.569 | BB | 0.469 | 0.198 | 0.754 |
| Bb | 0.553 | 0.105 | 0.045 | Bb | 0.337 | 0.502 | 0.141 |
| bb | 0.203 | 0.777 | 0.28 | bb | 0.339 | 0.453 | 0.285 |
| *h*^2^ = 0.1, *MAF* = 0.2 | | | | *h*^2^ = 0.1, *MAF* = 0.4 | | | |
| Epi.33 | AA | Aa | aa | Epi.38 | AA | Aa | aa |
| BB | 0.627 | 0.393 | 0.335 | BB | 0.478 | 0.311 | 0.864 |
| Bb | 0.396 | 0.779 | 0.953 | Bb | 0.387 | 0.579 | 0.263 |
| bb | 0.174 | 0.842 | 0.106 | bb | 0.634 | 0.436 | 0.138 |
| *h*^2^ = 0.1, *MAF* = 0.2 | | | | *h*^2^ = 0.1, *MAF* = 0.4 | | | |
| Epi.34 | AA | Aa | aa | Epi.39 | AA | Aa | aa |
| BB | 0.297 | 0.54 | 0.441 | BB | 0.068 | 0.299 | 0.017 |
| Bb | 0.541 | 0.072 | 0.278 | Bb | 0.289 | 0.044 | 0.285 |
| bb | 0.434 | 0.293 | 0.228 | bb | 0.048 | 0.262 | 0.174 |
| *h*^2^ = 0.1, *MAF* = 0.2 | | | | *h*^2^ = 0.1, *MAF* = 0.4 | | | |
| Epi.35 | AA | Aa | aa | Epi.40 | AA | Aa | aa |
| BB | 0.332 | 0.562 | 0.573 | BB | 0.539 | 0.12 | 0.258 |
| Bb | 0.583 | 0.112 | 0.147 | Bb | 0.165 | 0.378 | 0.325 |
| bb | 0.399 | 0.496 | 0.033 | bb | 0.123 | 0.426 | 0.276 |

**Table 2. *Cont*.**

| *h*^2^ = 0.05, *MAF* = 0.2 | | | | *h*^2^ = 0.05, *MAF* = 0.4 | | | |
| --- | --- | --- | --- | --- | --- | --- | --- |
| Epi.41 | AA | Aa | aa | Epi.46 | AA | Aa | aa |
| BB | 0.492 | 0.664 | 0.481 | BB | 0.002 | 0.155 | 0.214 |
| Bb | 0.642 | 0.33 | 0.746 | Bb | 0.199 | 0.071 | 0.022 |
| bb | 0.656 | 0.396 | 0 | bb | 0.081 | 0.122 | 0.135 |
| *h*^2^ = 0.05, *MAF* = 0.2 | | | | *h*^2^ = 0.05, *MAF* = 0.4 | | | |
| Epi.42 | AA | Aa | aa | Epi.47 | AA | Aa | aa |
| BB | 0.499 | 0.639 | 0.765 | BB | 0.188 | 0.02 | 0.171 |
| Bb | 0.666 | 0.389 | 0.083 | Bb | 0.032 | 0.174 | 0.059 |
| bb | 0.543 | 0.527 | 0.953 | bb | 0.134 | 0.087 | 0.092 |
| *h*^2^ = 0.05, *MAF* = 0.2 | | | | *h*^2^ = 0.05, *MAF* = 0.4 | | | |
| Epi.43 | AA | Aa | aa | Epi.48 | AA | Aa | aa |
| BB | 0.212 | 0.35 | 0.116 | BB | 0.005 | 0.179 | 0.251 |
| Bb | 0.336 | 0.054 | 0.495 | Bb | 0.211 | 0.1 | 0.026 |
| bb | 0.227 | 0.273 | 0.495 | bb | 0.156 | 0.098 | 0.156 |
| *h*^2^ = 0.05, *MAF* = 0.2 | | | | *h*^2^ = 0.05, *MAF* = 0.4 | | | |
| Epi.44 | AA | Aa | aa | Epi.49 | AA | Aa | aa |
| BB | 0.805 | 0.683 | 0.638 | BB | 0.174 | 0.321 | 0.154 |
| Bb | 0.657 | 0.936 | 0.989 | Bb | 0.223 | 0.254 | 0.245 |
| bb | 0.85 | 0.564 | 0.866 | bb | 0.448 | 0.025 | 0.424 |
| *h*^2^ = 0.05, *MAF* = 0.2 | | | | *h*^2^ = 0.05, *MAF* = 0.4 | | | |
| Epi.45 | AA | Aa | aa | Epi.50 | AA | Aa | aa |
| BB | 0.638 | 0.488 | 0.383 | BB | 0.098 | 0.219 | 0.302 |
| Bb | 0.464 | 0.765 | 0.957 | Bb | 0.302 | 0.126 | 0.121 |
| bb | 0.58 | 0.562 | 0.719 | bb | 0.053 | 0.308 | 0.136 |

**Table 2. *Cont*.**

| *h*^2^ = 0.025, *MAF* = 0.2 | | | | *h*^2^ = 0.025, *MAF* = 0.4 | | | |
| --- | --- | --- | --- | --- | --- | --- | --- |
| Epi.51 | AA | Aa | aa | Epi.56 | AA | Aa | aa |
| BB | 0.495 | 0.415 | 0.657 | BB | 0.002 | 0.155 | 0.214 |
| Bb | 0.429 | 0.616 | 0.121 | Bb | 0.199 | 0.071 | 0.022 |
| bb | 0.552 | 0.331 | 0.419 | bb | 0.081 | 0.122 | 0.135 |
| *h*^2^ = 0.025, *MAF* = 0.2 | | | | *h*^2^ = 0.025, *MAF* = 0.4 | | | |
| Epi.52 | AA | Aa | aa | Epi.57 | AA | Aa | aa |
| BB | 0.592 | 0.691 | 0.743 | BB | 0.188 | 0.02 | 0.171 |
| Bb | 0.712 | 0.493 | 0.419 | Bb | 0.032 | 0.174 | 0.059 |
| bb | 0.58 | 0.746 | 0.504 | bb | 0.134 | 0.087 | 0.092 |
| *h*^2^ = 0.025, *MAF* = 0.2 | | | | *h*^2^ = 0.025, *MAF* = 0.4 | | | |
| Epi.53 | AA | Aa | aa | Epi.58 | AA | Aa | aa |
| BB | 0.108 | 0.194 | 0.186 | BB | 0.005 | 0.179 | 0.251 |
| Bb | 0.196 | 0.037 | 0.045 | Bb | 0.211 | 0.1 | 0.026 |
| bb | 0.172 | 0.073 | 0.13 | bb | 0.156 | 0.098 | 0.156 |
| *h*^2^ = 0.025, *MAF* = 0.2 | | | | *h*^2^ = 0.025, *MAF* = 0.4 | | | |
| Epi.54 | AA | Aa | aa | Epi.59 | AA | Aa | aa |
| BB | 0.112 | 0.186 | 0.128 | BB | 0.174 | 0.321 | 0.154 |
| Bb | 0.193 | 0.024 | 0.138 | Bb | 0.223 | 0.254 | 0.245 |
| bb | 0.079 | 0.236 | 0.251 | bb | 0.448 | 0.025 | 0.424 |
| *h*^2^ = 0.025, *MAF* = 0.2 | | | | *h*^2^ = 0.025, *MAF* = 0.4 | | | |
| Epi.55 | AA | Aa | aa | Epi.60 | AA | Aa | aa |
| BB | 0.272 | 0.192 | 0.185 | BB | 0.098 | 0.219 | 0.302 |
| Bb | 0.172 | 0.367 | 0.39 | Bb | 0.302 | 0.126 | 0.121 |
| bb | 0.345 | 0.069 | 0.005 | bb | 0.053 | 0.308 | 0.136 |

**Chromosome 1**

| 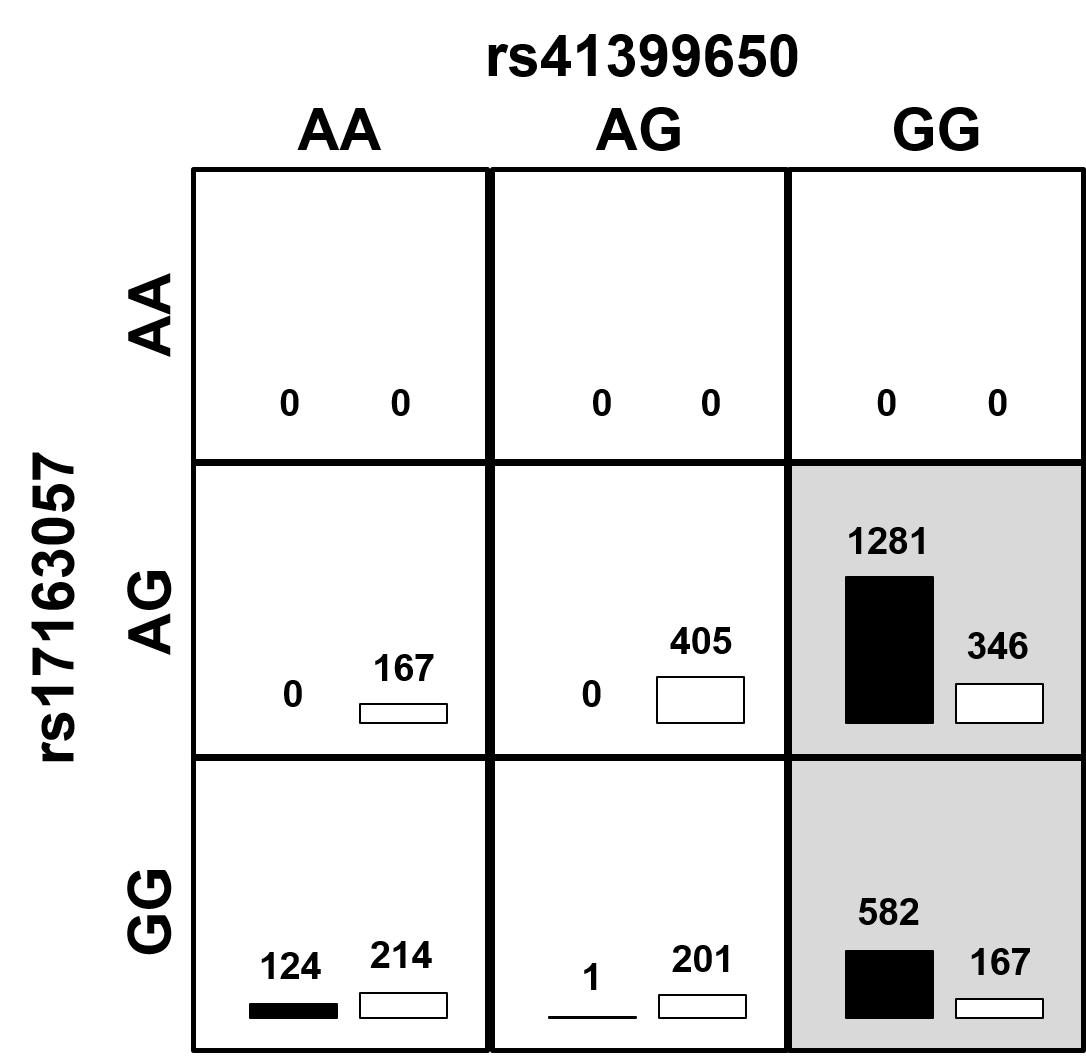 | 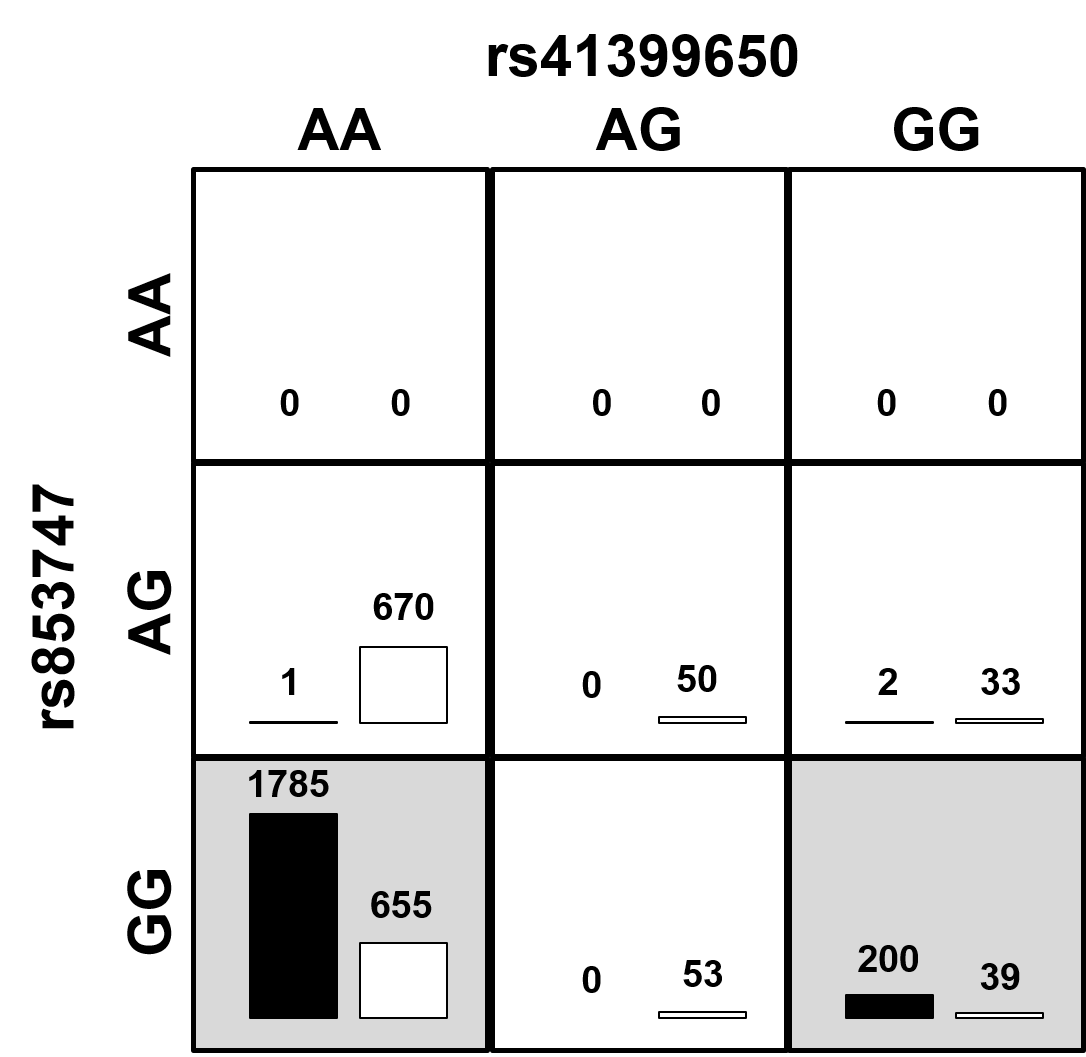 |
| --- | --- |
| 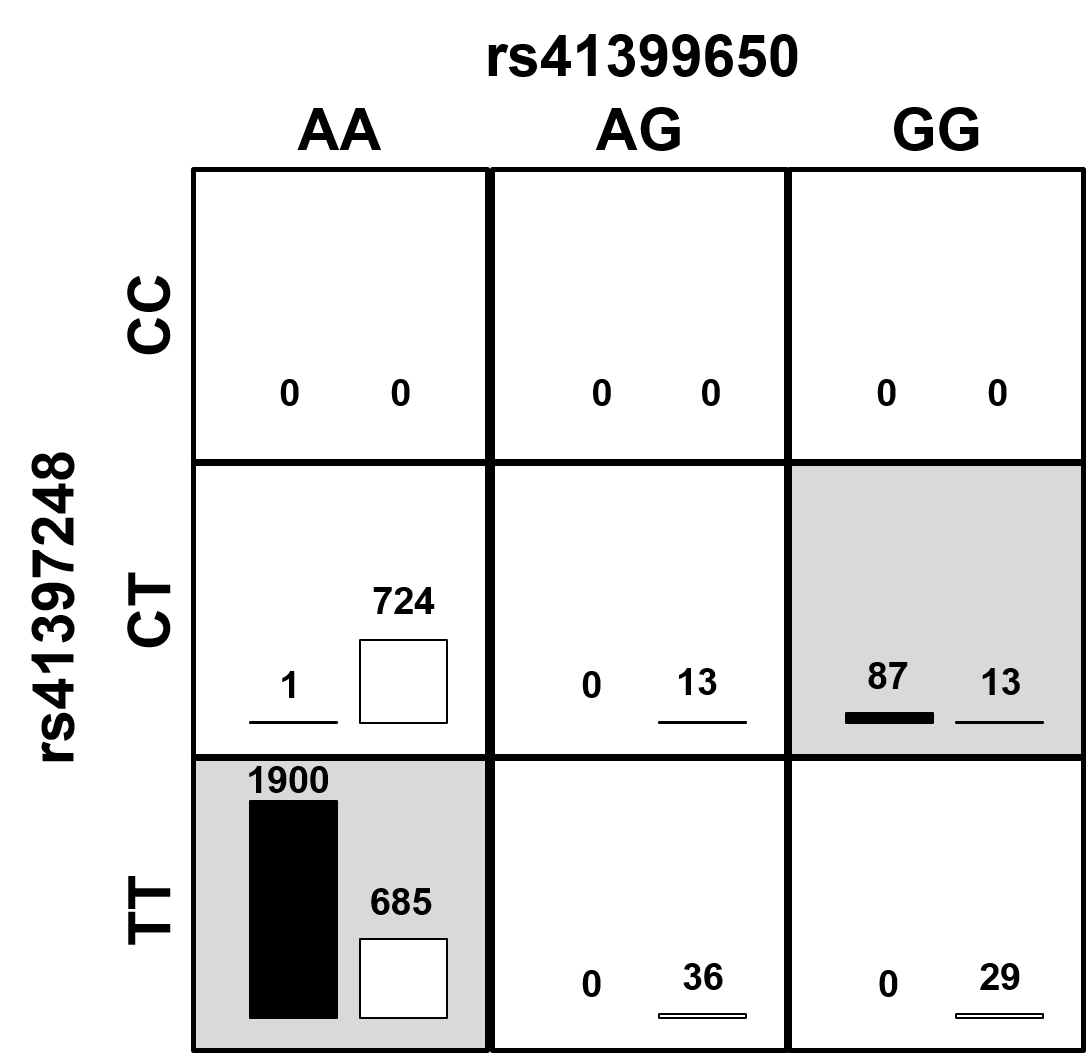 | |

**Figure 1.** Genotype counts for the SNP pairs in CAD relative to the most common double homozygote genotype in chromosomes on WTCCC data.

**Chromosome 2**


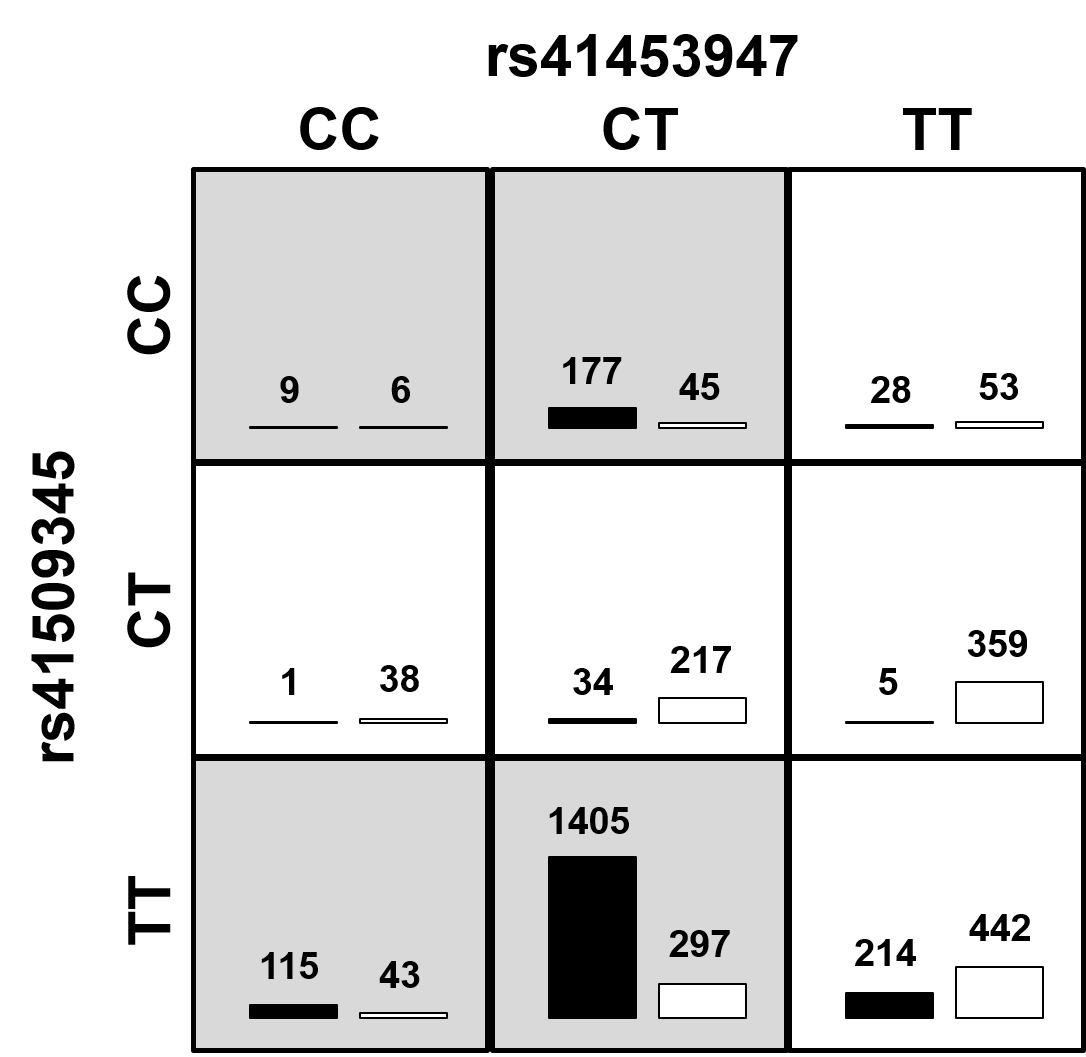


**Chromosome 3**

| 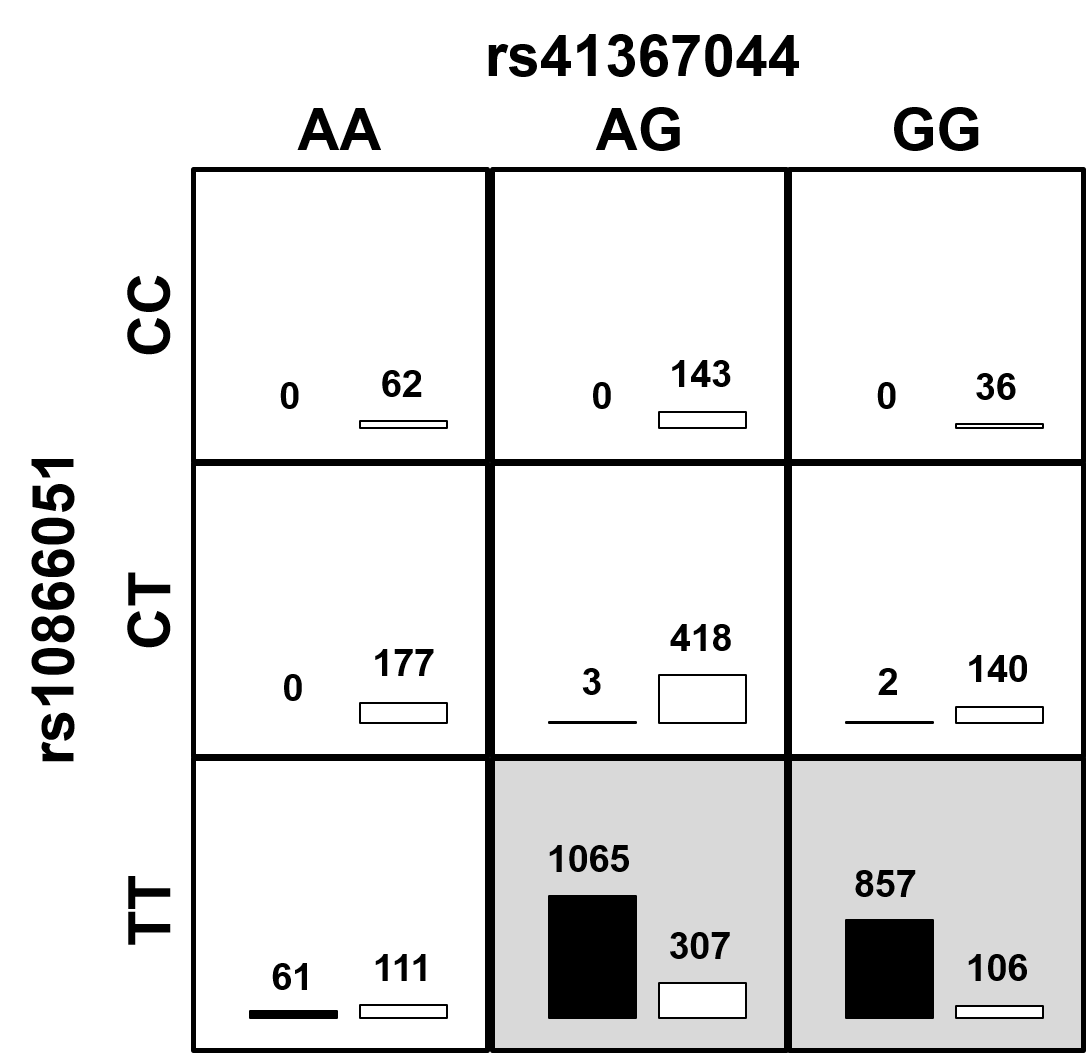 | 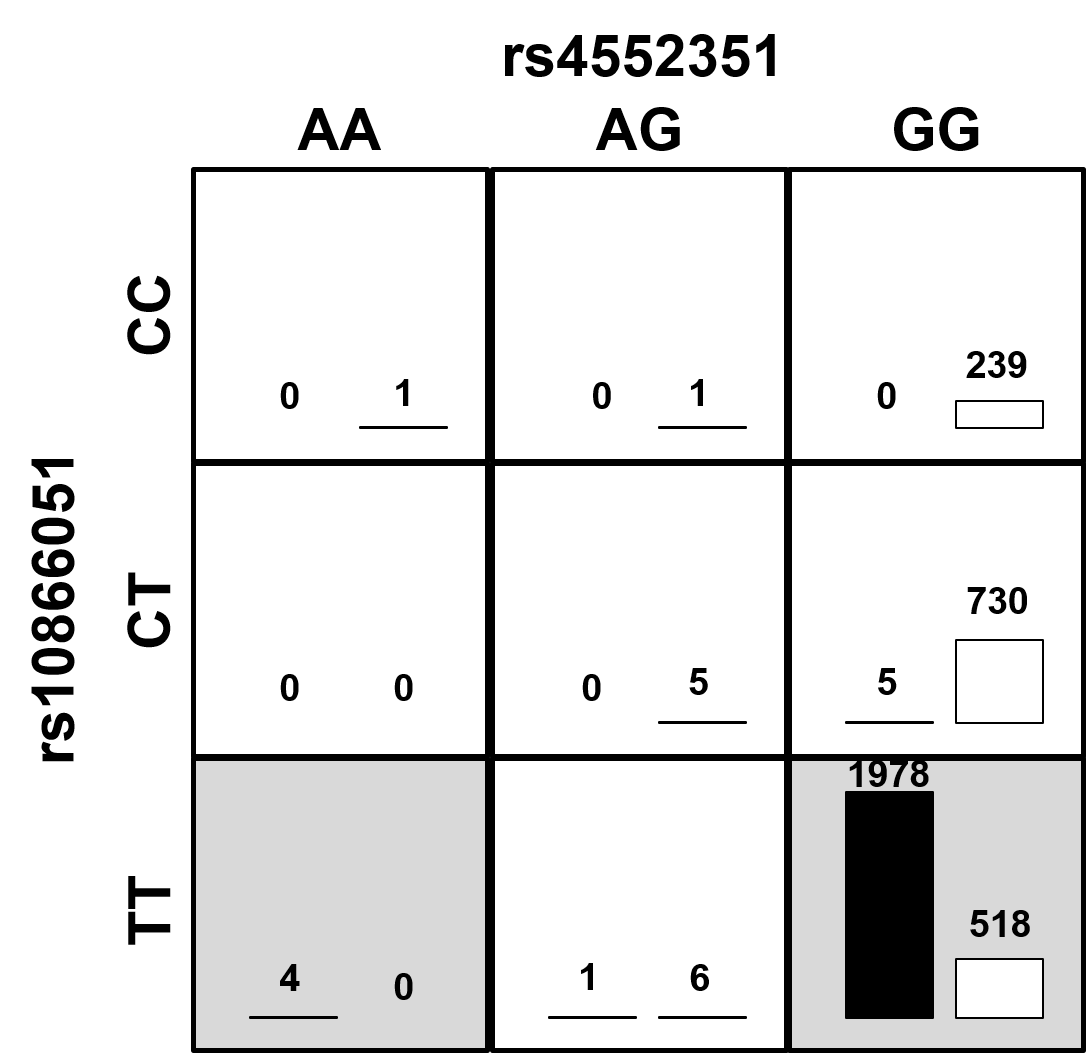 |
| --- | --- |
| 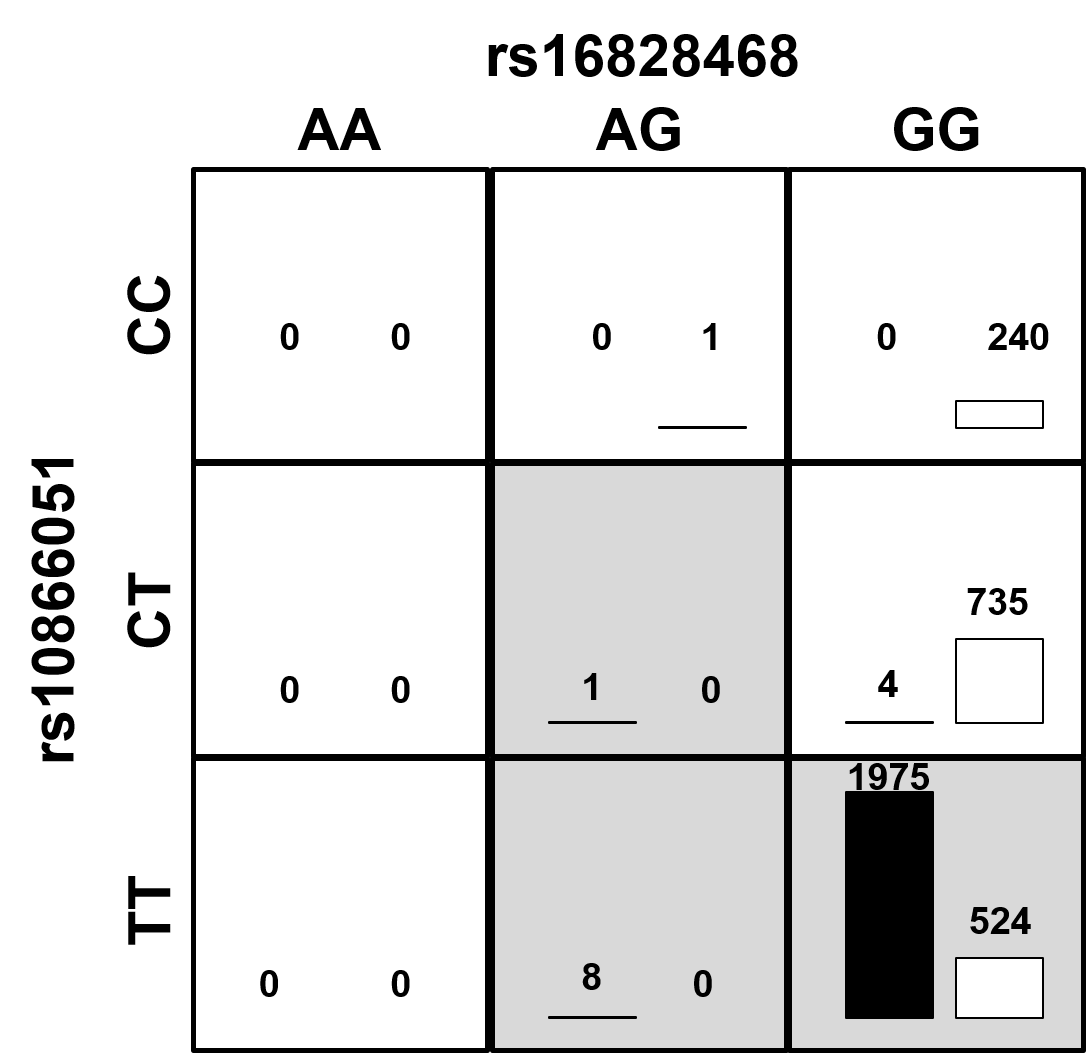 | |

**Chromosome 4**


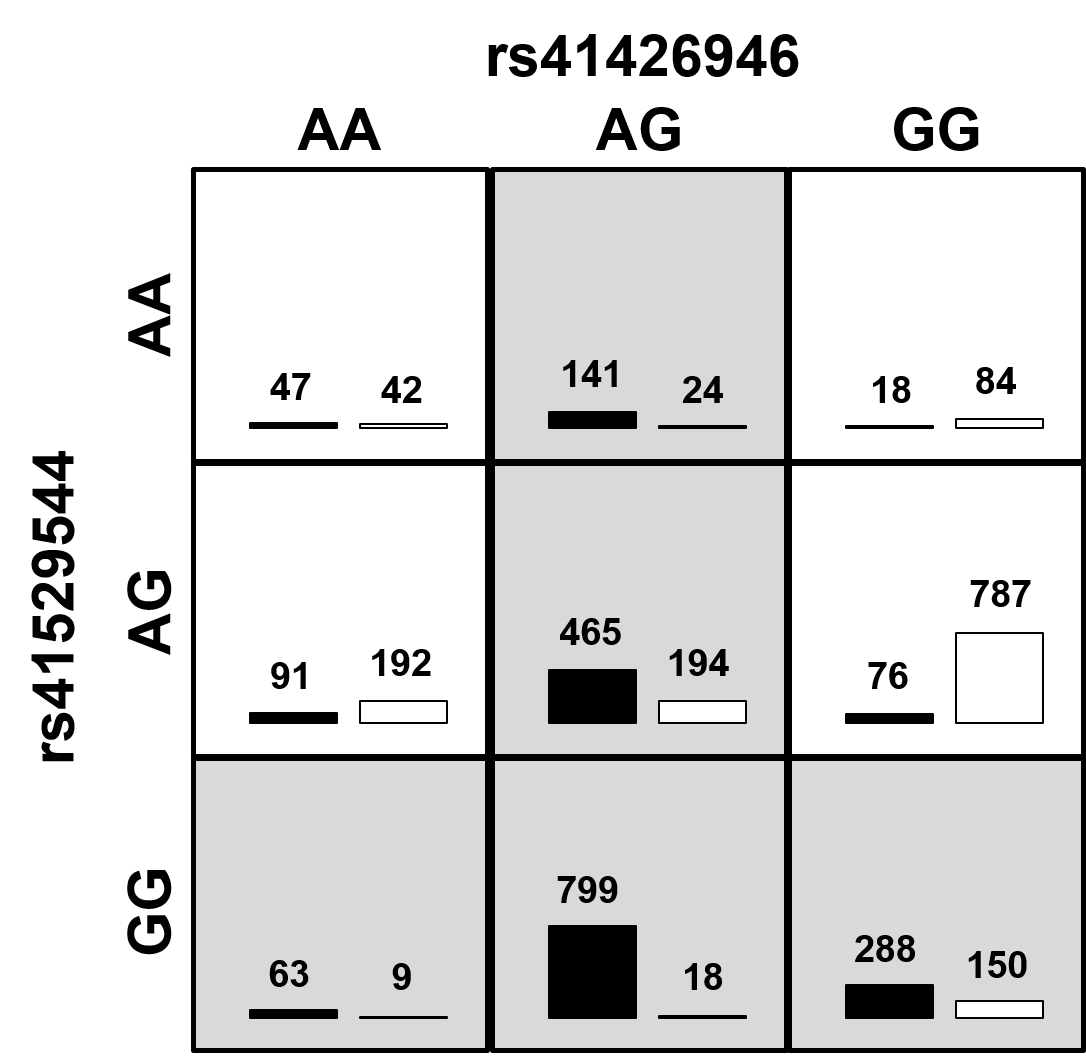


**Chromosome 5**


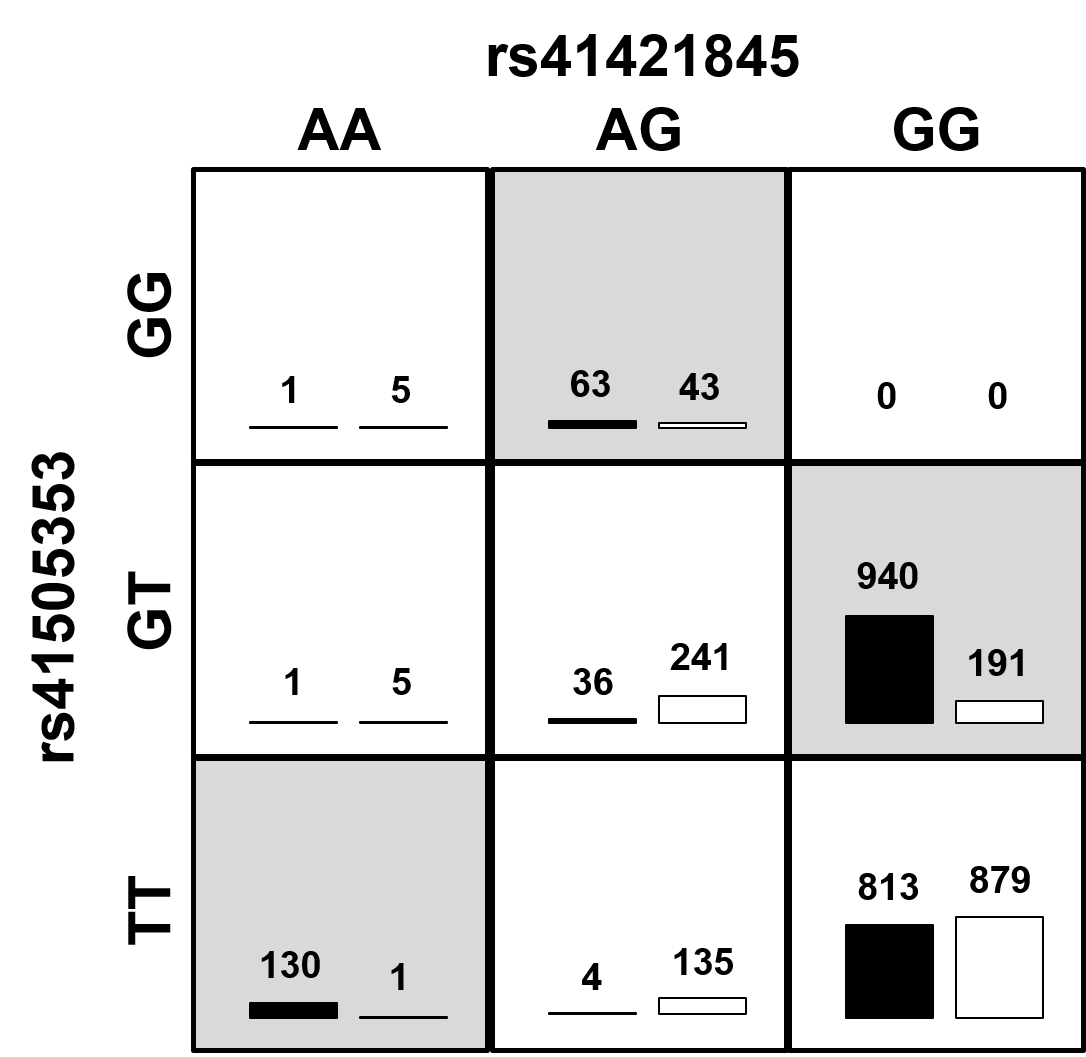


**Chromosome 6**

| 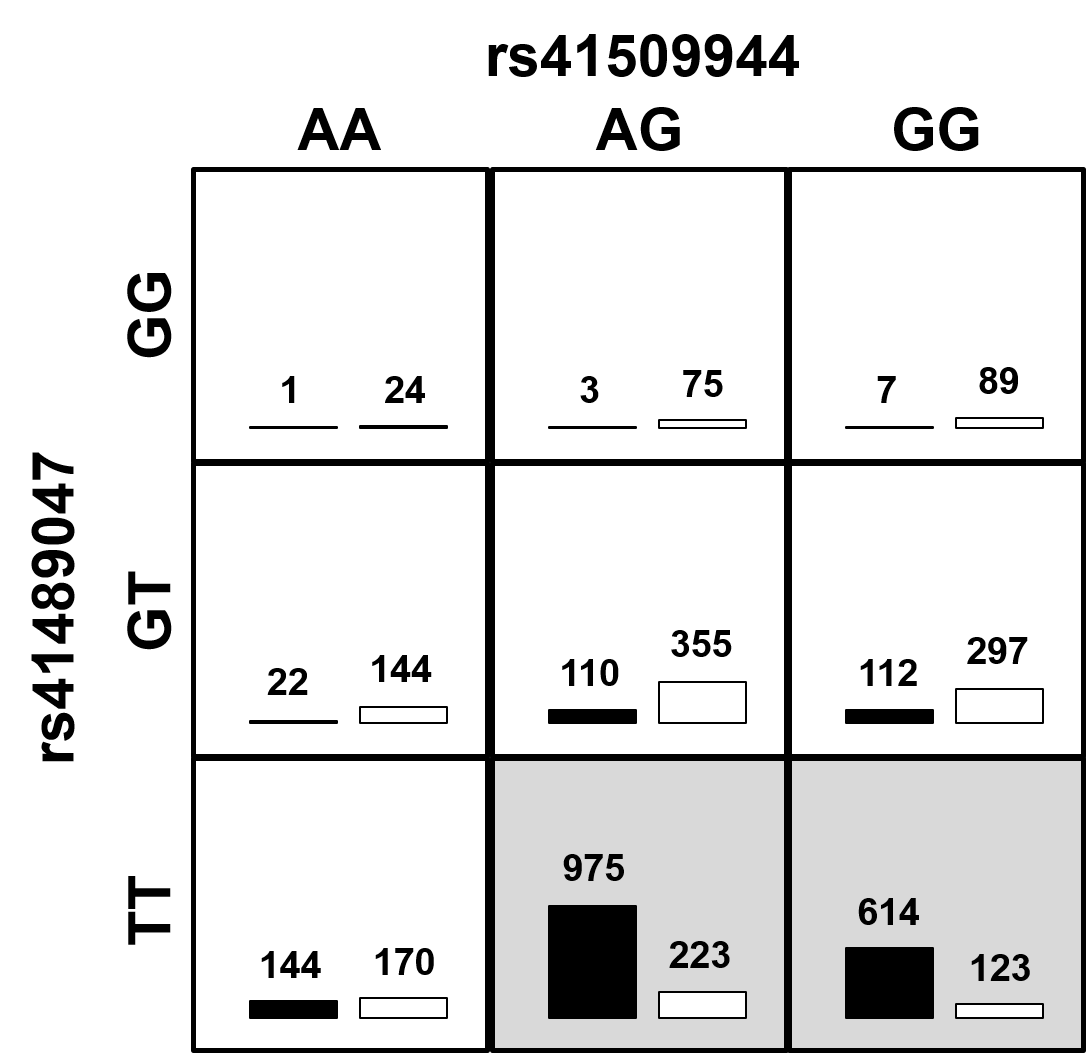 | 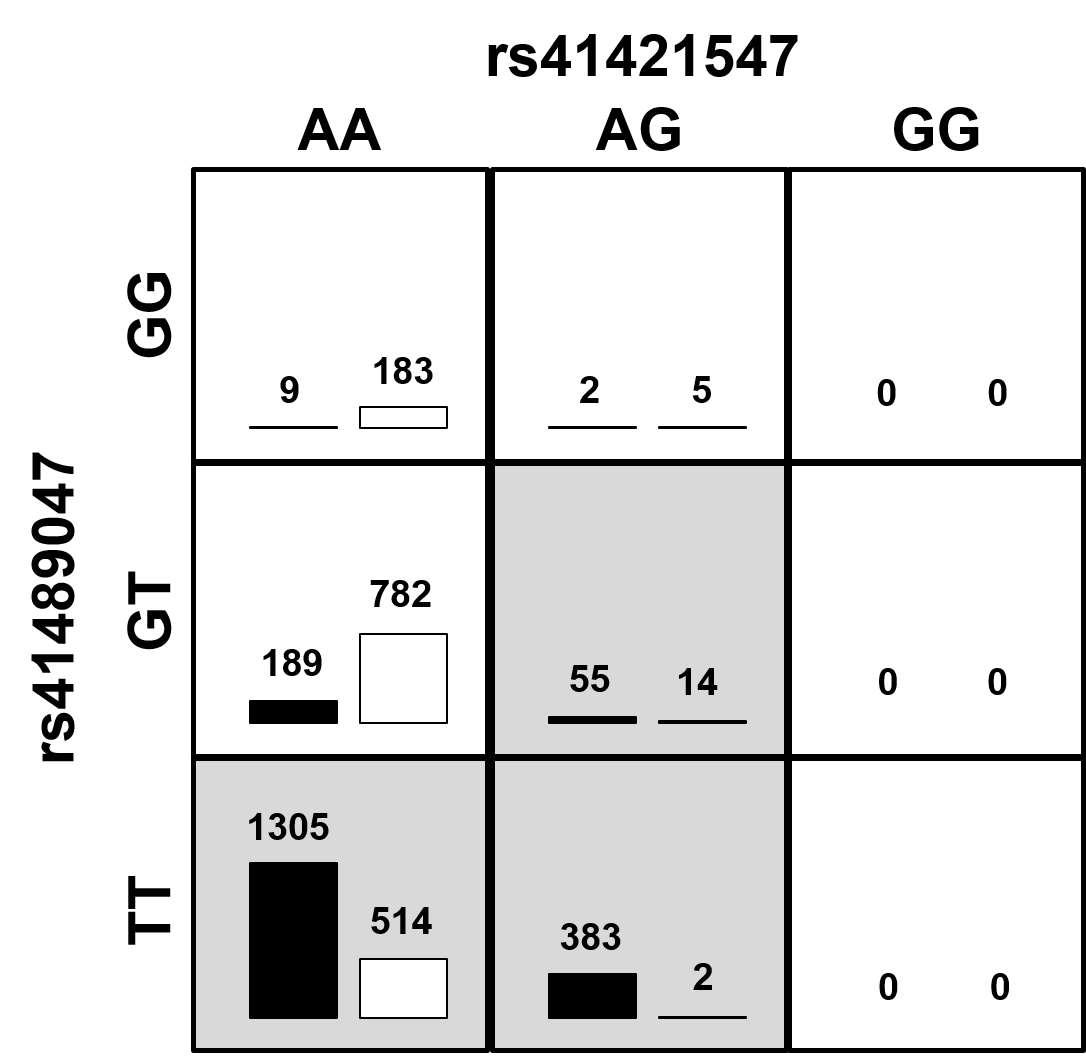 |
| --- | --- |
| 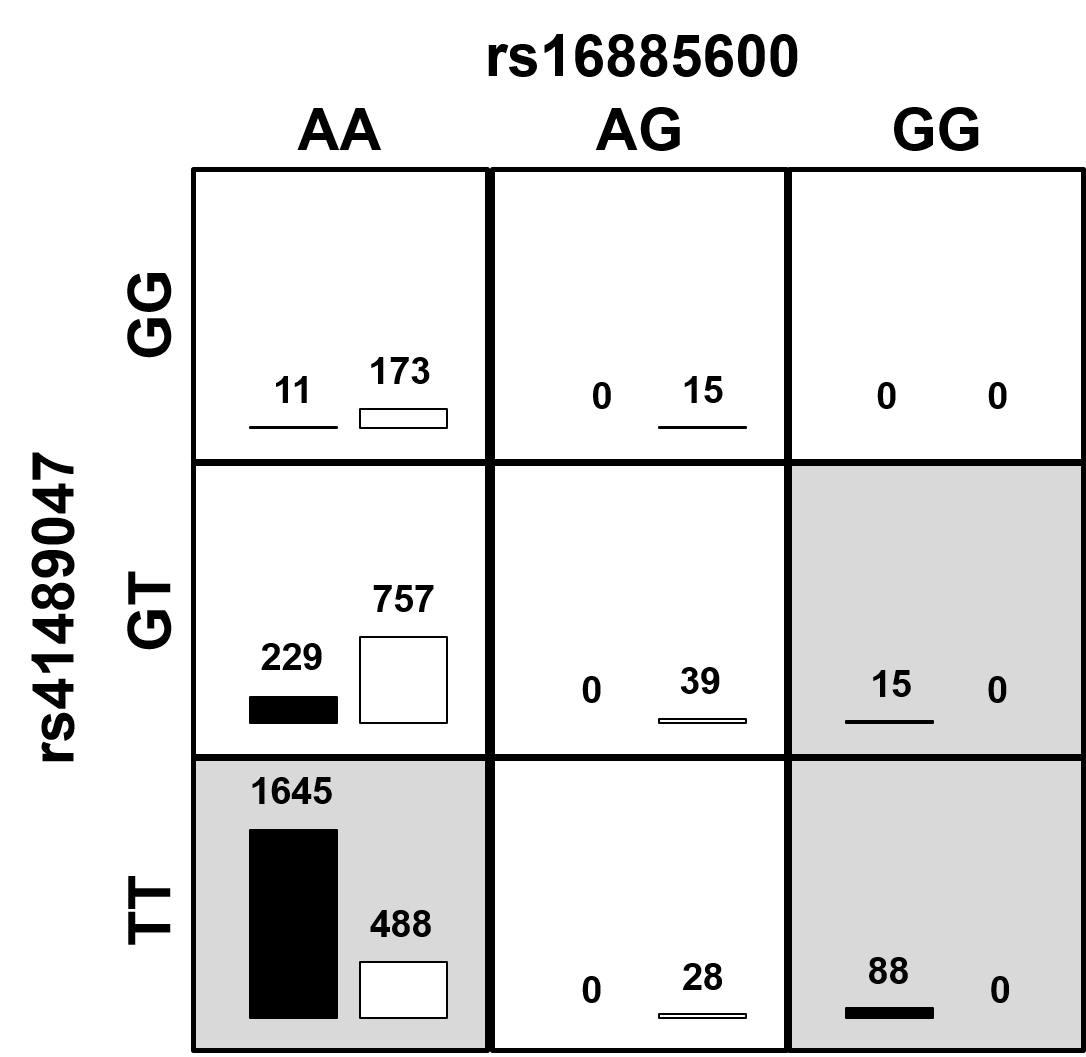 | |

**Chromosome 7**

| 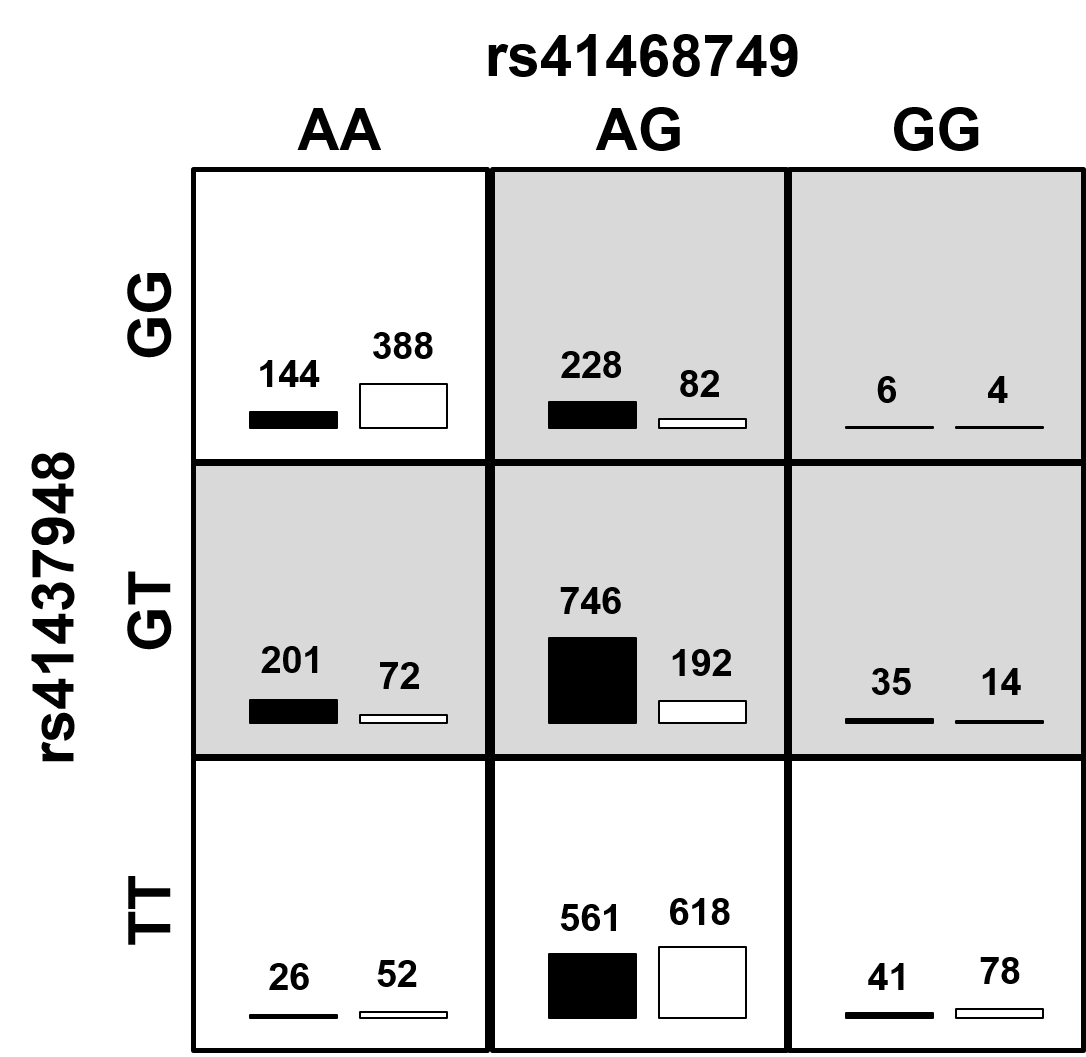 | 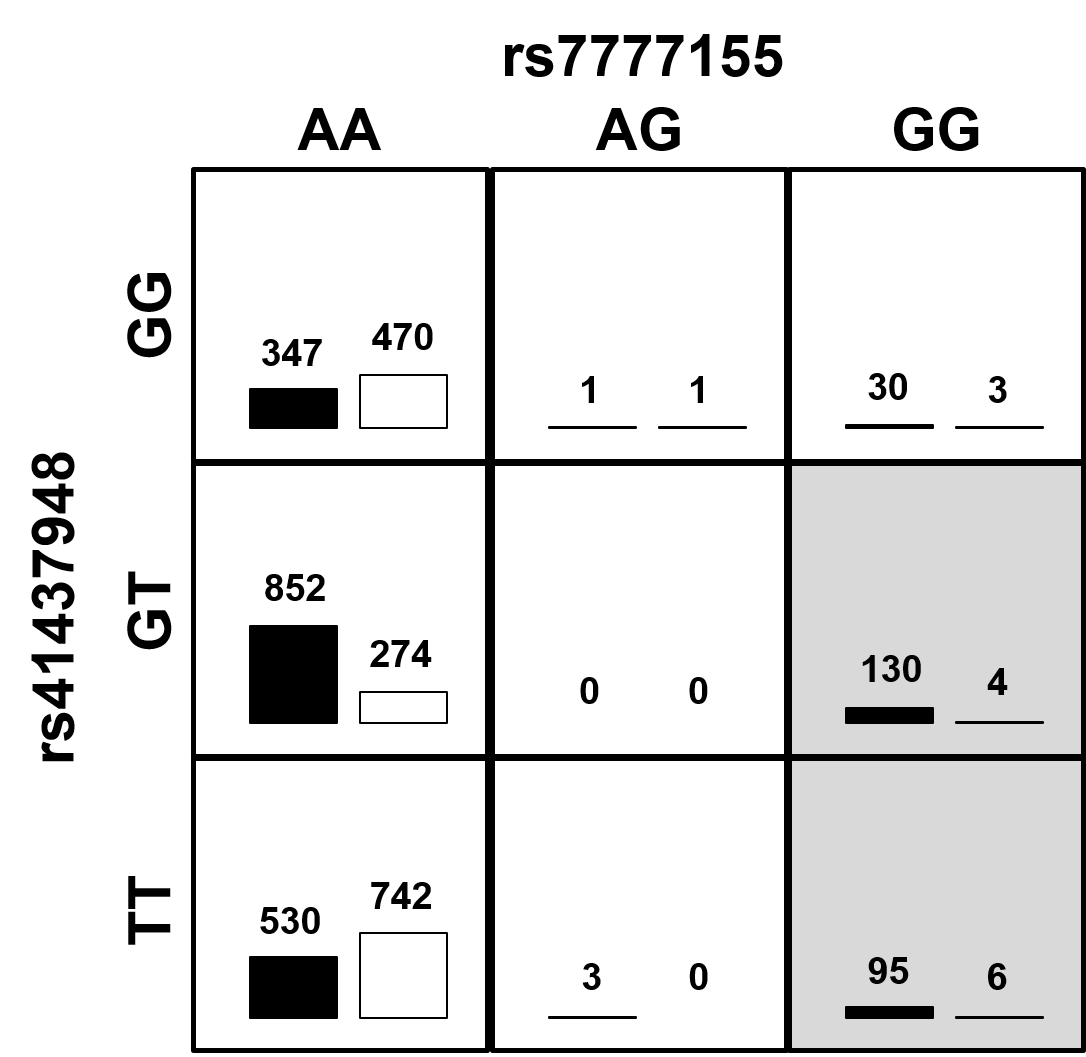 |
| --- | --- |

**Chromosome 8**

| 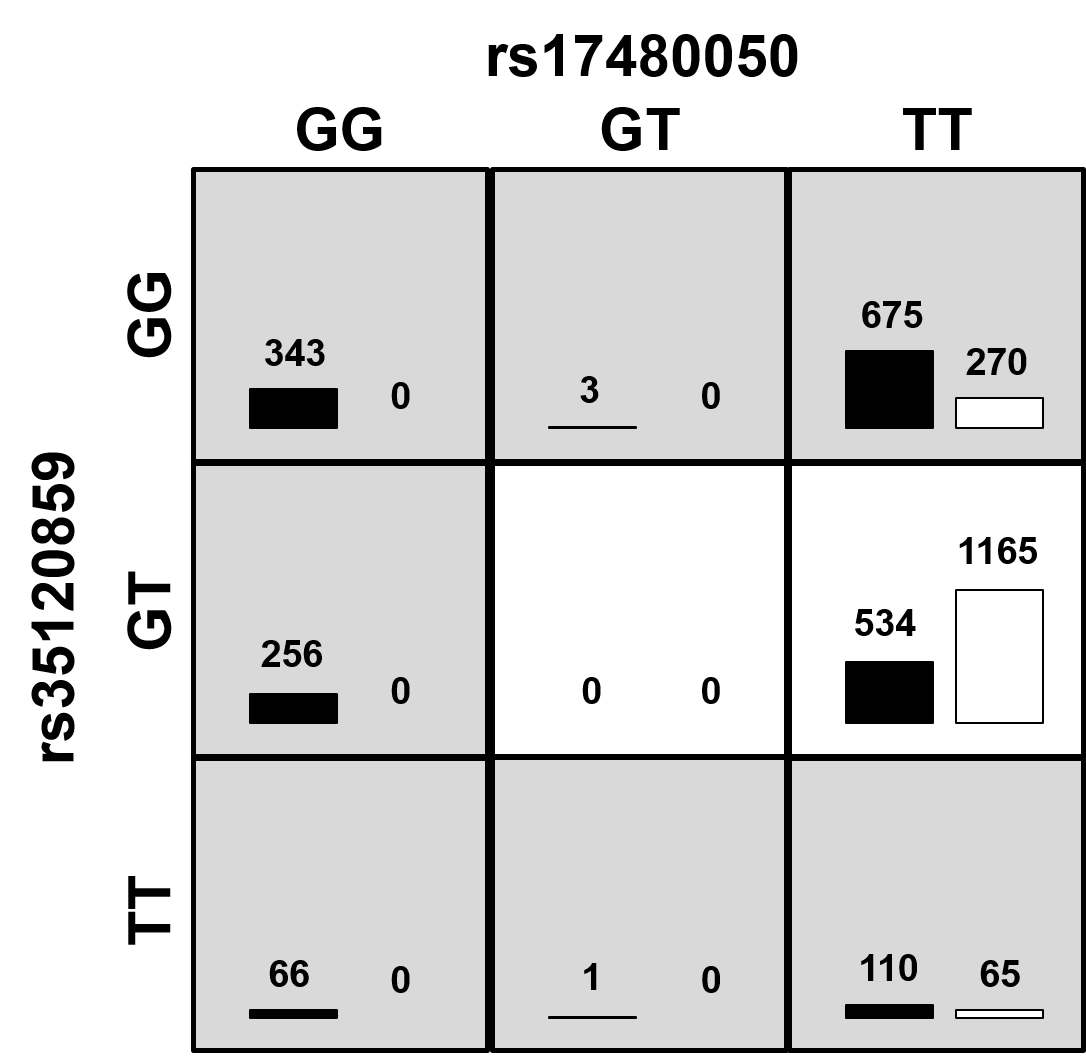 | 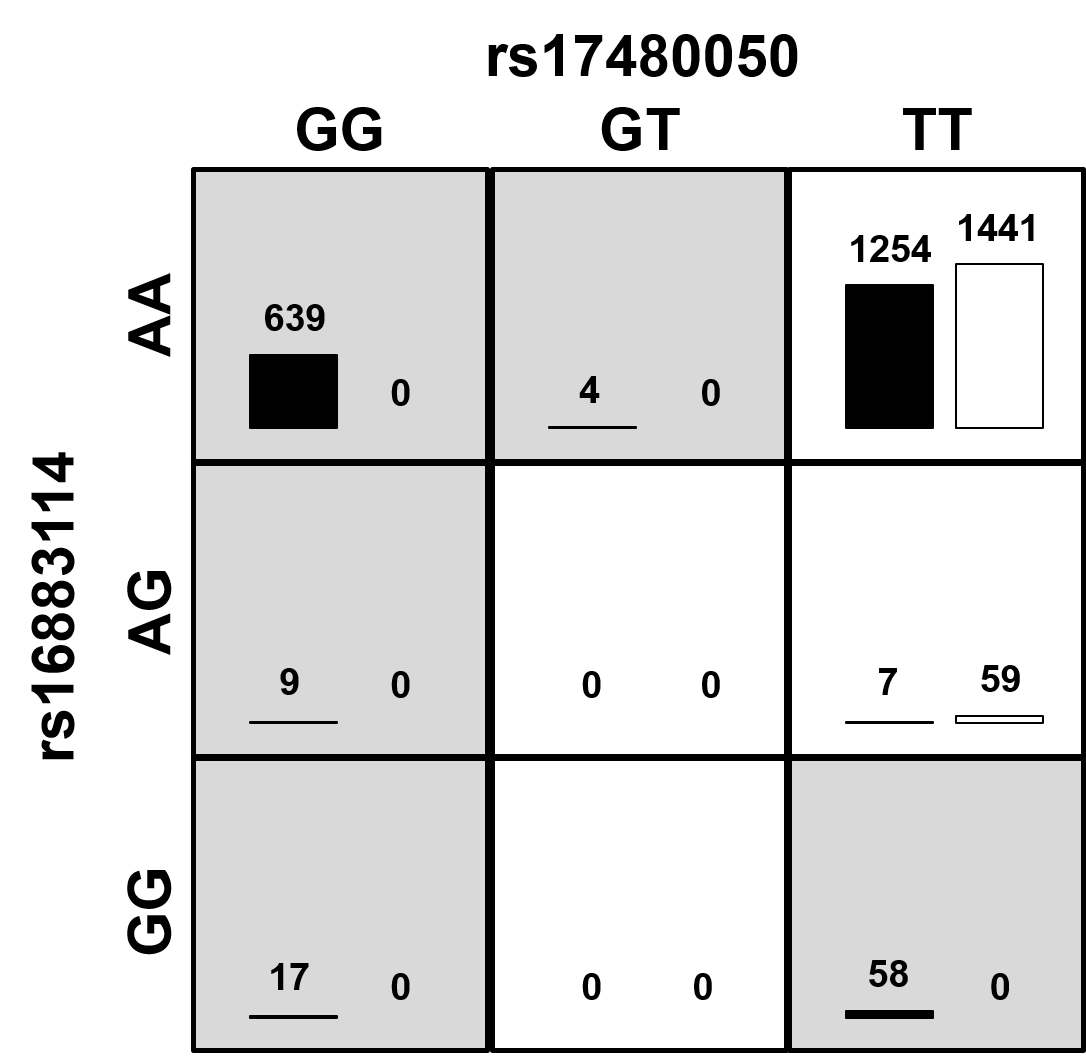 |
| --- | --- |

**Chromosome 9**


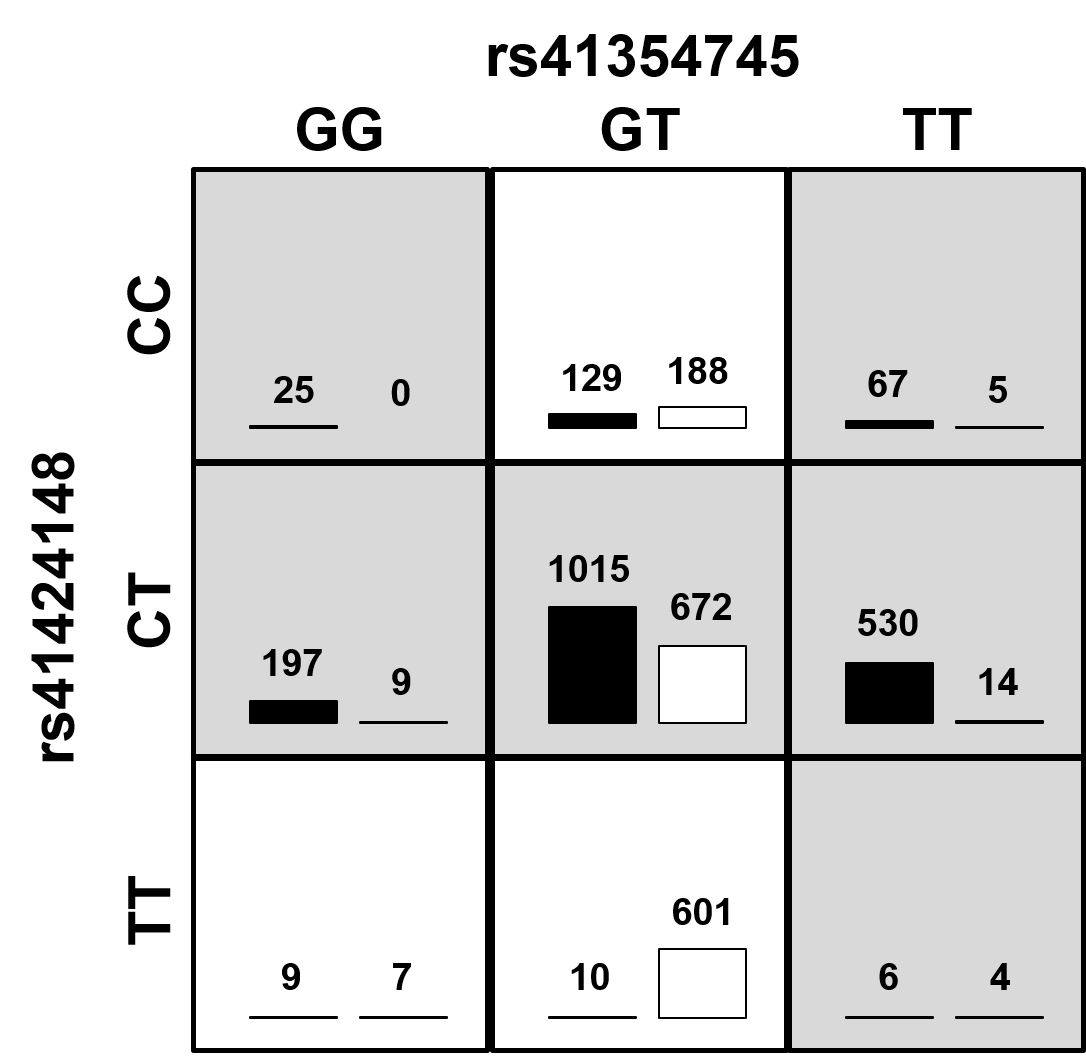


**Chromosome 10**


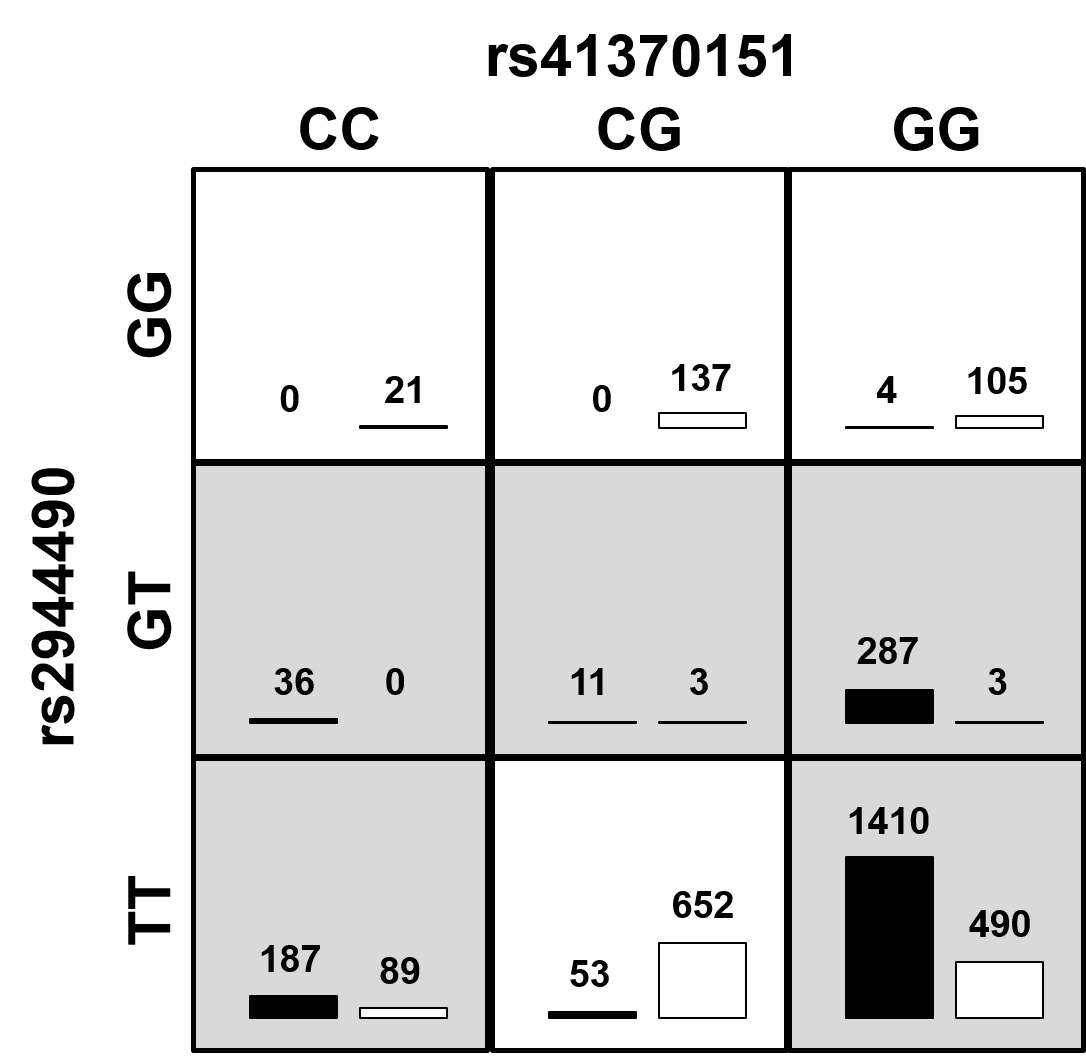


**Chromosome 11**


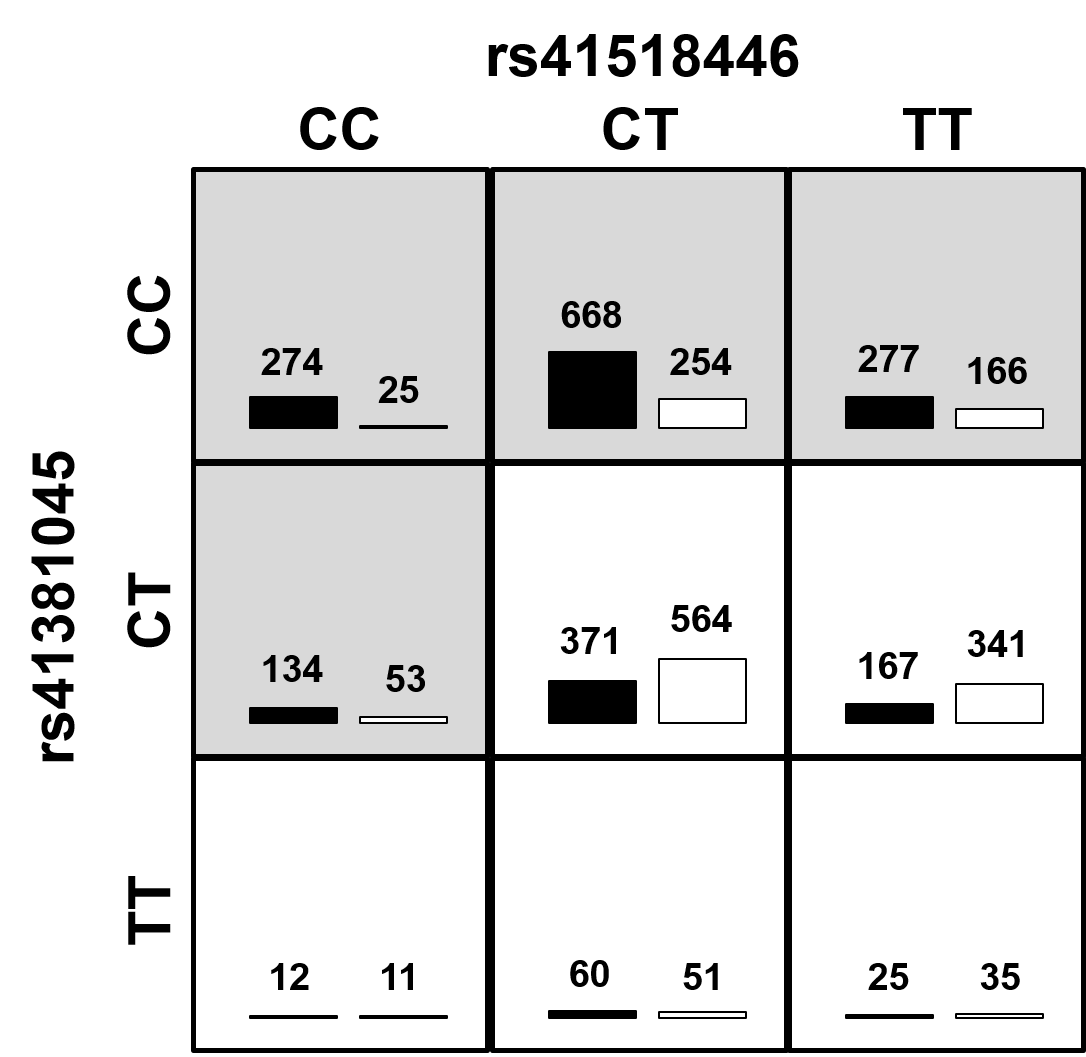


**Chromosome 12**


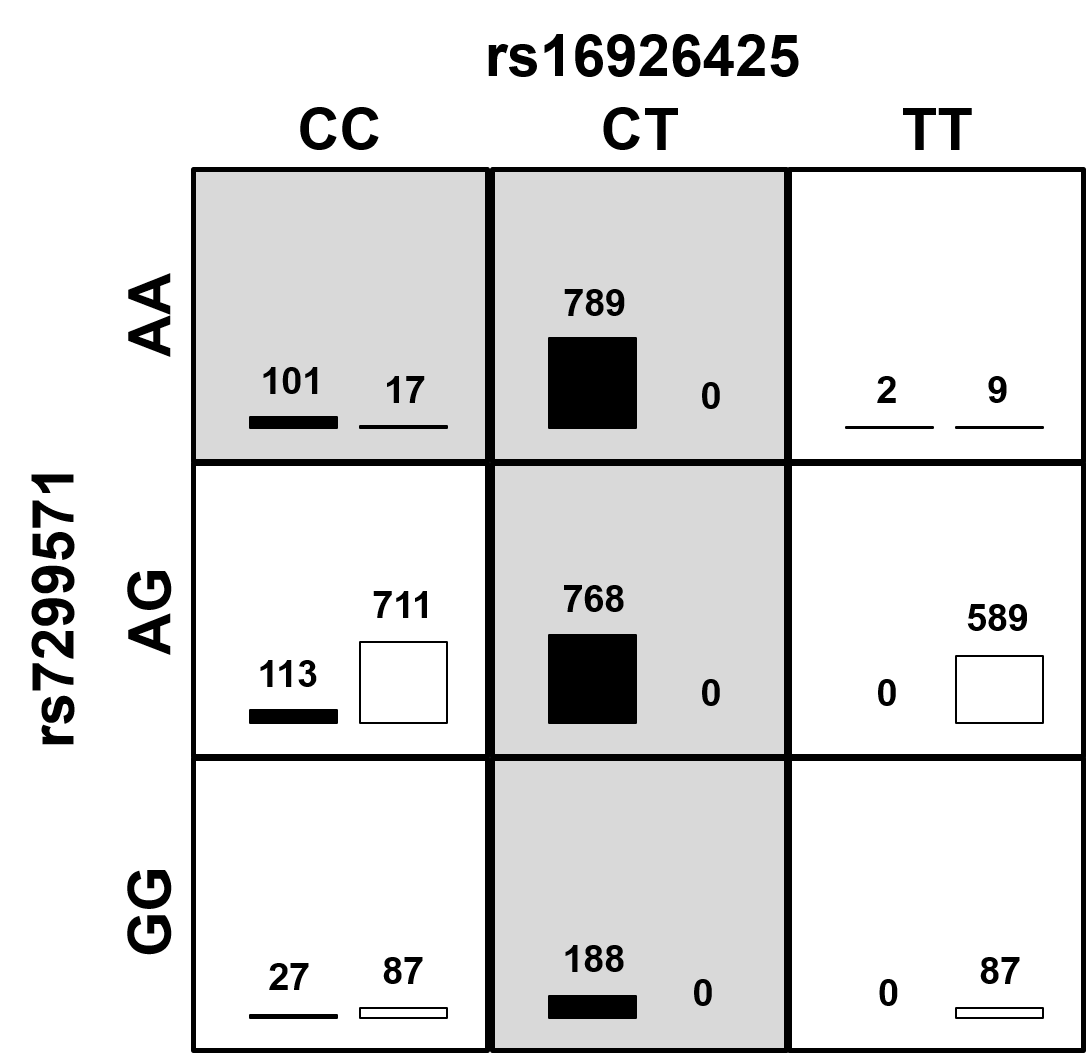


**Chromosome 13**


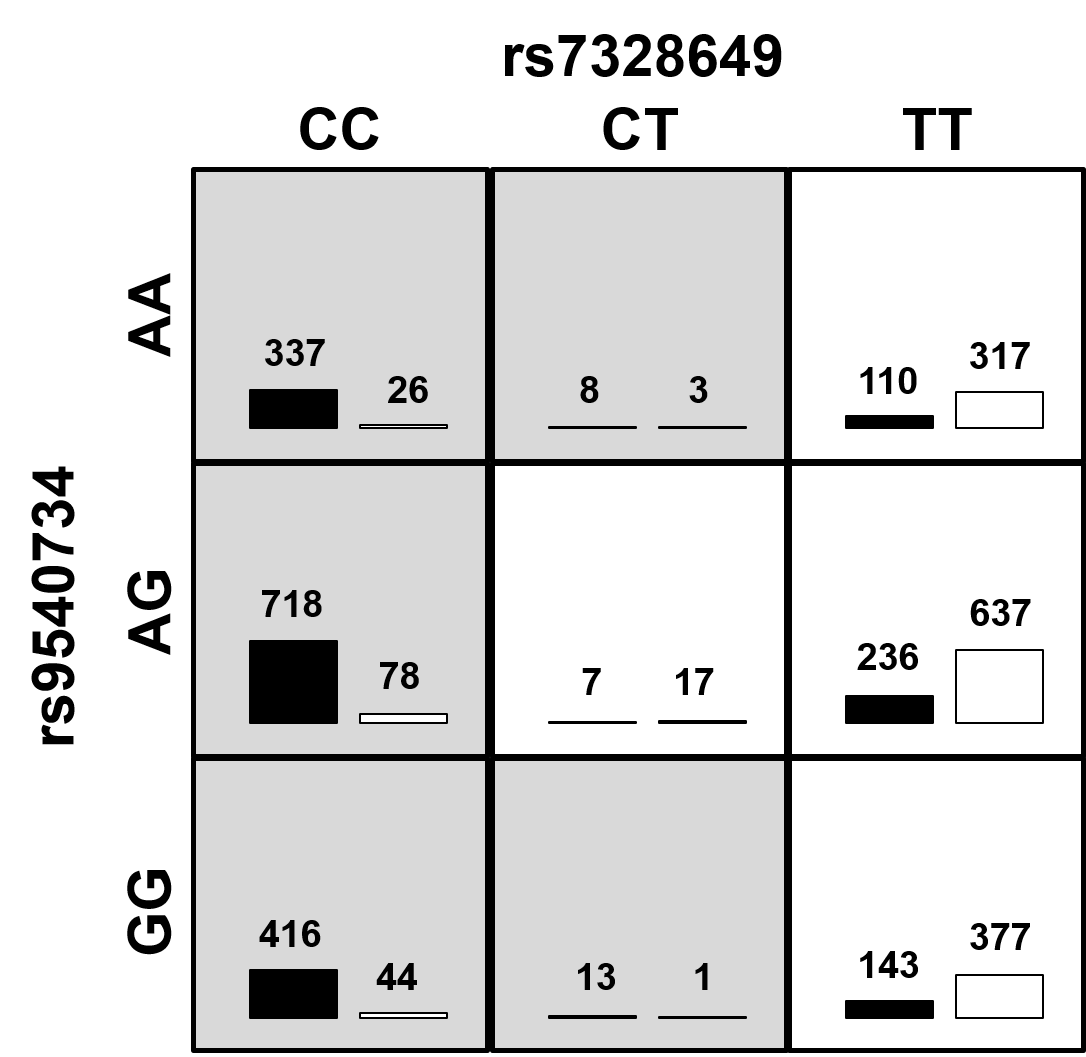


**Chromosome 14**

| 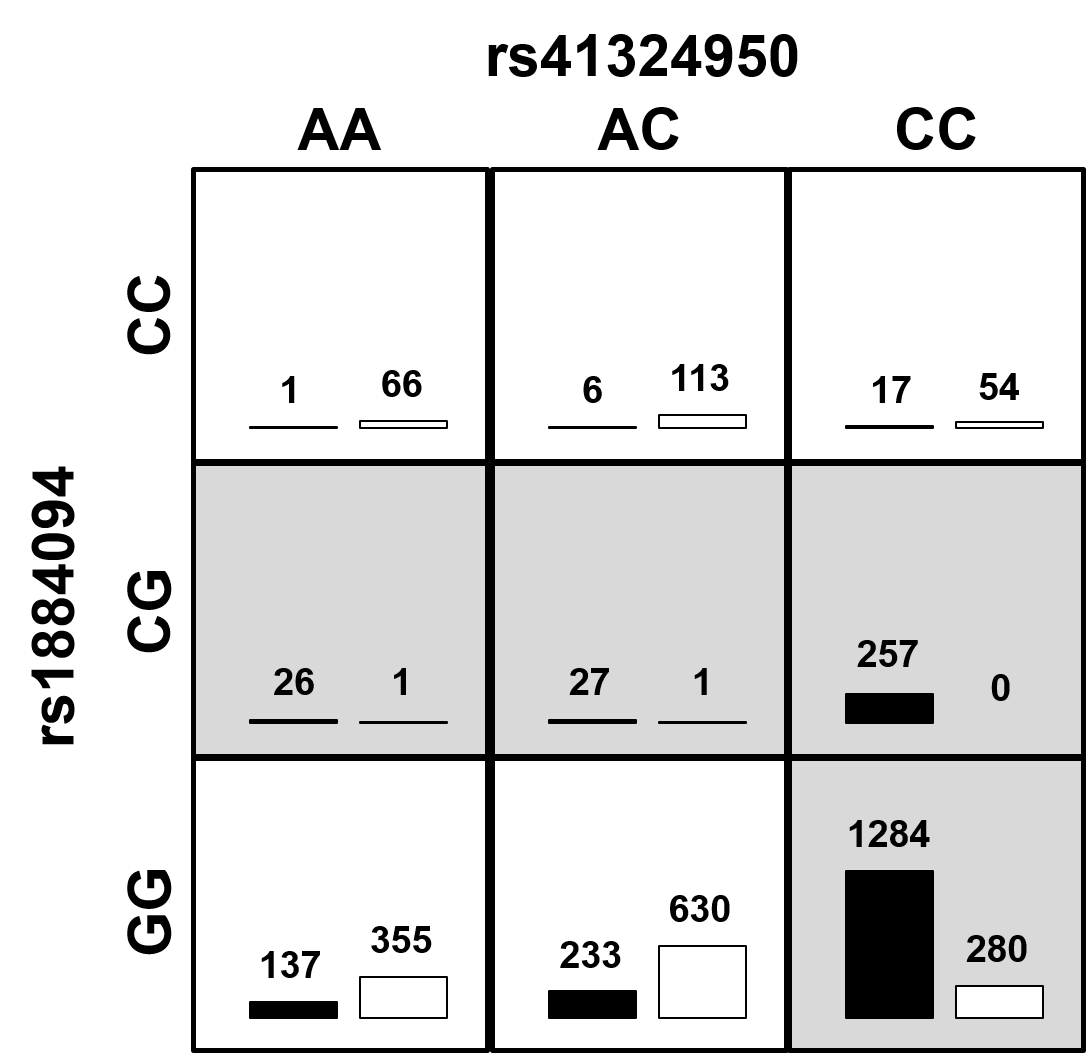 | 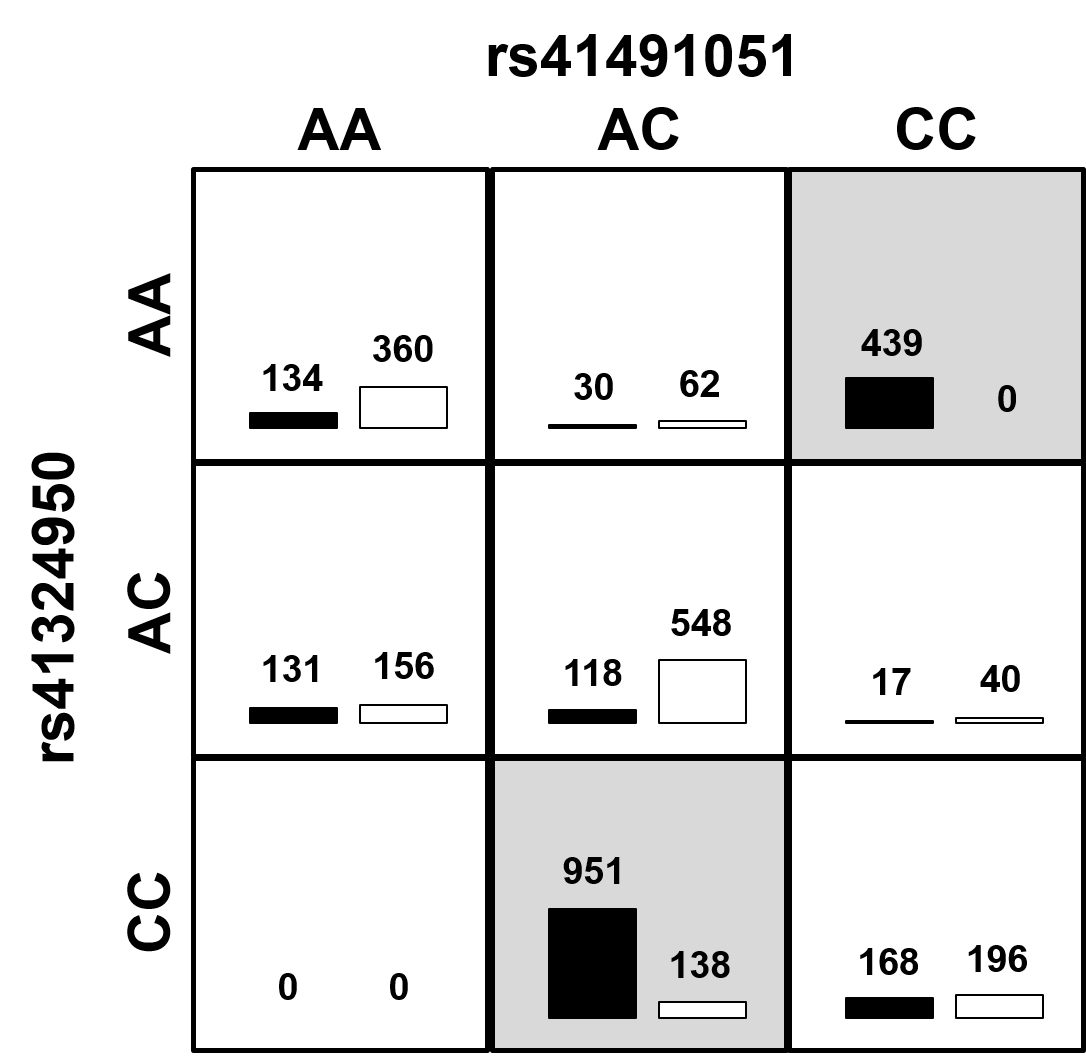 |
| --- | --- |

**Chromosome** 15

| 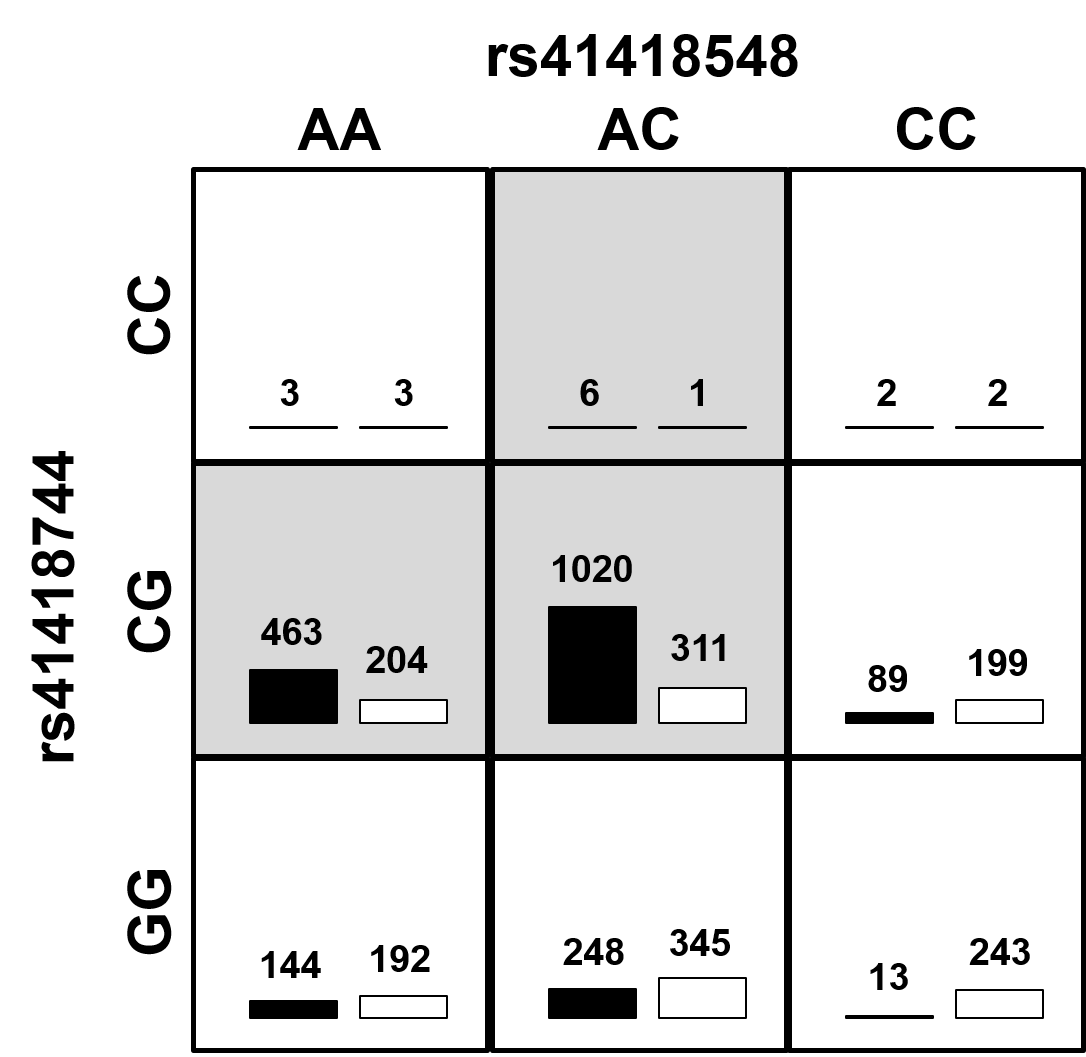 | 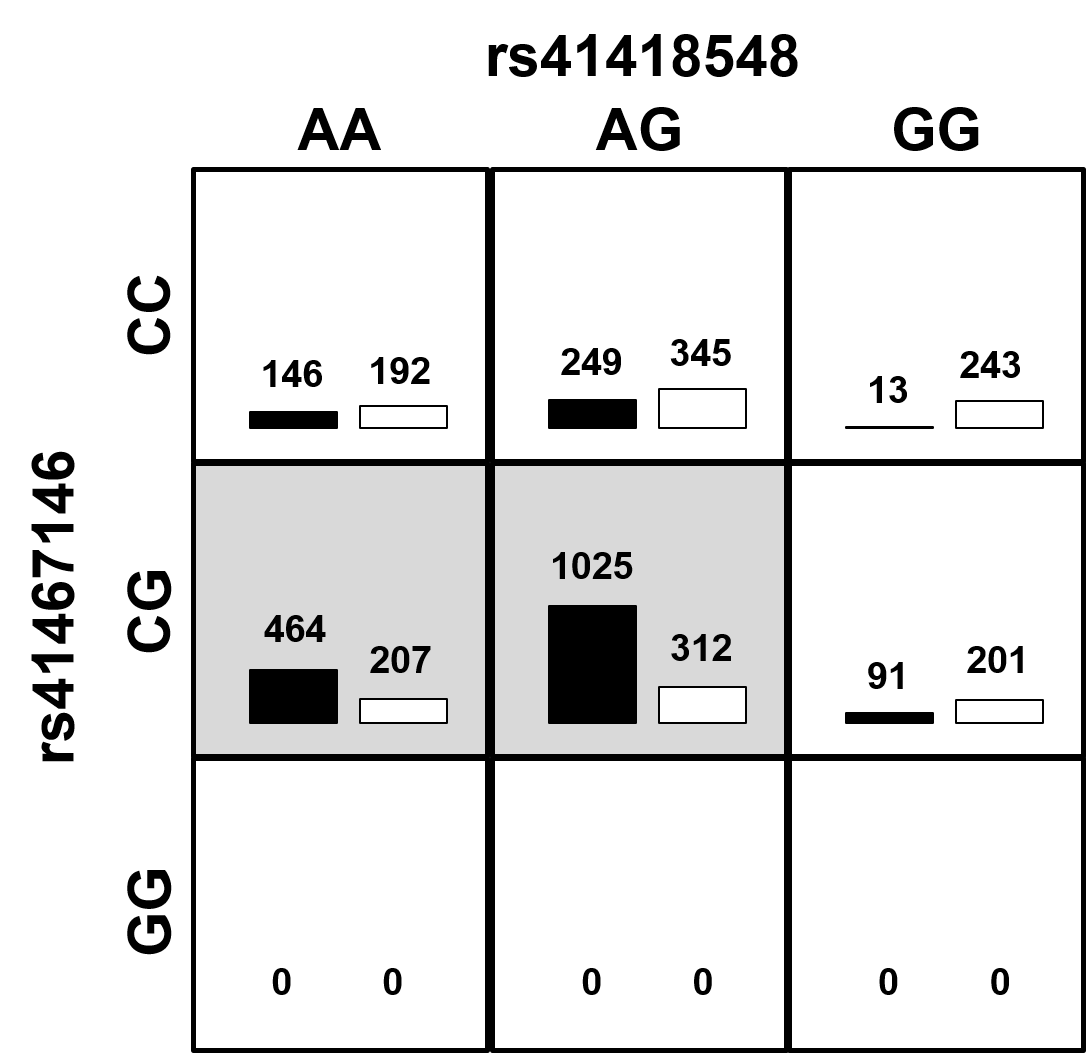 |
| --- | --- |

**Chromosome 16**


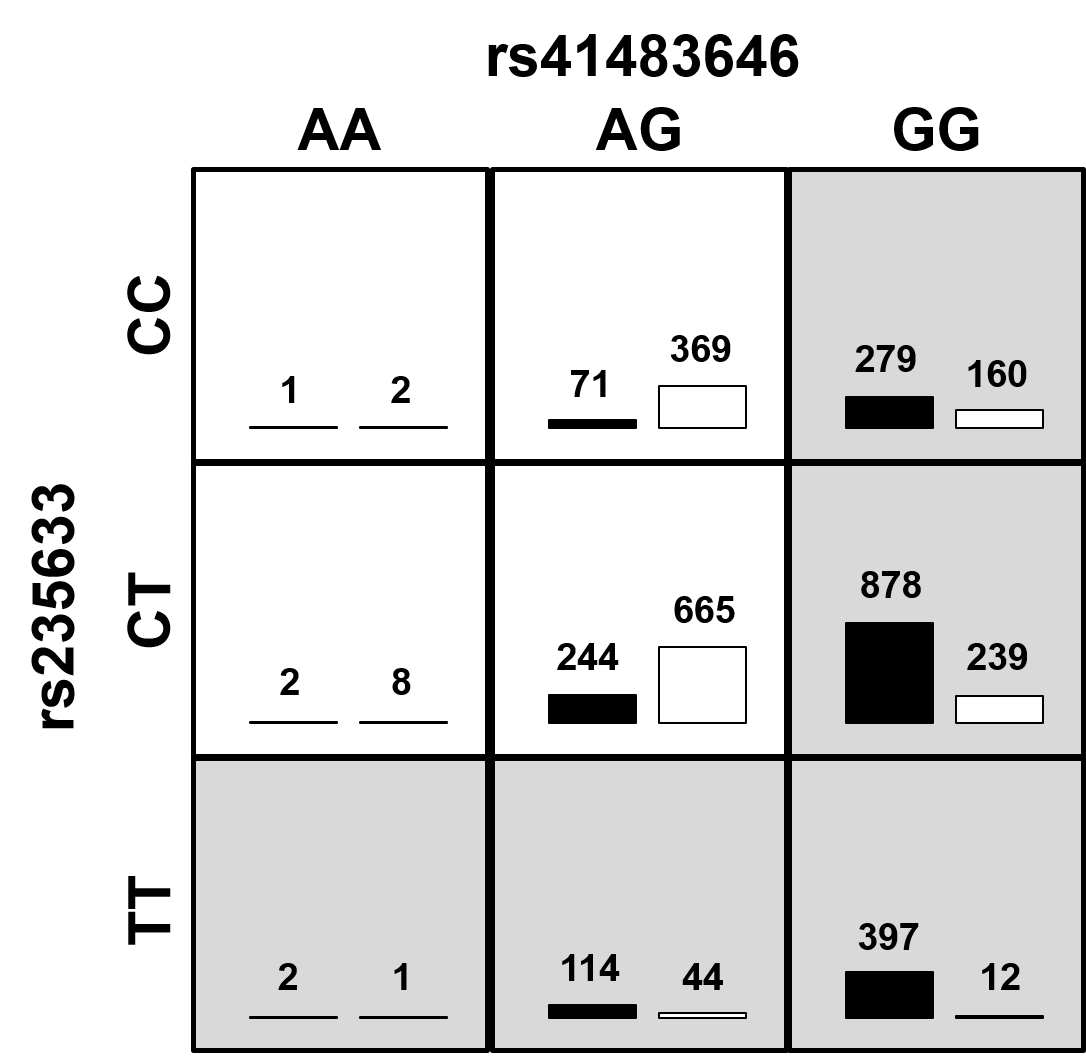


**Chromosome** 17

| 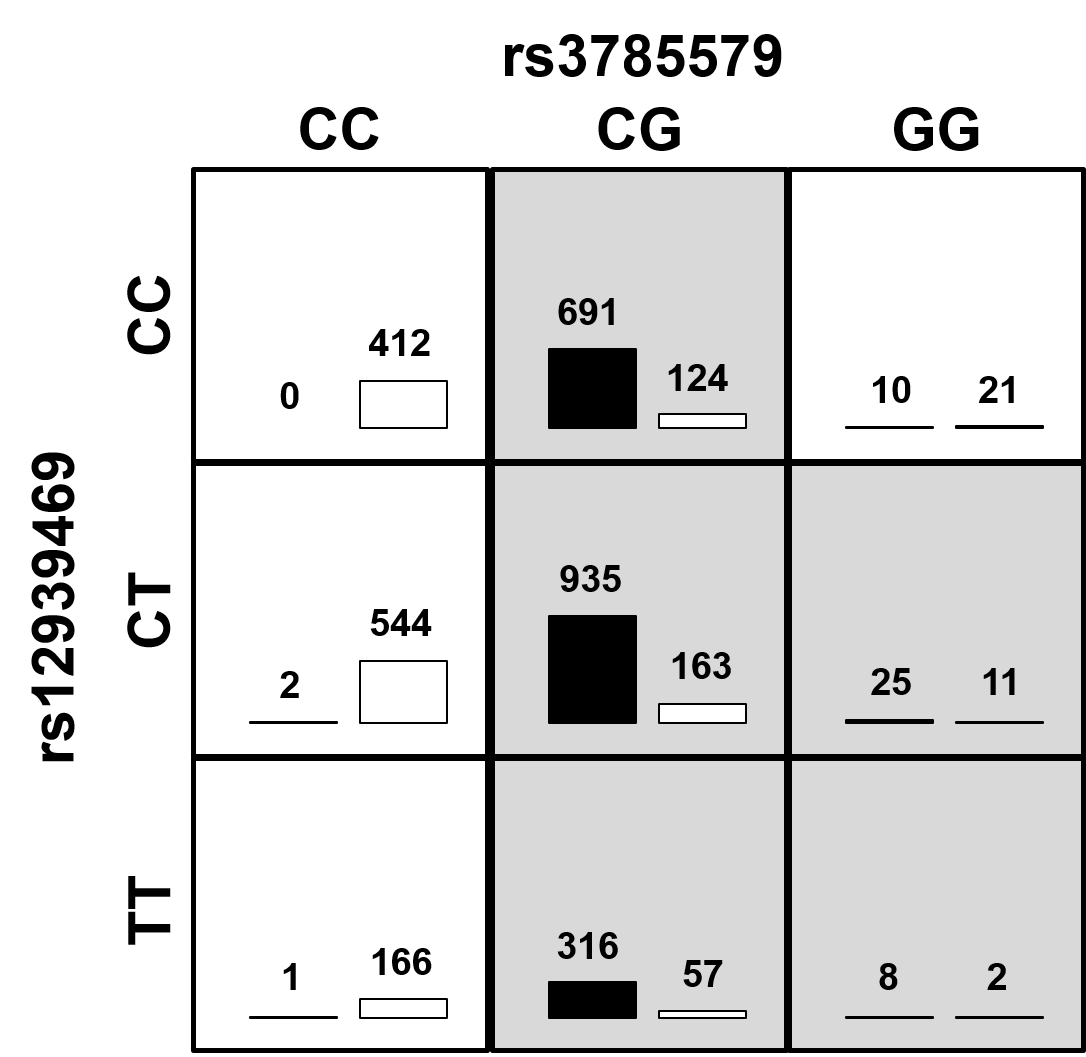 | 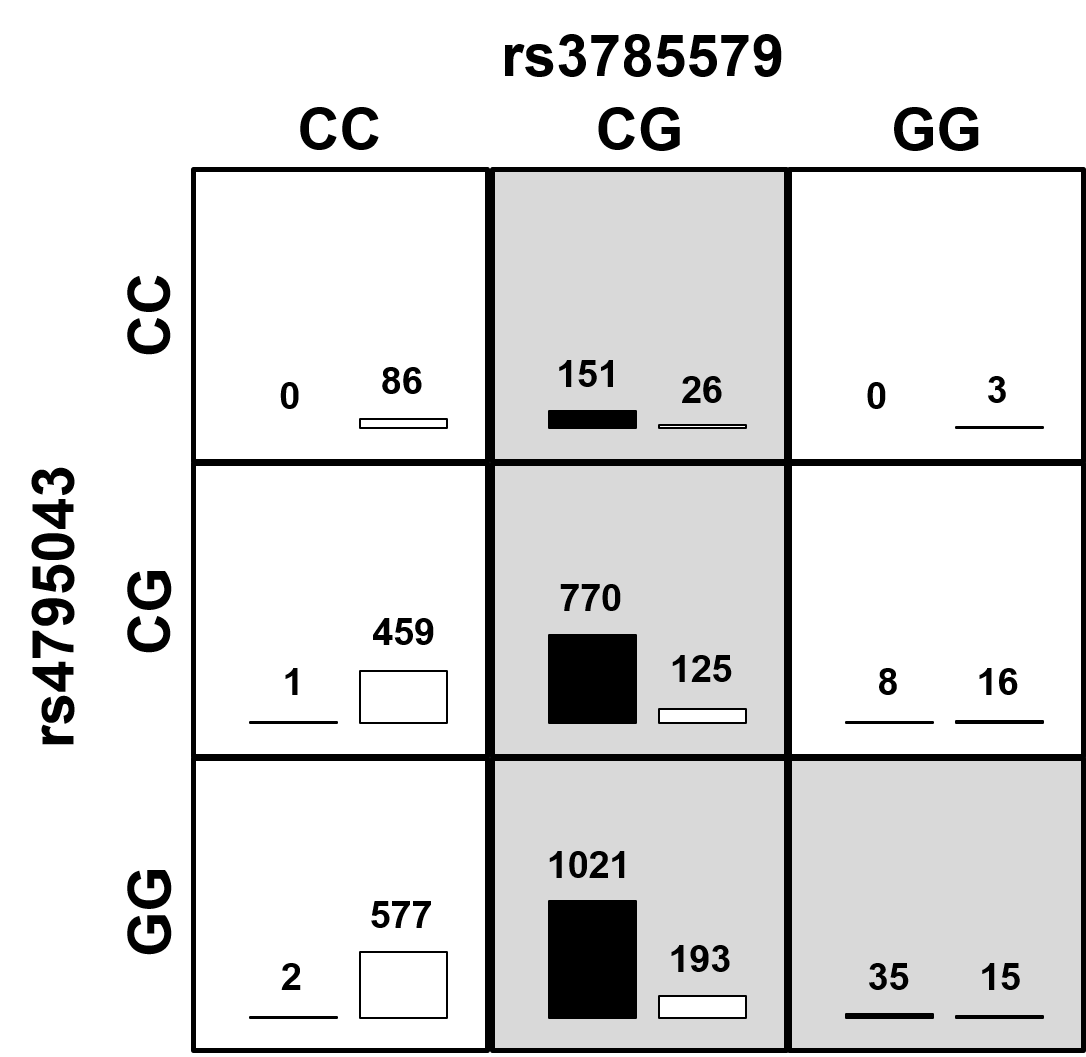 |
| --- | --- |
| 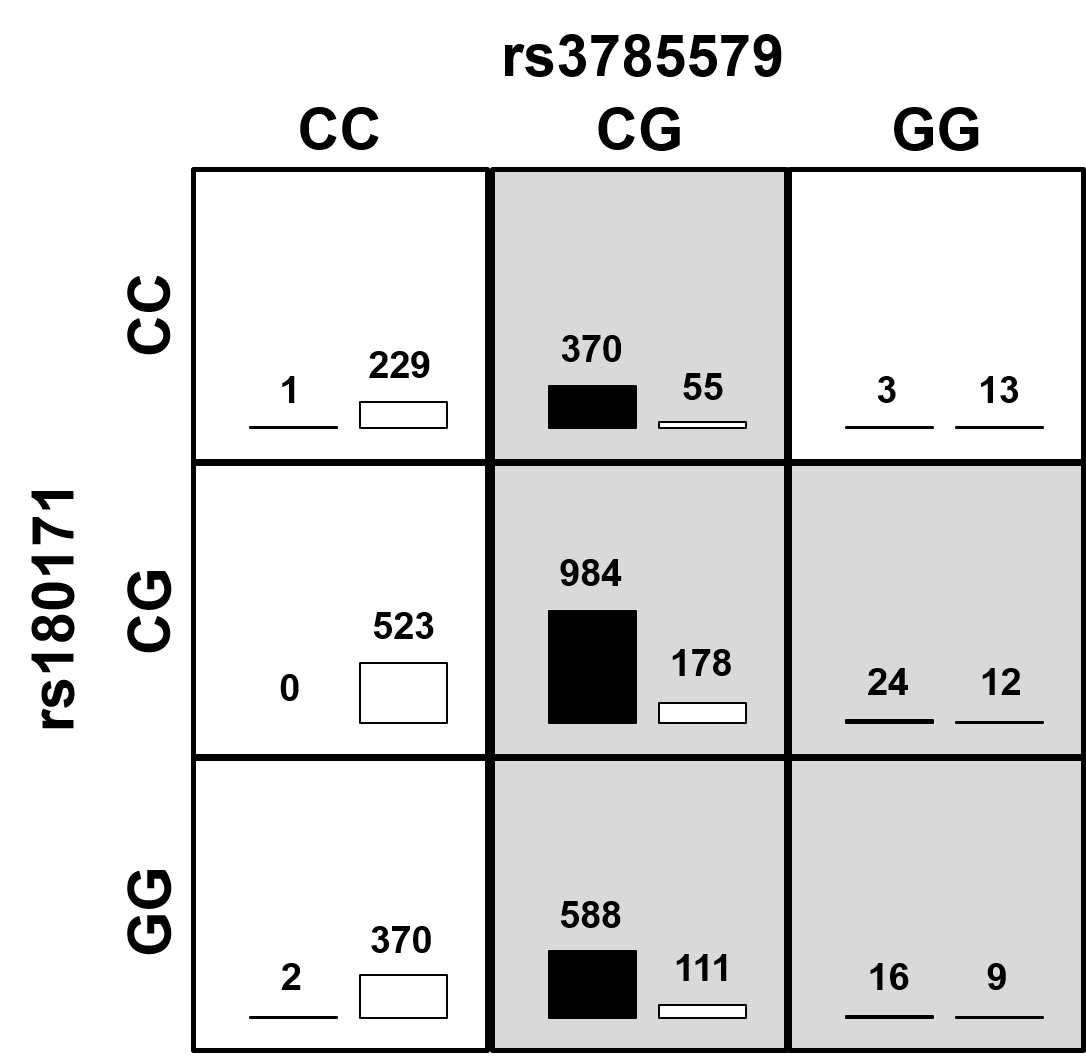 | 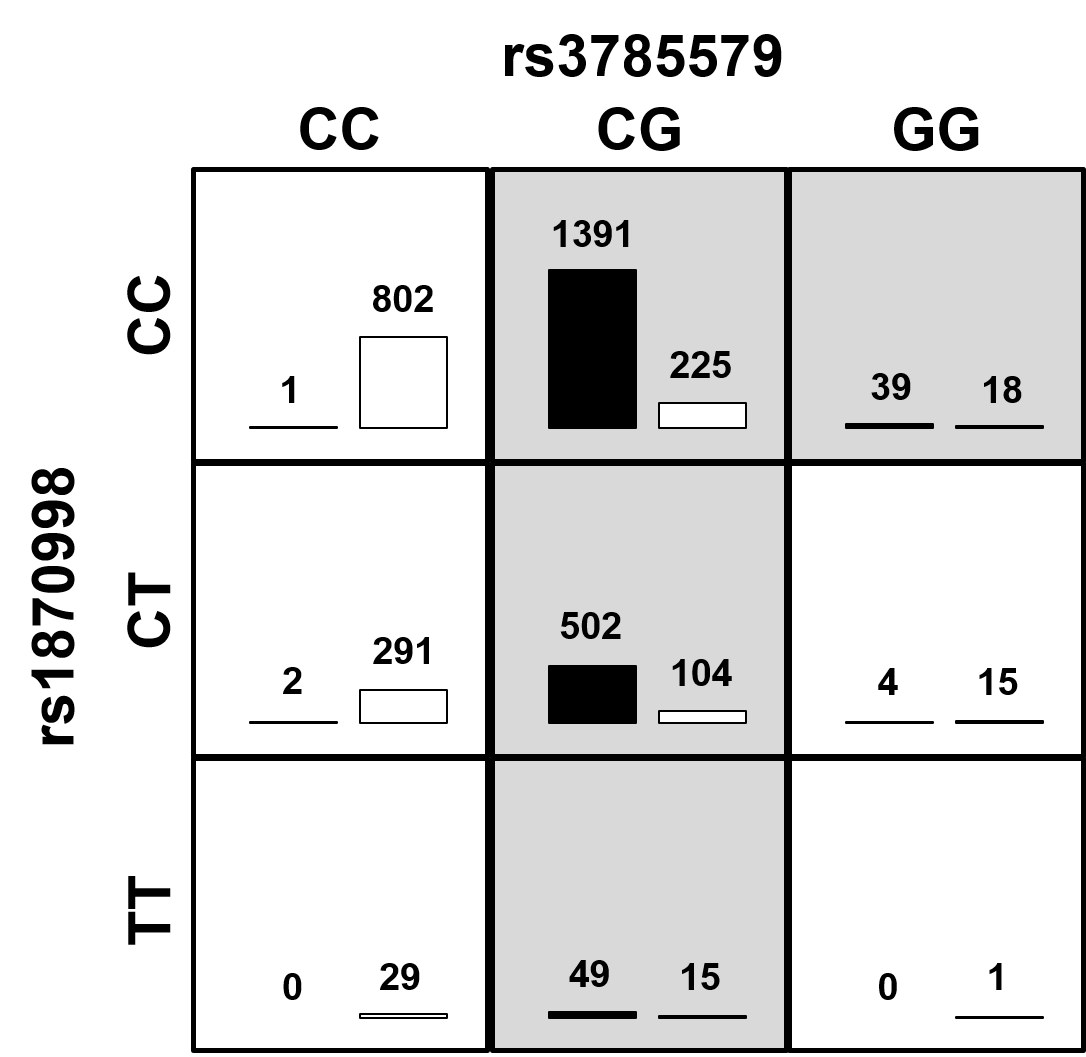 |
| 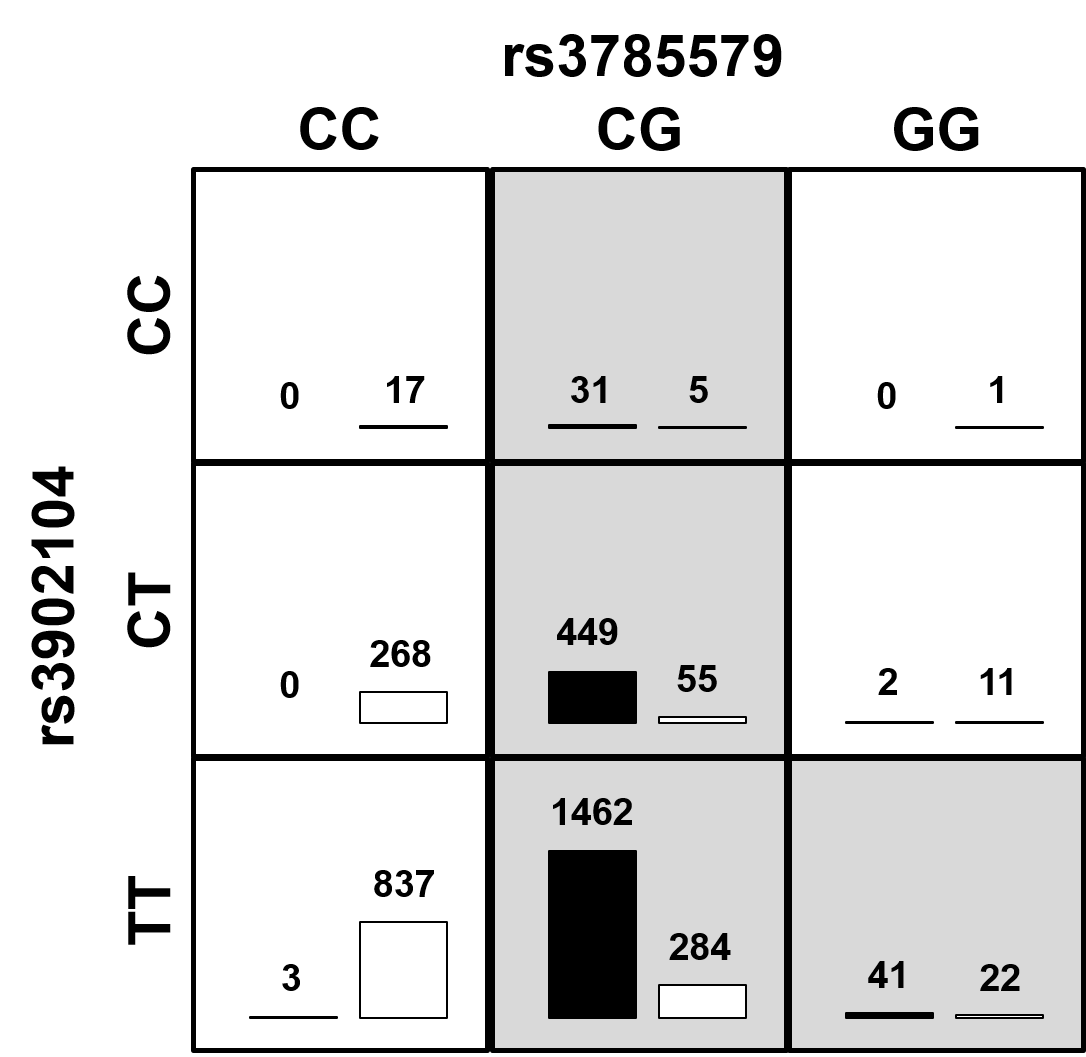 | |

**Chromosome** 18

| 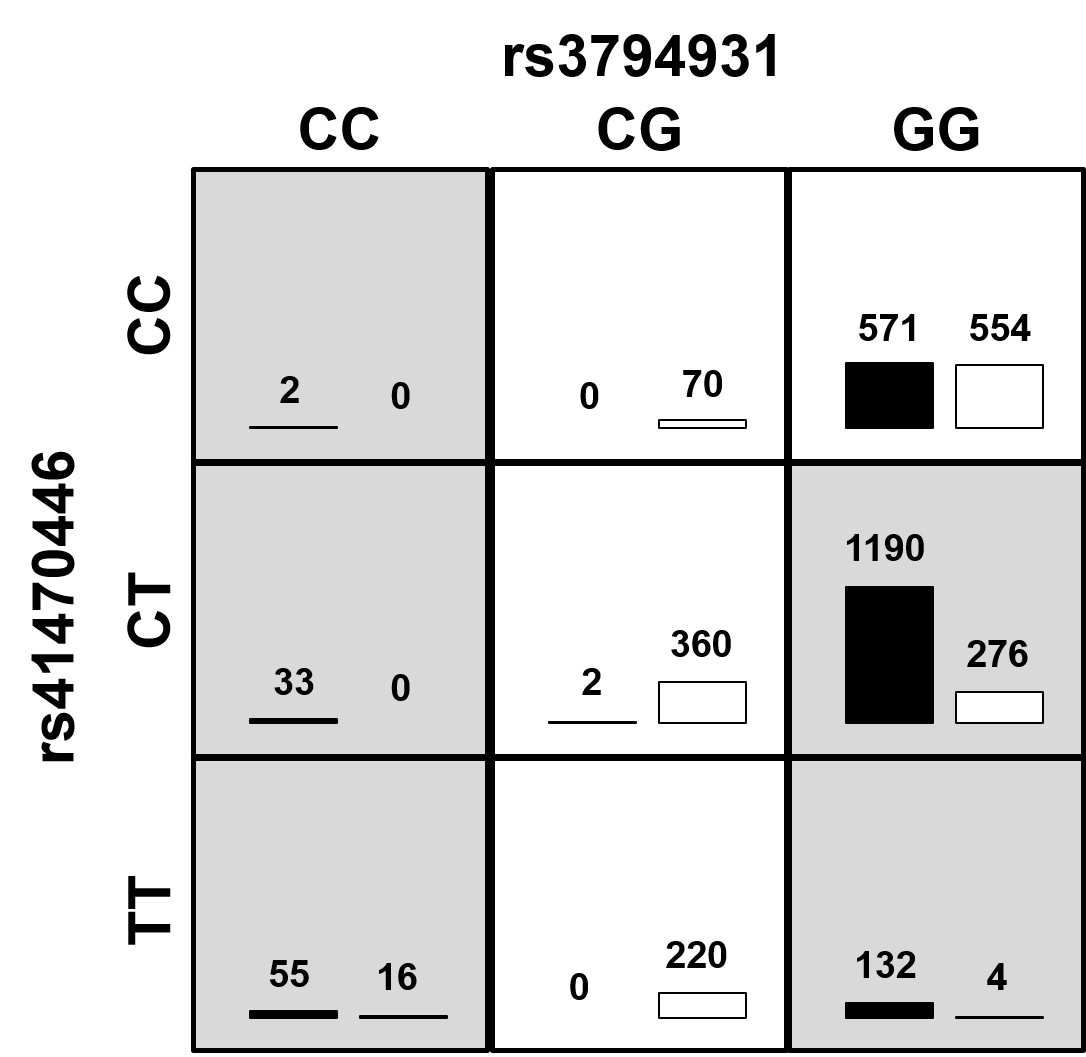 | 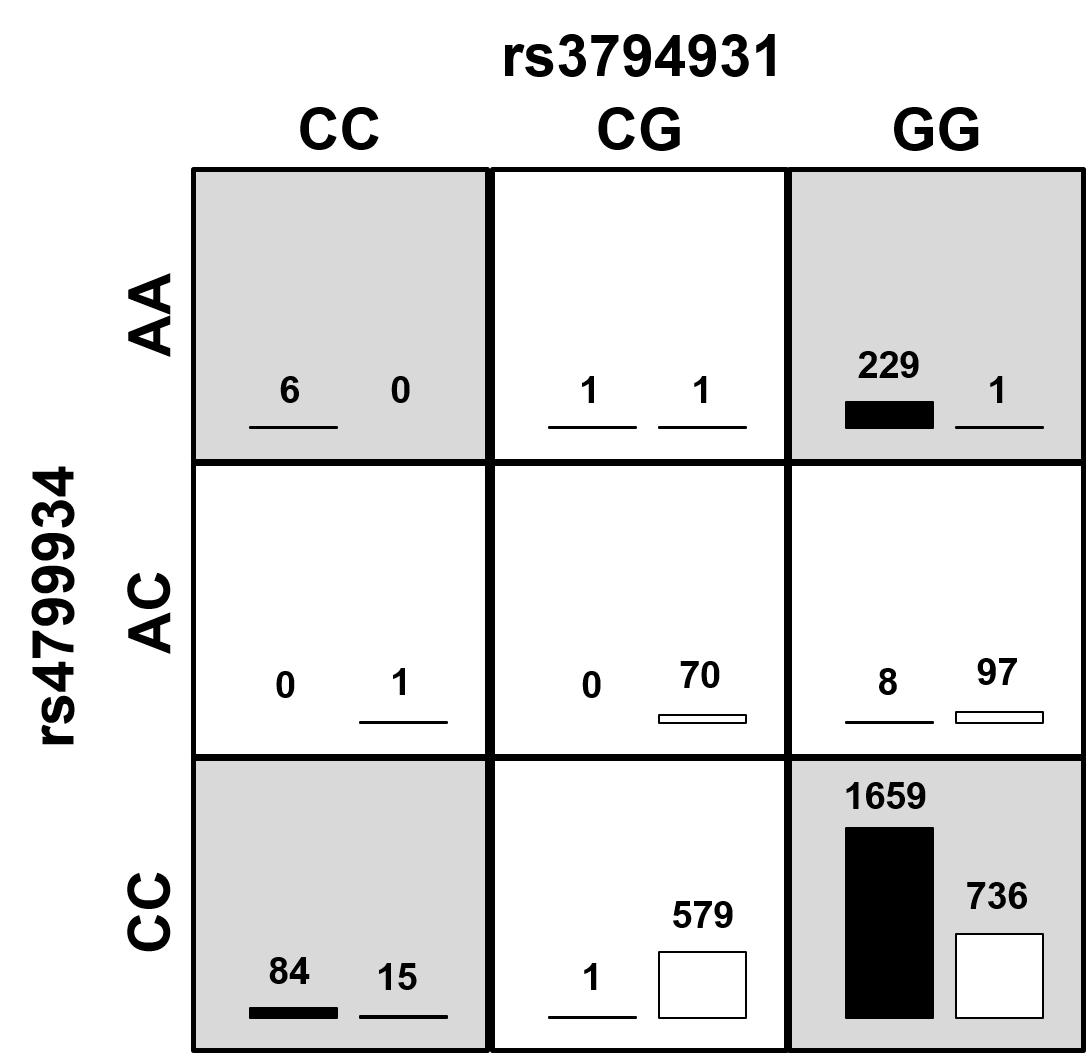 |
| --- | --- |

**Chromosome 19**

| 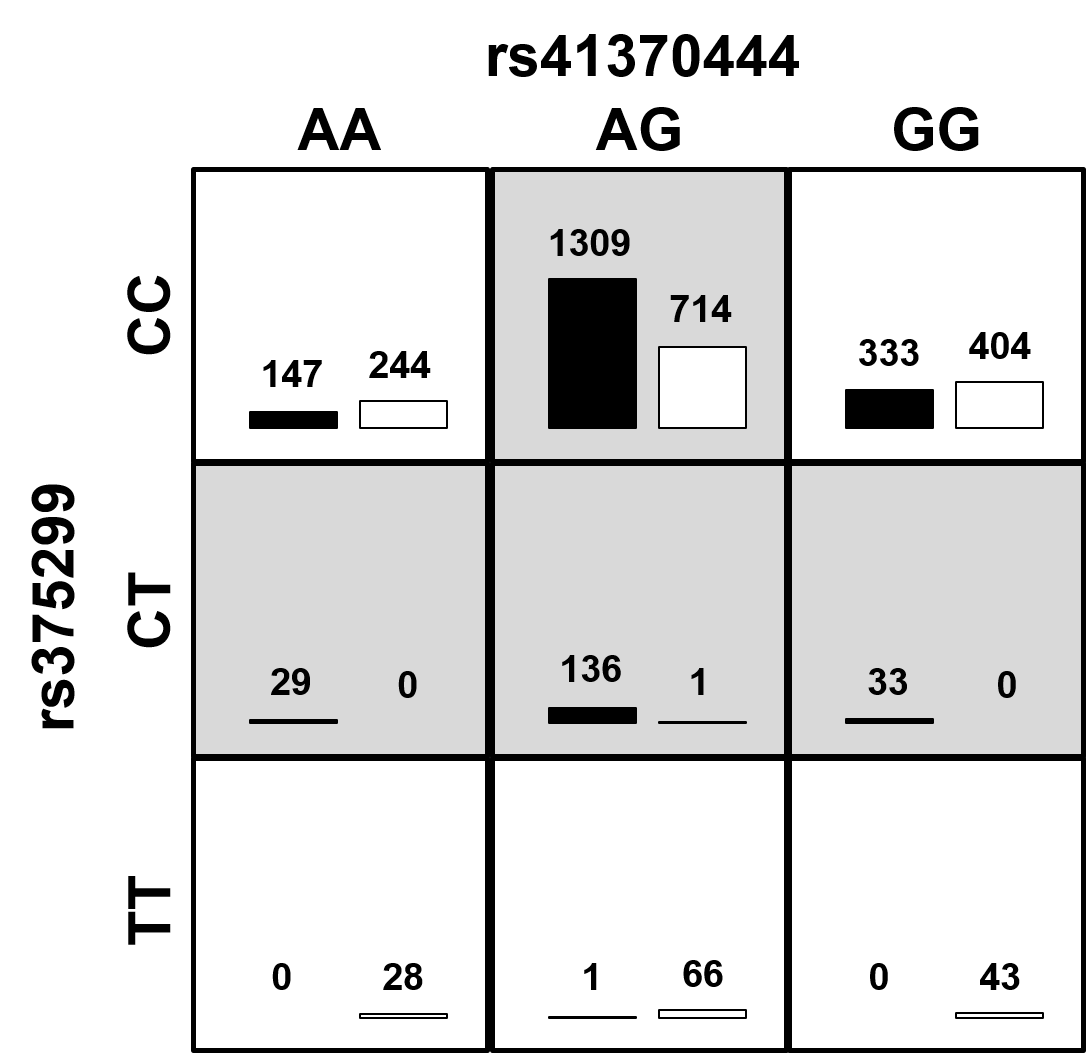 | 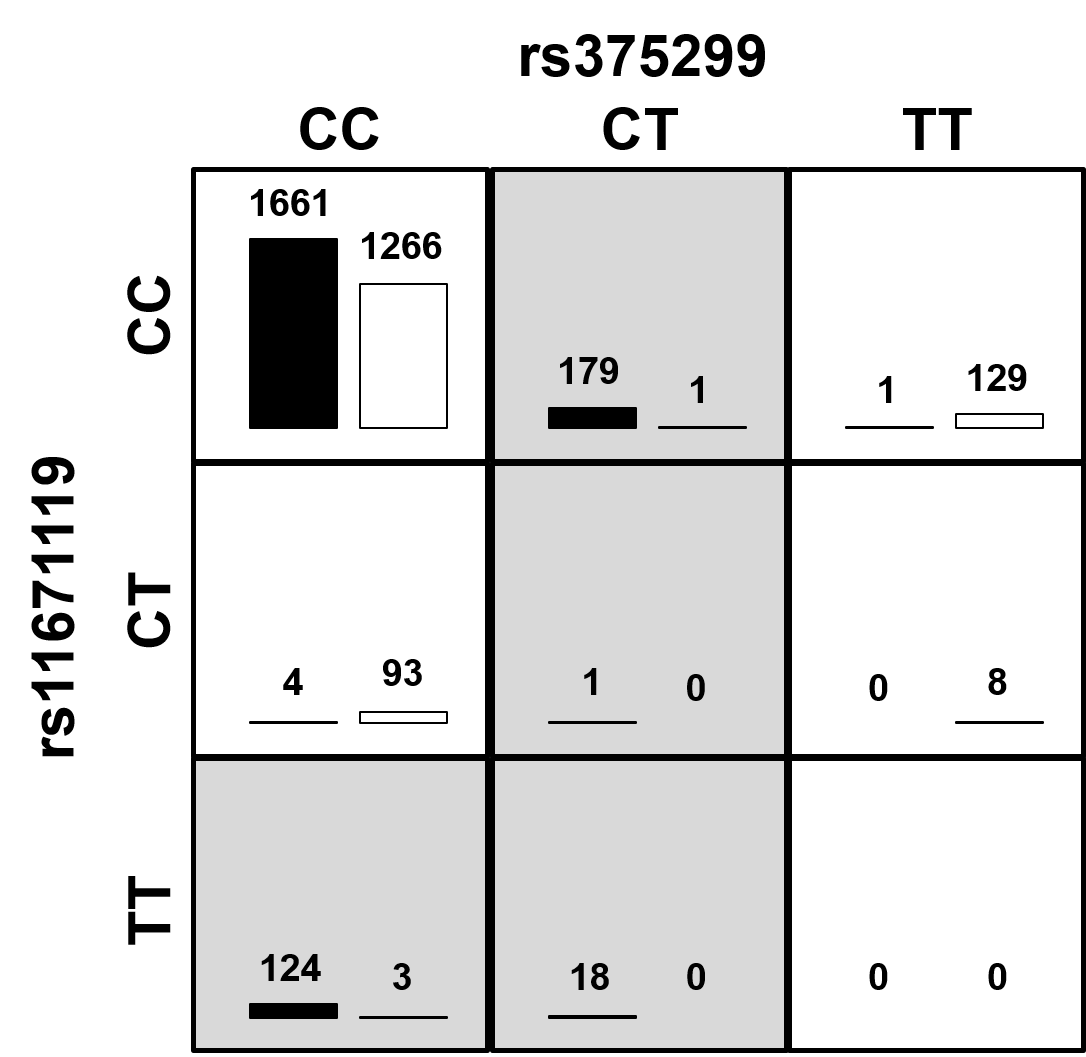 |
| --- | --- |

**Chromosome** 20

| 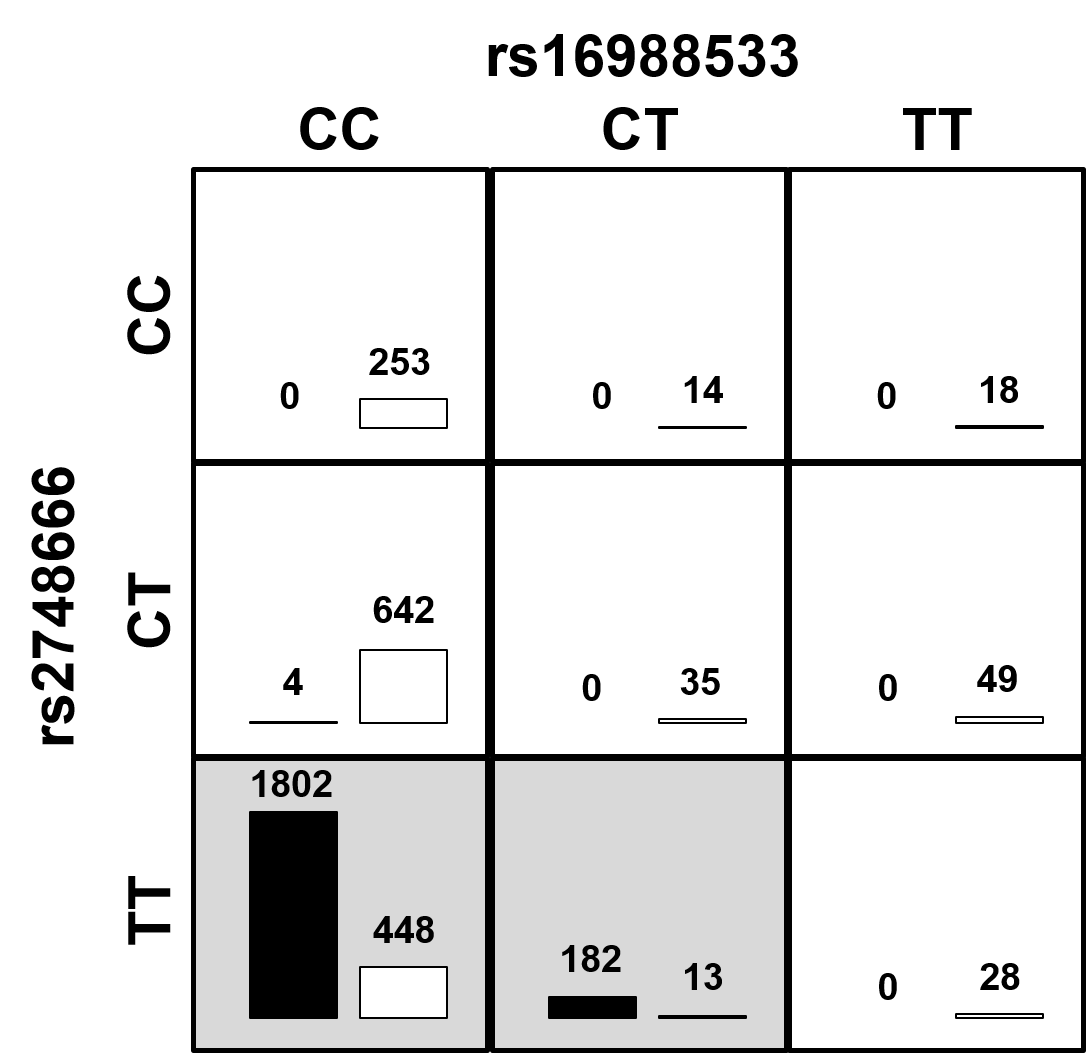 | 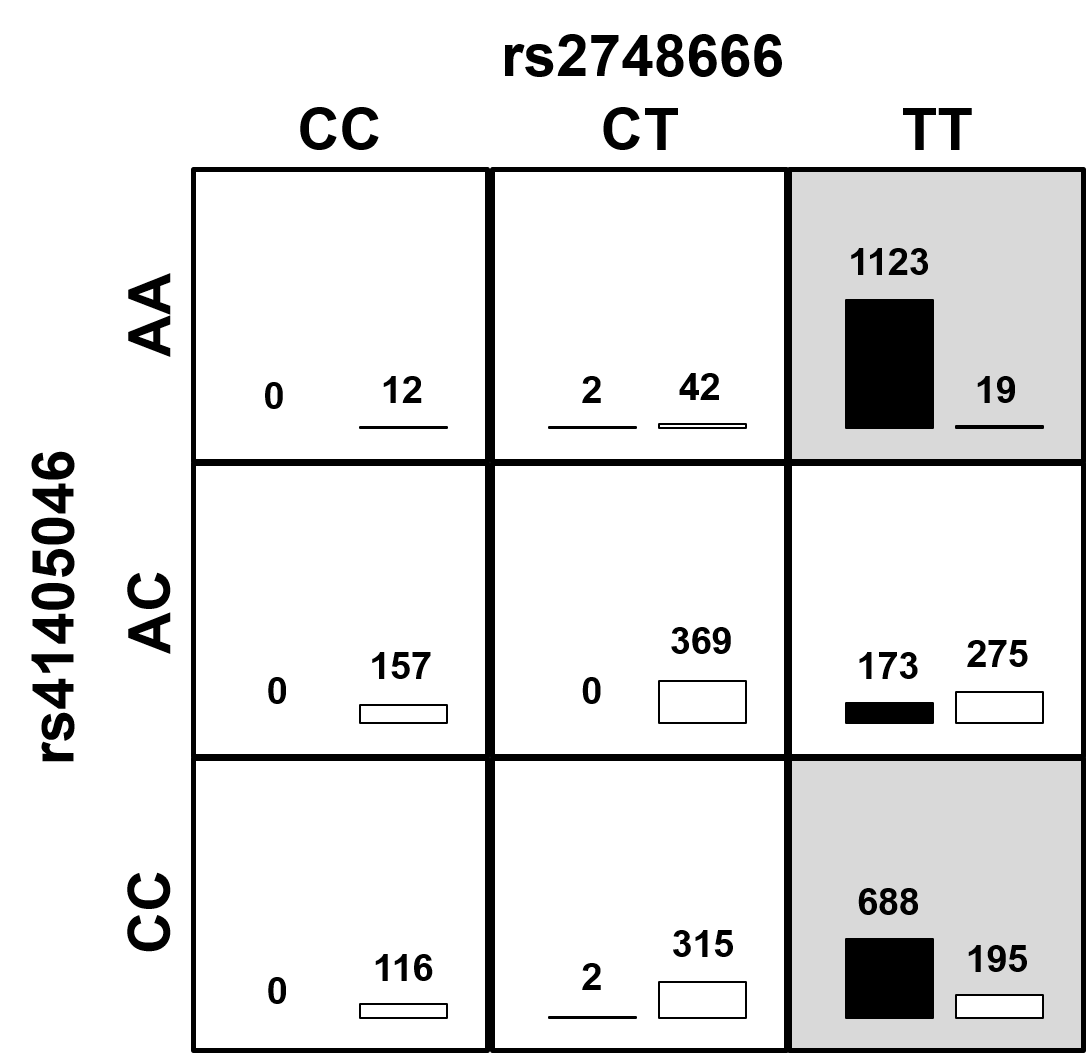 |
| --- | --- |

**Chromosome 21**

| 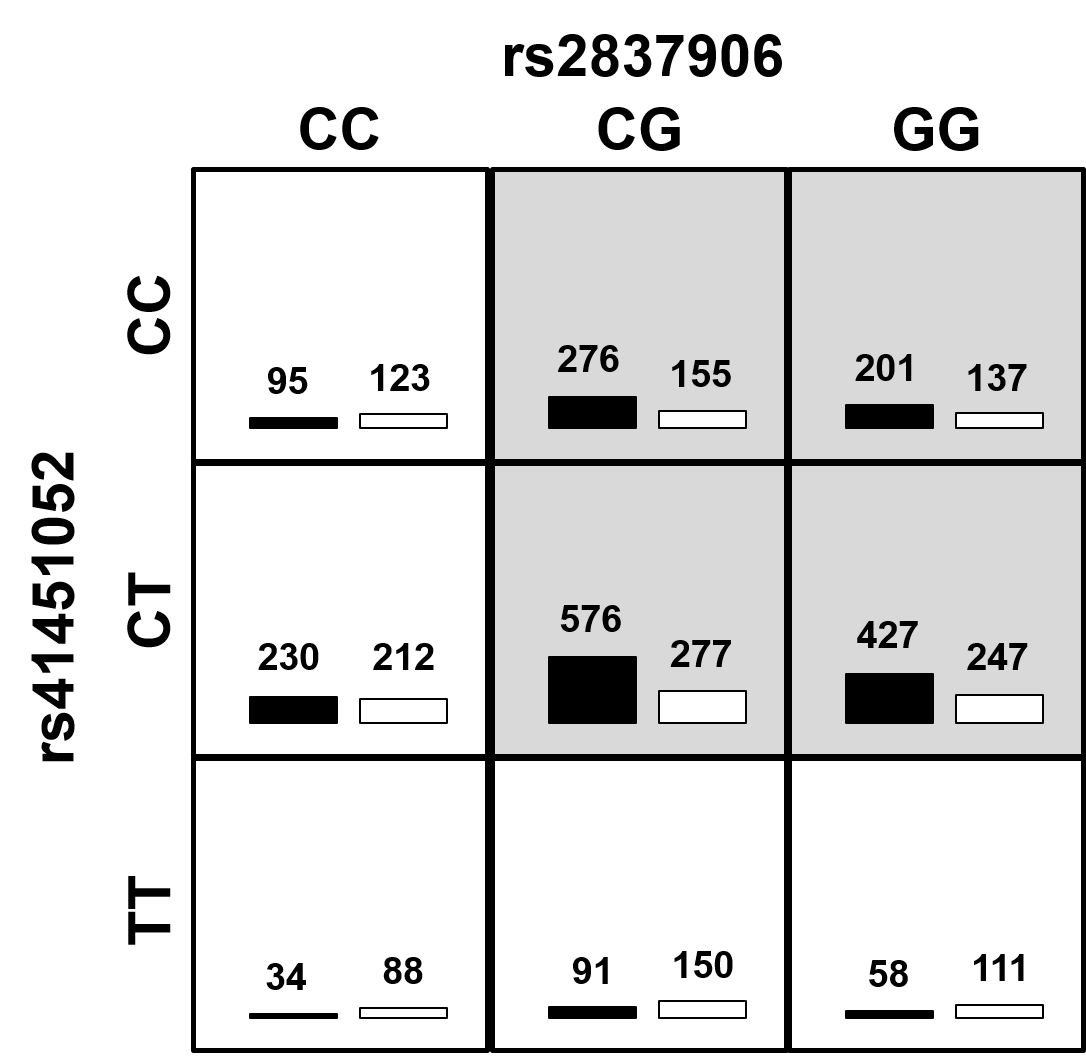 | 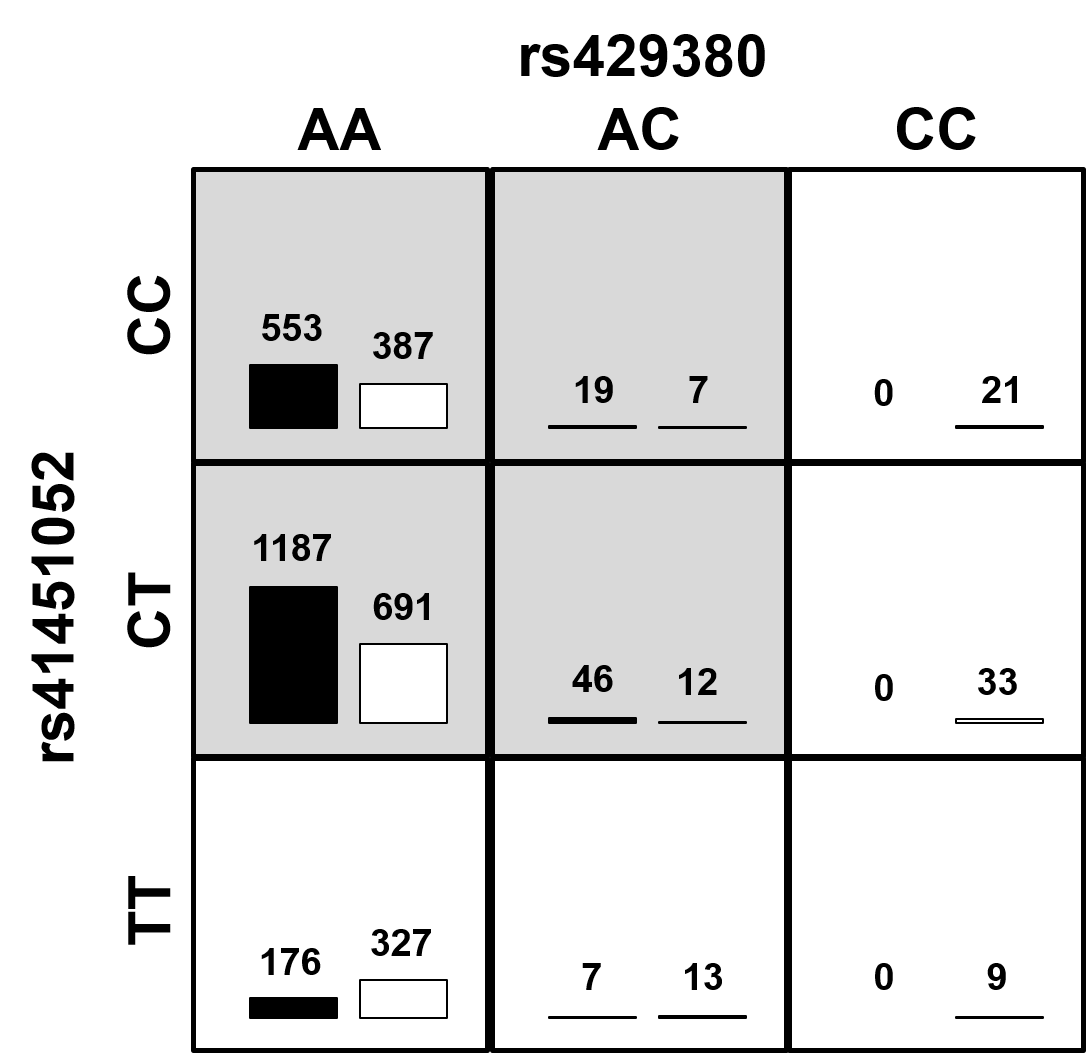 |
| --- | --- |

**Chromosome** 22

| 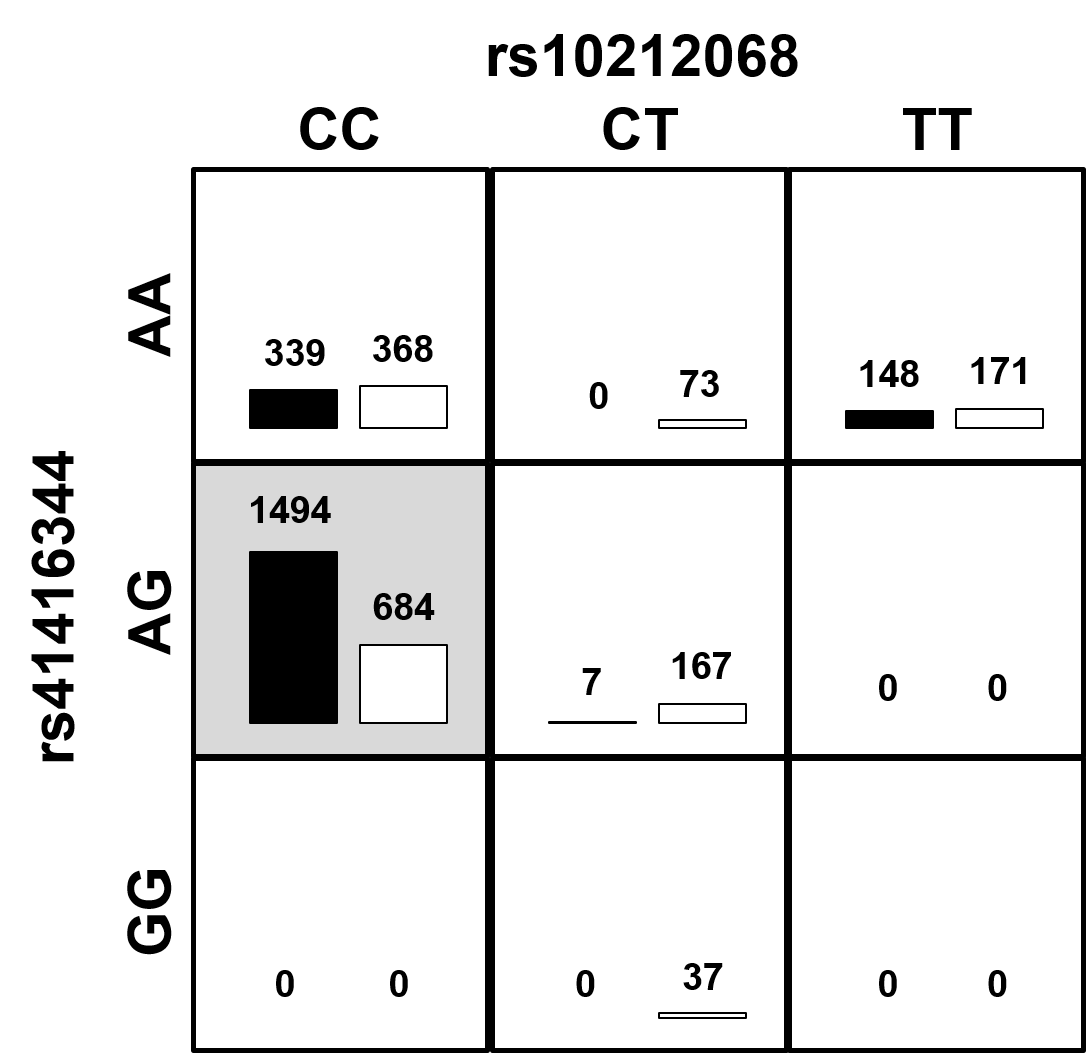 | 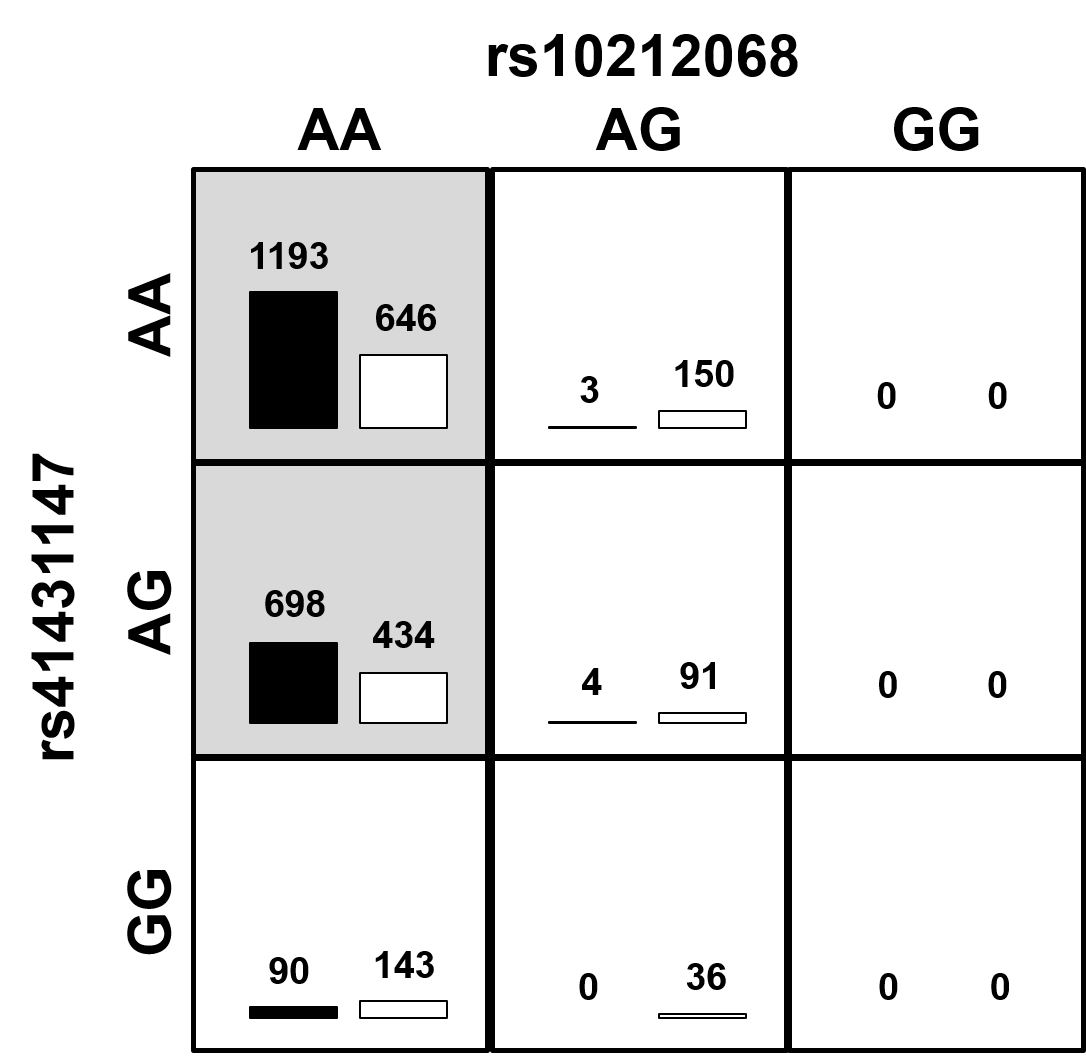 |
| --- | --- |
| 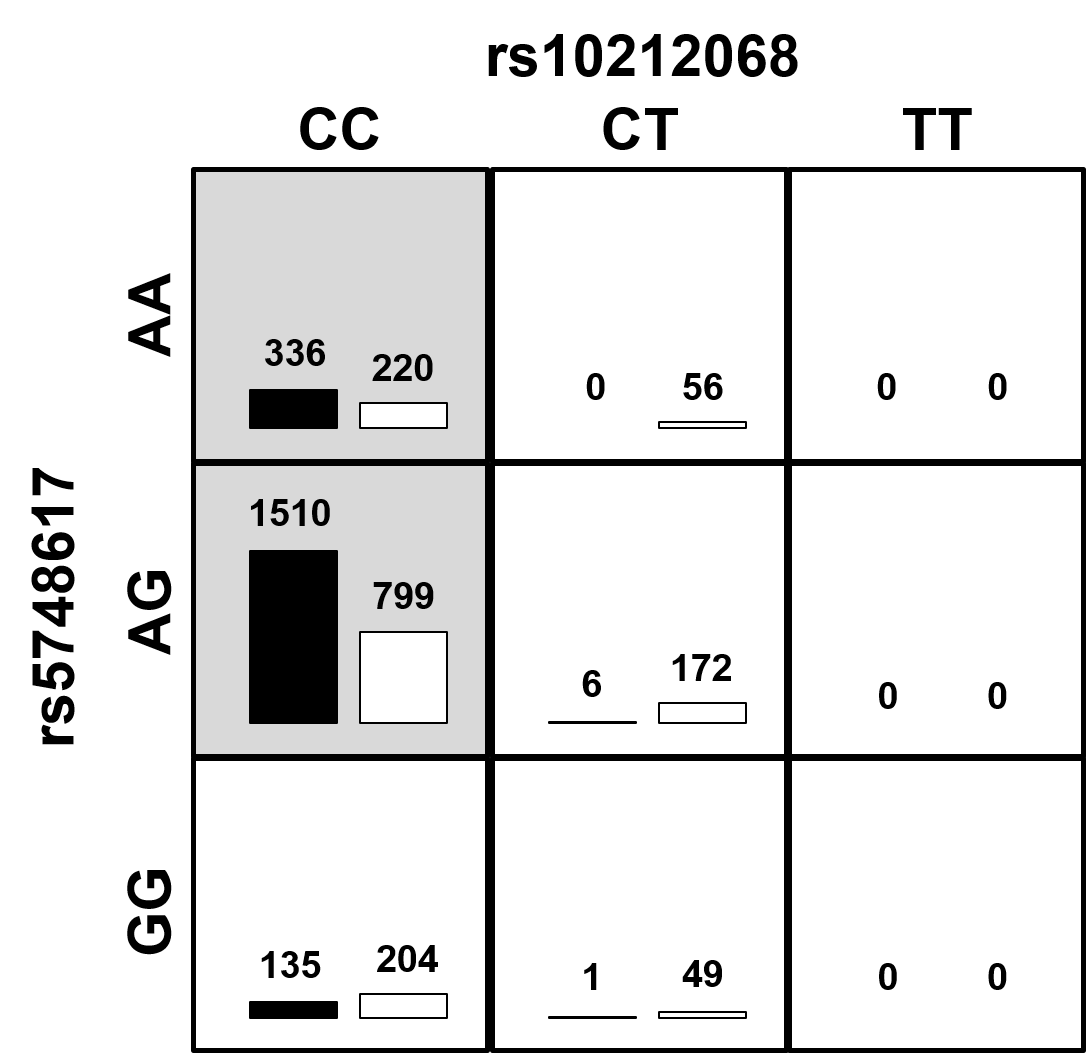 | 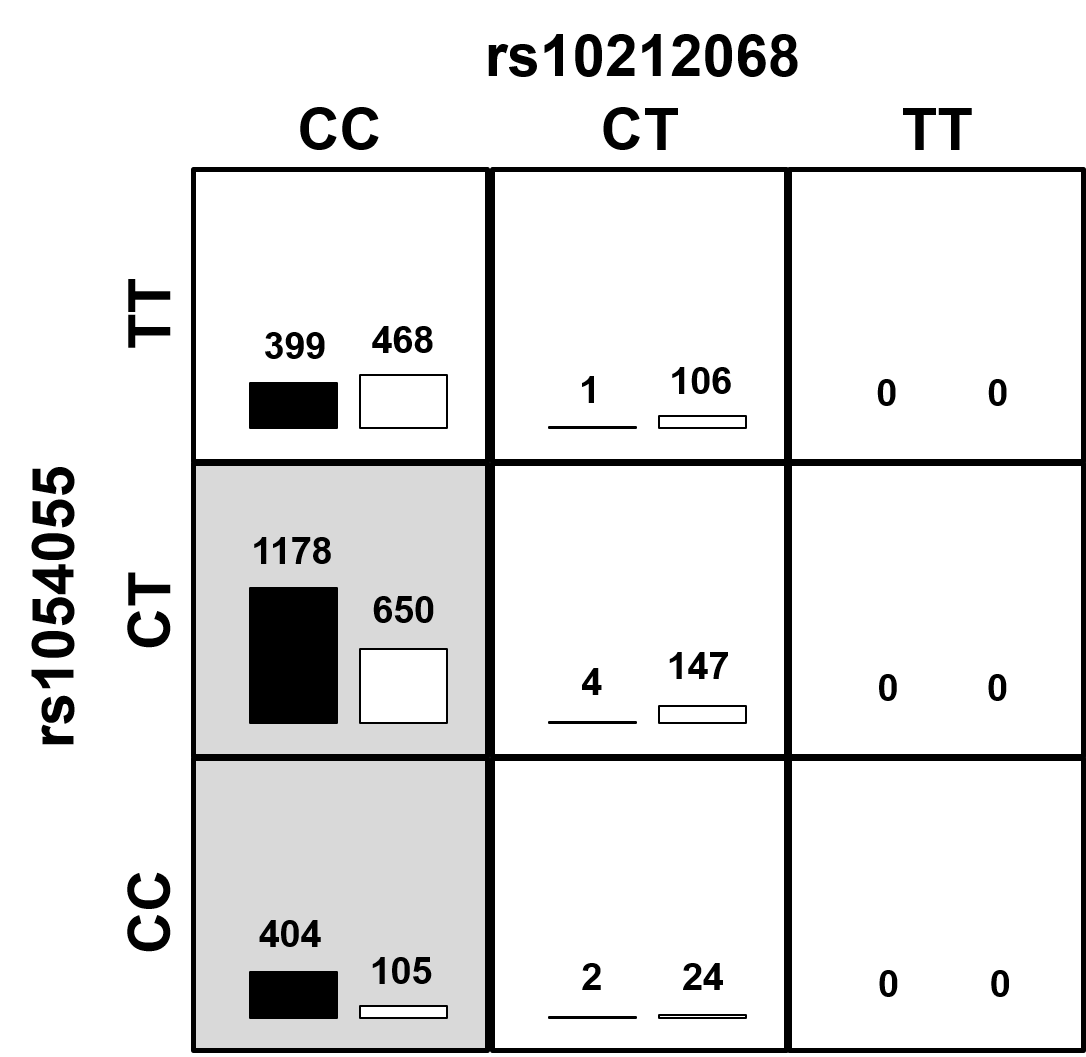 |
| 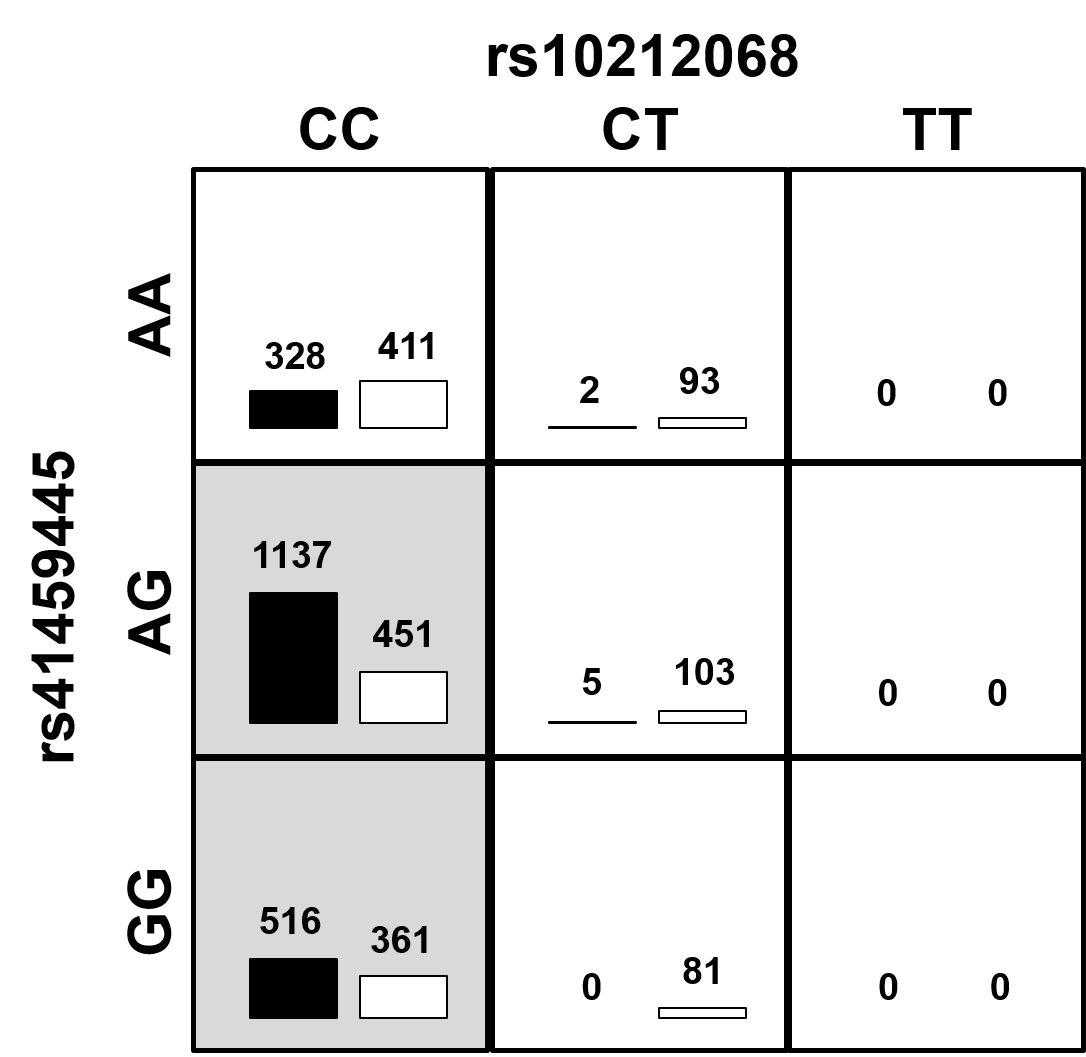 | 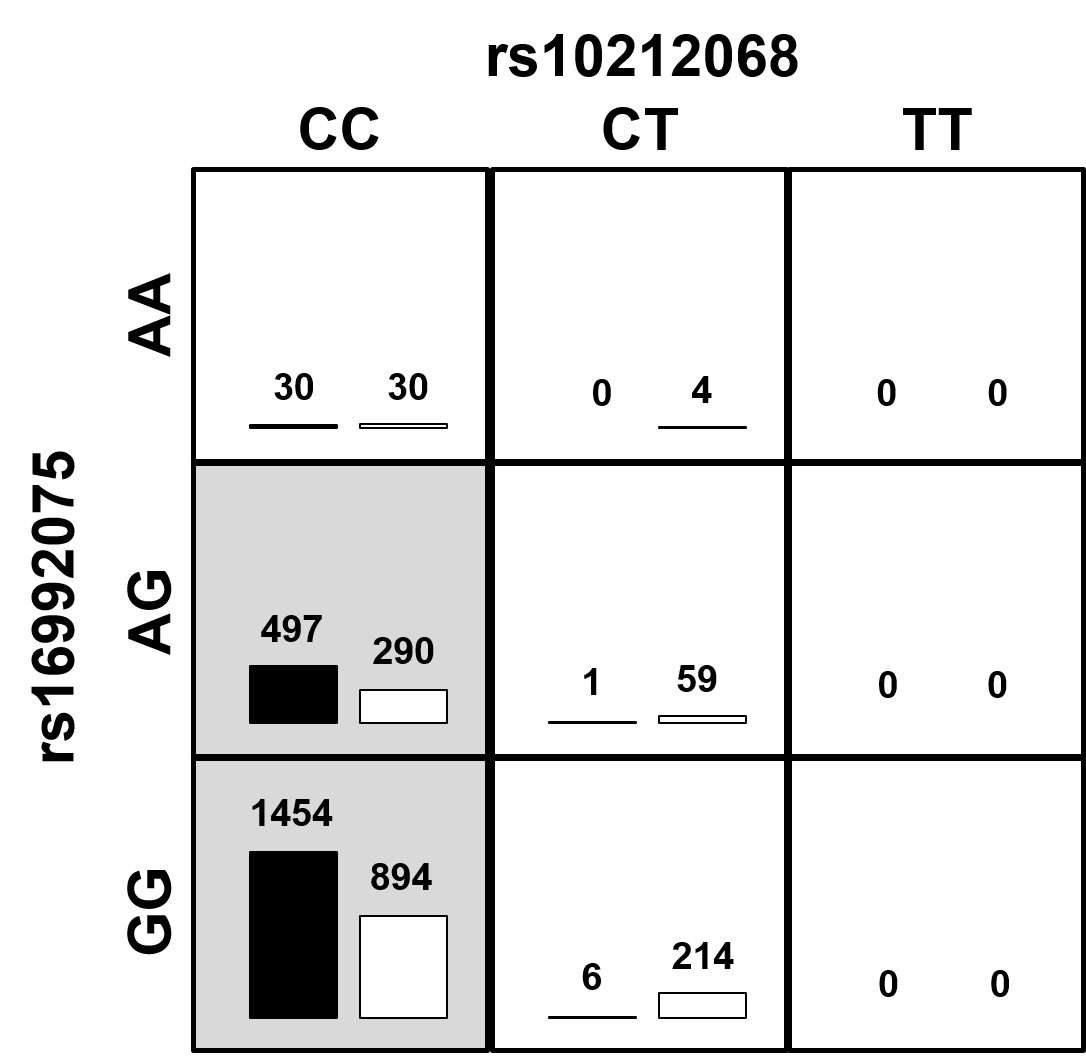 |

**Chromosome 23**


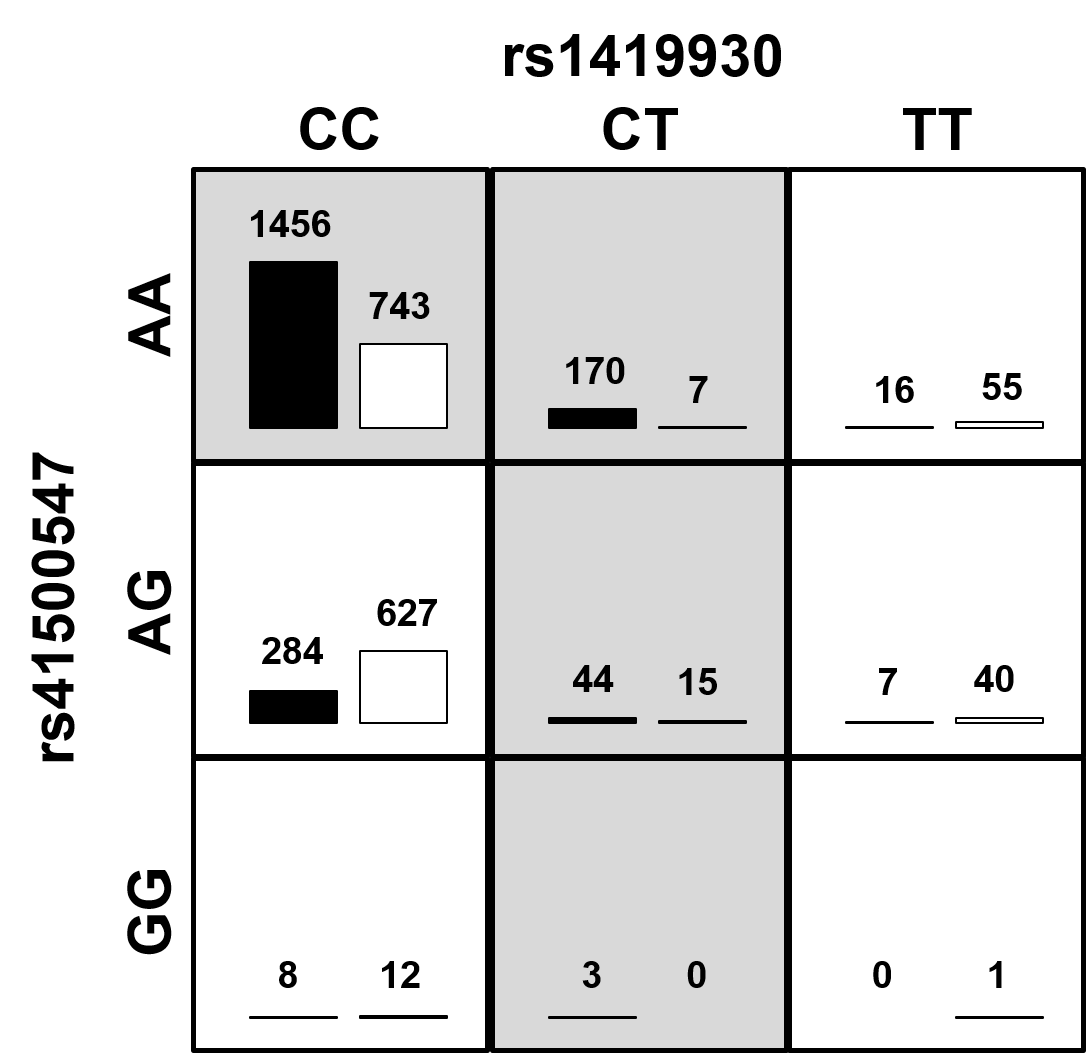


**Example of MODEMDR procedures**

In this example, suppose a data includes 100 samples (50 cases and 50 controls) and the number of factors is 5. Table 1 list a dataset. From left to right, the columns list the control/case class and SNPs. We use six genomes to illustrate the MODEMDR process as follows:

1. Data pre-processing,
2. Pareto operation,
3. Target vectors initialization,
4. Mutation operation, recombination operation, boundary constraints, and selection operation,
5. Evaluate whether stopping criteria are met.

**1. Data pre-processing**

1. Classification generate the balanced 5-fold subsets.

**Step 1.** Classify the samples into case sets (cases) and controls sets (controls).

| No. | Control | | | | | No. | Case | | | | |
| --- | --- | --- | --- | --- | --- | --- | --- | --- | --- | --- | --- |
|  | SNP1 | SNP2 | SNP3 | SNP4 | SNP5 |  | SNP1 | SNP2 | SNP3 | SNP4 | SNP5 |
| 1 | 0 | 1 | 0 | 0 | 0 | 1 | 0 | 0 | 0 | 0 | 0 |
| 2 | 0 | 0 | 1 | 2 | 0 | 2 | 0 | 0 | 0 | 0 | 0 |
| 3 | 0 | 0 | 1 | 1 | 0 | 3 | 0 | 0 | 1 | 0 | 0 |
| 4 | 0 | 1 | 0 | 2 | 0 | 4 | 0 | 0 | 0 | 1 | 0 |
| 5 | 1 | 0 | 1 | 1 | 0 | 5 | 1 | 2 | 1 | 0 | 0 |
| 6 | 0 | 1 | 2 | 0 | 0 | 6 | 0 | 0 | 1 | 0 | 0 |
| 7 | 0 | 1 | 1 | 1 | 0 | 7 | 0 | 0 | 1 | 2 | 0 |
| 8 | 0 | 0 | 1 | 0 | 0 | 8 | 1 | 1 | 1 | 0 | 0 |
| 9 | 0 | 0 | 2 | 2 | 0 | 9 | 0 | 0 | 1 | 1 | 0 |
| 10 | 0 | 0 | 1 | 2 | 0 | 10 | 1 | 1 | 1 | 1 | 0 |
| 11 | 0 | 0 | 1 | 1 | 0 | 11 | 0 | 0 | 2 | 0 | 0 |
| 12 | 0 | 0 | 0 | 2 | 0 | 12 | 0 | 0 | 0 | 1 | 0 |
| 13 | 2 | 0 | 1 | 1 | 0 | 13 | 1 | 0 | 1 | 2 | 1 |
| 14 | 0 | 0 | 1 | 1 | 0 | 14 | 0 | 0 | 2 | 1 | 0 |
| 15 | 0 | 0 | 1 | 2 | 1 | 15 | 0 | 0 | 1 | 0 | 1 |
| 16 | 1 | 0 | 2 | 0 | 0 | 16 | 0 | 0 | 0 | 0 | 0 |
| 17 | 0 | 0 | 2 | 0 | 0 | 17 | 0 | 0 | 0 | 2 | 0 |
| 18 | 0 | 0 | 2 | 1 | 1 | 18 | 0 | 0 | 0 | 1 | 0 |
| 19 | 0 | 0 | 1 | 1 | 0 | 19 | 0 | 0 | 0 | 0 | 0 |
| 20 | 0 | 0 | 1 | 0 | 0 | 20 | 1 | 1 | 2 | 1 | 1 |
| 21 | 0 | 0 | 1 | 1 | 0 | 21 | 1 | 1 | 1 | 1 | 1 |
| 22 | 0 | 0 | 1 | 0 | 0 | 22 | 0 | 0 | 1 | 1 | 1 |
| 23 | 0 | 0 | 1 | 1 | 0 | 23 | 0 | 0 | 2 | 1 | 0 |
| 24 | 0 | 0 | 2 | 0 | 0 | 24 | 0 | 0 | 0 | 2 | 0 |
| 25 | 1 | 1 | 1 | 2 | 0 | 25 | 1 | 0 | 1 | 0 | 0 |
| 26 | 0 | 1 | 0 | 2 | 0 | 26 | 0 | 0 | 1 | 1 | 1 |
| 27 | 1 | 0 | 1 | 1 | 1 | 27 | 0 | 0 | 1 | 1 | 0 |
| 28 | 0 | 0 | 2 | 0 | 0 | 28 | 1 | 1 | 0 | 2 | 0 |
| 29 | 0 | 0 | 0 | 2 | 0 | 29 | 0 | 0 | 2 | 0 | 1 |
| 30 | 0 | 0 | 0 | 2 | 0 | 30 | 0 | 0 | 2 | 1 | 0 |
| 31 | 0 | 0 | 1 | 0 | 0 | 31 | 0 | 0 | 0 | 2 | 0 |
| 32 | 0 | 0 | 1 | 0 | 0 | 32 | 0 | 0 | 1 | 0 | 0 |
| 33 | 1 | 0 | 0 | 0 | 1 | 33 | 0 | 0 | 0 | 0 | 0 |
| 34 | 0 | 1 | 1 | 1 | 1 | 34 | 0 | 0 | 2 | 0 | 0 |
| 35 | 1 | 0 | 0 | 1 | 0 | 35 | 0 | 0 | 1 | 0 | 0 |
| 36 | 0 | 1 | 1 | 0 | 0 | 36 | 1 | 1 | 1 | 0 | 0 |
| 37 | 0 | 0 | 1 | 2 | 0 | 37 | 0 | 0 | 1 | 1 | 0 |
| 38 | 0 | 0 | 2 | 2 | 0 | 38 | 0 | 0 | 1 | 1 | 0 |
| 39 | 0 | 0 | 0 | 2 | 0 | 39 | 0 | 0 | 0 | 0 | 0 |
| 40 | 0 | 1 | 1 | 1 | 0 | 40 | 0 | 0 | 0 | 1 | 0 |
| 41 | 0 | 0 | 1 | 1 | 1 | 41 | 0 | 0 | 2 | 2 | 1 |
| 42 | 0 | 0 | 0 | 1 | 0 | 42 | 0 | 0 | 1 | 1 | 0 |
| 43 | 0 | 1 | 0 | 0 | 0 | 43 | 0 | 0 | 1 | 0 | 0 |
| 44 | 0 | 1 | 0 | 1 | 0 | 44 | 1 | 1 | 0 | 1 | 0 |
| 45 | 0 | 0 | 0 | 0 | 1 | 45 | 0 | 0 | 0 | 0 | 0 |
| 46 | 0 | 0 | 0 | 1 | 0 | 46 | 0 | 0 | 1 | 1 | 1 |
| 47 | 0 | 0 | 0 | 2 | 0 | 47 | 0 | 0 | 0 | 0 | 0 |
| 48 | 0 | 0 | 0 | 0 | 1 | 48 | 0 | 0 | 2 | 0 | 0 |
| 49 | 0 | 0 | 1 | 1 | 0 | 49 | 0 | 0 | 0 | 0 | 1 |
| 50 | 0 | 1 | 1 | 2 | 0 | 50 | 2 | 1 | 2 | 1 | 0 |

**Step 2.** Randomly sort samples in cases and samples in controls.

| No. | Control | | | | | No. | Case | | | | |
| --- | --- | --- | --- | --- | --- | --- | --- | --- | --- | --- | --- |
|  | SNP1 | SNP2 | SNP3 | SNP4 | SNP5 |  | SNP1 | SNP2 | SNP3 | SNP4 | SNP5 |
| 1 | 0 | 0 | 1 | 0 | 0 | 1 | 0 | 2 | 0 | 0 | 0 |
| 2 | 2 | 0 | 0 | 0 | 0 | 2 | 1 | 0 | 0 | 0 | 0 |
| 3 | 1 | 2 | 1 | 0 | 0 | 3 | 2 | 1 | 0 | 2 | 1 |
| 4 | 0 | 2 | 0 | 0 | 1 | 4 | 1 | 1 | 1 | 0 | 0 |
| 5 | 2 | 0 | 0 | 1 | 0 | 5 | 0 | 2 | 0 | 0 | 0 |
| 6 | 1 | 1 | 0 | 0 | 0 | 6 | 2 | 0 | 0 | 0 | 0 |
| 7 | 2 | 0 | 0 | 0 | 0 | 7 | 1 | 2 | 0 | 0 | 0 |
| 8 | 2 | 1 | 1 | 0 | 0 | 8 | 2 | 0 | 1 | 0 | 0 |
| 9 | 1 | 2 | 0 | 1 | 1 | 9 | 1 | 1 | 0 | 1 | 1 |
| 10 | 1 | 0 | 0 | 0 | 0 | 10 | 2 | 2 | 1 | 0 | 0 |
| 11 | 1 | 2 | 0 | 0 | 0 | 11 | 0 | 1 | 0 | 0 | 0 |
| 12 | 1 | 1 | 1 | 0 | 1 | 12 | 0 | 1 | 0 | 0 | 0 |
| 13 | 0 | 1 | 0 | 0 | 1 | 13 | 1 | 0 | 0 | 0 | 0 |
| 14 | 0 | 2 | 0 | 0 | 1 | 14 | 1 | 1 | 0 | 0 | 0 |
| 15 | 0 | 1 | 0 | 0 | 0 | 15 | 0 | 0 | 0 | 0 | 0 |
| 16 | 1 | 1 | 0 | 0 | 0 | 16 | 2 | 1 | 0 | 0 | 0 |
| 17 | 1 | 1 | 0 | 0 | 0 | 17 | 1 | 2 | 1 | 1 | 0 |
| 18 | 1 | 1 | 0 | 2 | 0 | 18 | 0 | 0 | 0 | 0 | 0 |
| 19 | 2 | 2 | 0 | 0 | 0 | 19 | 1 | 1 | 0 | 0 | 0 |
| 20 | 2 | 0 | 0 | 0 | 1 | 20 | 0 | 0 | 1 | 0 | 0 |
| 21 | 1 | 1 | 0 | 0 | 0 | 21 | 1 | 1 | 0 | 0 | 0 |
| 22 | 1 | 1 | 1 | 0 | 0 | 22 | 2 | 1 | 1 | 1 | 1 |
| 23 | 2 | 0 | 0 | 0 | 0 | 23 | 0 | 0 | 0 | 0 | 0 |
| 24 | 0 | 2 | 0 | 0 | 0 | 24 | 1 | 0 | 0 | 0 | 0 |
| 25 | 0 | 0 | 1 | 1 | 0 | 25 | 1 | 0 | 0 | 0 | 0 |
| 26 | 0 | 0 | 1 | 0 | 0 | 26 | 1 | 0 | 0 | 0 | 0 |
| 27 | 1 | 0 | 0 | 0 | 0 | 27 | 0 | 0 | 0 | 0 | 0 |
| 28 | 1 | 2 | 0 | 0 | 1 | 28 | 0 | 2 | 0 | 0 | 0 |
| 29 | 1 | 1 | 0 | 0 | 0 | 29 | 0 | 0 | 0 | 0 | 0 |
| 30 | 1 | 1 | 1 | 1 | 0 | 30 | 1 | 1 | 0 | 0 | 0 |
| 31 | 1 | 0 | 0 | 0 | 0 | 31 | 0 | 0 | 0 | 0 | 0 |
| 32 | 0 | 2 | 0 | 0 | 0 | 32 | 1 | 0 | 0 | 1 | 1 |
| 33 | 0 | 0 | 0 | 0 | 1 | 33 | 1 | 1 | 0 | 0 | 0 |
| 34 | 0 | 2 | 0 | 0 | 0 | 34 | 2 | 1 | 0 | 0 | 0 |
| 35 | 1 | 1 | 0 | 0 | 1 | 35 | 2 | 0 | 0 | 0 | 0 |
| 36 | 1 | 1 | 0 | 0 | 0 | 36 | 0 | 0 | 0 | 0 | 0 |
| 37 | 1 | 2 | 0 | 0 | 0 | 37 | 1 | 1 | 1 | 1 | 1 |
| 38 | 0 | 2 | 0 | 0 | 0 | 38 | 0 | 1 | 0 | 0 | 0 |
| 39 | 0 | 1 | 0 | 0 | 0 | 39 | 0 | 2 | 0 | 1 | 1 |
| 40 | 1 | 2 | 0 | 0 | 0 | 40 | 1 | 0 | 0 | 1 | 2 |
| 41 | 2 | 2 | 0 | 0 | 0 | 41 | 1 | 0 | 1 | 0 | 0 |
| 42 | 1 | 1 | 0 | 0 | 0 | 42 | 0 | 1 | 0 | 1 | 1 |
| 43 | 0 | 1 | 0 | 1 | 0 | 43 | 0 | 0 | 0 | 0 | 0 |
| 44 | 1 | 1 | 0 | 0 | 1 | 44 | 2 | 1 | 0 | 0 | 0 |
| 45 | 1 | 1 | 0 | 1 | 0 | 45 | 1 | 1 | 1 | 0 | 0 |
| 46 | 0 | 0 | 0 | 0 | 1 | 46 | 2 | 0 | 0 | 0 | 0 |
| 47 | 1 | 0 | 0 | 0 | 0 | 47 | 1 | 0 | 0 | 1 | 0 |
| 48 | 1 | 0 | 0 | 0 | 0 | 48 | 1 | 1 | 1 | 0 | 0 |
| 49 | 0 | 2 | 0 | 0 | 0 | 49 | 0 | 1 | 0 | 0 | 0 |
| 50 | 1 | 0 | 0 | 0 | 1 | 50 | 1 | 0 | 0 | 1 | 1 |

**Step 3.** Count total number of cases and total number of controls.

The total number of cases is 50 and the total number of controls is 50.

**Step 4.** Compute the ratio between cases and controls.

Ratio = 50 / 50 =1, meaning the data is balanced between cases and controls.

**Step 5.** Classify the samples of cases and samples of controls into *j*^th^-fold subset according to ratio.

| No. | Control | | | | | No. | Case | | | | |
| --- | --- | --- | --- | --- | --- | --- | --- | --- | --- | --- | --- |
|  | SNP1 | SNP2 | SNP3 | SNP4 | SNP5 |  | SNP1 | SNP2 | SNP3 | SNP4 | SNP5 |
| 1-fold | | | | | | | | | | | |
| 1 | 0 | 0 | 1 | 0 | 0 | 1 | 0 | 2 | 0 | 0 | 0 |
| 2 | 2 | 0 | 0 | 0 | 0 | 2 | 1 | 0 | 0 | 0 | 0 |
| 3 | 1 | 2 | 1 | 0 | 0 | 3 | 2 | 1 | 0 | 2 | 1 |
| 4 | 0 | 2 | 0 | 0 | 1 | 4 | 1 | 1 | 1 | 0 | 0 |
| 5 | 2 | 0 | 0 | 1 | 0 | 5 | 0 | 2 | 0 | 0 | 0 |
| 6 | 1 | 1 | 0 | 0 | 0 | 6 | 2 | 0 | 0 | 0 | 0 |
| 7 | 2 | 0 | 0 | 0 | 0 | 7 | 1 | 2 | 0 | 0 | 0 |
| 8 | 2 | 1 | 1 | 0 | 0 | 8 | 2 | 0 | 1 | 0 | 0 |
| 9 | 1 | 2 | 0 | 1 | 1 | 9 | 1 | 1 | 0 | 1 | 1 |
| 10 | 1 | 0 | 0 | 0 | 0 | 10 | 2 | 2 | 1 | 0 | 0 |
| 2-fold | | | | | | | | | | | |
| 11 | 1 | 2 | 0 | 0 | 0 | 11 | 0 | 1 | 0 | 0 | 0 |
| 12 | 1 | 1 | 1 | 0 | 1 | 12 | 0 | 1 | 0 | 0 | 0 |
| 13 | 0 | 1 | 0 | 0 | 1 | 13 | 1 | 0 | 0 | 0 | 0 |
| 14 | 0 | 2 | 0 | 0 | 1 | 14 | 1 | 1 | 0 | 0 | 0 |
| 15 | 0 | 1 | 0 | 0 | 0 | 15 | 0 | 0 | 0 | 0 | 0 |
| 16 | 1 | 1 | 0 | 0 | 0 | 16 | 2 | 1 | 0 | 0 | 0 |
| 17 | 1 | 1 | 0 | 0 | 0 | 17 | 1 | 2 | 1 | 1 | 0 |
| 18 | 1 | 1 | 0 | 2 | 0 | 18 | 0 | 0 | 0 | 0 | 0 |
| 19 | 2 | 2 | 0 | 0 | 0 | 19 | 1 | 1 | 0 | 0 | 0 |
| 20 | 2 | 0 | 0 | 0 | 1 | 20 | 0 | 0 | 1 | 0 | 0 |
| 3-fold | | | | | | | | | | | |
| 21 | 1 | 1 | 0 | 0 | 0 | 21 | 1 | 1 | 0 | 0 | 0 |
| 22 | 1 | 1 | 1 | 0 | 0 | 22 | 2 | 1 | 1 | 1 | 1 |
| 23 | 2 | 0 | 0 | 0 | 0 | 23 | 0 | 0 | 0 | 0 | 0 |
| 24 | 0 | 2 | 0 | 0 | 0 | 24 | 1 | 0 | 0 | 0 | 0 |
| 25 | 0 | 0 | 1 | 1 | 0 | 25 | 1 | 0 | 0 | 0 | 0 |
| 26 | 0 | 0 | 1 | 0 | 0 | 26 | 1 | 0 | 0 | 0 | 0 |
| 27 | 1 | 0 | 0 | 0 | 0 | 27 | 0 | 0 | 0 | 0 | 0 |
| 28 | 1 | 2 | 0 | 0 | 1 | 28 | 0 | 2 | 0 | 0 | 0 |
| 29 | 1 | 1 | 0 | 0 | 0 | 29 | 0 | 0 | 0 | 0 | 0 |
| 30 | 1 | 1 | 1 | 1 | 0 | 30 | 1 | 1 | 0 | 0 | 0 |
| 4-fold | | | | | | | | | | | |
| 31 | 1 | 0 | 0 | 0 | 0 | 31 | 0 | 0 | 0 | 0 | 0 |
| 32 | 0 | 2 | 0 | 0 | 0 | 32 | 1 | 0 | 0 | 1 | 1 |
| 33 | 0 | 0 | 0 | 0 | 1 | 33 | 1 | 1 | 0 | 0 | 0 |
| 34 | 0 | 2 | 0 | 0 | 0 | 34 | 2 | 1 | 0 | 0 | 0 |
| 35 | 1 | 1 | 0 | 0 | 1 | 35 | 2 | 0 | 0 | 0 | 0 |
| 36 | 1 | 1 | 0 | 0 | 0 | 36 | 0 | 0 | 0 | 0 | 0 |
| 37 | 1 | 2 | 0 | 0 | 0 | 37 | 1 | 1 | 1 | 1 | 1 |
| 38 | 0 | 2 | 0 | 0 | 0 | 38 | 0 | 1 | 0 | 0 | 0 |
| 39 | 0 | 1 | 0 | 0 | 0 | 39 | 0 | 2 | 0 | 1 | 1 |
| 40 | 1 | 2 | 0 | 0 | 0 | 40 | 1 | 0 | 0 | 1 | 2 |
| 5-fold | | | | | | | | | | | |
| 41 | 2 | 2 | 0 | 0 | 0 | 41 | 1 | 0 | 1 | 0 | 0 |
| 42 | 1 | 1 | 0 | 0 | 0 | 42 | 0 | 1 | 0 | 1 | 1 |
| 43 | 0 | 1 | 0 | 1 | 0 | 43 | 0 | 0 | 0 | 0 | 0 |
| 44 | 1 | 1 | 0 | 0 | 1 | 44 | 2 | 1 | 0 | 0 | 0 |
| 45 | 1 | 1 | 0 | 1 | 0 | 45 | 1 | 1 | 1 | 0 | 0 |
| 46 | 0 | 0 | 0 | 0 | 1 | 46 | 2 | 0 | 0 | 0 | 0 |
| 47 | 1 | 0 | 0 | 0 | 0 | 47 | 1 | 0 | 0 | 1 | 0 |
| 48 | 1 | 0 | 0 | 0 | 0 | 48 | 1 | 1 | 1 | 0 | 0 |
| 49 | 0 | 2 | 0 | 0 | 0 | 49 | 0 | 1 | 0 | 0 | 0 |
| 50 | 1 | 0 | 0 | 0 | 1 | 50 | 1 | 0 | 0 | 1 | 1 |

**2. Pareto operation**

In the initialization, an empty storage *S* = {∅} is generated.

**3. Target vectors initialization**

**Target vectors are generated by the combined genetic and environmental factors, in which the parameters in a target vector are mutually different. Thus, six target vector are randomly generated:**

| Initial target vector_1_:  *X*_1,0_ = (1, 3), meaning (*SNP­*_1_, *SNP*_3_). | Initial target vector_2_:  *X* _2,0_ = (2, 3), meaning (*SNP­*_2_, *SNP­*_3_) |
| --- | --- |
| Initial target vector_3_:  *X* _3,0_ = (1, 5), meaning (*SNP­*_1_, *SNP­*_5_) | Initial target vector_4_:  *X* _4,0_ = (3, 4), meaning (*SNP*_3_*, SNP*_4_) |
| Initial target vector_5_:  *X* _5,0_ = (2, 4), meaning (*SNP­*_2_, *SNP*_4_) | Initial target vector_6_:  *X* _6,0_ = (3, 5), meaning (*SNP­*_3_, *SNP­*_5_) |

**3. Mutation operation, recombination operation, boundary constraints, selection operation, and evaluating whether stopping criteria are met.**

3.1. Mutation operation

The mutant vector *V_i,G_*_+1_ is generated according to Eq. 2. In the initialization, the Pareto storage is empty. Therefore, the *X_r_*_1,0_, *X_r_*_2,0_ and *X_r_*_2,0_ are selected from population.

Therefore,

Mutation operation of target vector_1_:

Suppose *r*_1_, *r*_2_, and *r*_3_ randomly select 3, 4, and 2, respectively, and *F* set to 0.5.

*V_i,G+_*_1_ = *X_r_*_1_*_,G_* + *F‧*(*X_r_*_2_*_,G_* – *X_r_*_3_*_,G_*)

*=> V*_1,0+1_ = *X*_3,0_ + 0.5*‧*(*X*_4,0_ – *X*_2,0_)

=> *V*_1,1_ = (1, 5) + 0.5*‧*{(3, 4) – (2, 3)}

=> *V*_1,1_ = ((1 + 0.5*‧*(3 – 2)), (5 + 0.5*‧*(4 – 3)))

=> *V*_1,1_ = (1.5, 5.5)

Mutation operation of target vector_2_:

Suppose *r*_1_, *r*_2_, and *r*_3_ randomly select 1, 3, and 6, respectively.

*V_i,G+_*_1_ = *X_r_*_1_*_,G_* + *F‧*(*X_r_*_2_*_,G_* – *X_r_*_3_*_,G_*)

*=> V*_2, 0+1_ = *X*_1,0_ + 0.5*‧*(*X*_3,0_ – *X*_6,0_)

=> *V*_2, 1_ = (1, 3) + 0.5*‧*{(1, 5) – (3, 5)}

=> *V*_2, 1_ = ((1 + 0.5*‧*(1 – 3)), (3 + 0.5*‧*(5 – 5)))

=> *V*_2, 1_ = (0, 3)

Mutation operation of target vector_3_:

Suppose *r*_1_, *r*_2_, and *r*_3_ randomly select 5, 2, and 4, respectively.

*V_i,G_*_+1_ = *X_r_*_1_,*_G_* + *F‧*(*X_r_*_2_*_,G_* – *X_r_*_5_*_,G_*)

*=> V*_3,0+1_ = *X*_5,0_ + 0.5*‧*(*X*_2,0_ – *X*_4,0_)

=> *V*_3,1_ = (2, 4) + 0.5*‧*{(2, 3) – (3, 4)}

=> *V*_3,1_ = ((2 + 0.5*‧*(2 – 3)), (4 + 0.5*‧*(3 – 4)))

=> *V*_3,1_ = (1.5, 3.5)

Mutation operation of target vector_4_:

Suppose *r*_1_, *r*_2_, and *r*_3_ randomly select 2, 6, and 3, respectively.

*V_i,G_*_+1_ = *X_r_*_1_*_,G_* + *F‧*(*X_r_*_2_*_,G_* – *X_r_*_3_*_,G_*)

*=> V*_4,0+1_ = *X*_2,0_ + 0.5*‧*(*X*_6,0_ – *X*_3,0_)

=> *V*_4,1_ = (2, 3) + 0.5*‧*{(3, 5) – (1, 5)}

=> *V*_4,1_ = ((2 + 0.5*‧*(3 – 1)), (3 + 0.5*‧*(5 – 5)))

=> *V*_4,1_ = (1, 3)

Mutation operation of target vector_5_:

Suppose *r*_1_, *r*_2_, and *r*_3_ randomly select 6, 1, and 4, respectively.

*V_i,G_*_+1_ = *X_r_*_1_*_,G_* + *F‧*(*X_r_*_2_*_,G_* – *X_r_*_3_*_,G_*)

*=> V*_5,0+1_ = *X*_6,0_ + 0.5*‧*(*X*_1,0_ – *X*_4,0_)

=> *V*_5,1_ = (3, 5) + 0.5*‧*{(1, 3) – (3, 4)}

=> *V*_5,1_ = ((3 + 0.5*‧*(1 – 3)), (5 + 0.5*‧*(3 – 4)))

=> *V*_5,1_ = (2, 4.5)

Mutation operation of target vector_6_:

Suppose *r*_1_, *r*_2_, and *r*_3_ randomly select 3, 5, and 2, respectively.

*V_i,G_*_+1_ = *X_r_*_1_*_,G_* + *F‧*(*X_r_*_2_*_,G_* – *X_r_*_3_*_,G_*)

*=> V*_6,0+1_ = *X*_3,0_ + 0.5*‧*(*X*_5,0_ – *X*_2,0_)

=> *V*_6,1_ = (1, 5) + 0.5*‧*{(2, 4) – (2, 3)}

=> *V*_6,1_ = ((1 + 0.5*‧*(2 – 2)), (5 + 0.5*‧*(4 – 3)))

=> *V*_6,1_ = (1, 5.5)

**3.2.** Recombination operation

The trial vector *U_i_*,*_G_*_+1_ is developed from the parameters of the target vector, *X_i_*,*_G_*, and the parameters of the mutant vector, *V_i_*,*_G_*_+1_. The trial vector can be denoted as:

*U_i,G+1_* = (*u*_1_*_i,G+_*_1_, *u*_2_*_i,G+_*_1_, ..., *u_Di,G+_*_1_)

where

$$u_{j,i,G+1}=\left\{\begin{matrix} v_{j,i,G+1} & \text{if}(randb(j)\leq CR)\text{ or }j=rnbr(i), \\ x_{j,i,G\text{ }} & \text{if}(randb(j)>CR)\text{ or }j\neq rnbr(i), \end{matrix}\right.\text{ }j=1,\text{ }2,\text{ }...,\text{ }D$$

Recombination of target vector_1_:

Suppose a randomly generated *randb* = {0.6, 0.7}

No dimension is smaller than *CR* (0.5).

Therefore,

*U*_1_,*_G_*_+1_ = (*X*_1,1,0_, *X*_2,1,0_)

*=> U*_1,1_ = (1, 3)

Recombination of target vector_2_:

Suppose a randomly generated *randb* = {0.4, 0.1}

The 1^th^ and 2^th^ dimensions are smaller than *CR* (0.5).

Therefore,

*U*_2_,*_G_*_+1_ = (*V*_1,2,0+1_, *V*_2,2,0+1_)

*=> U*_2,1_ = (0, 3)

Recombination of target vector_3_:

Suppose a randomly generated *randb* = {0.4, 0.8}

The 1^th^ dimension is smaller than *CR* (0.5).

Therefore,

*U*_3_,*_G_*_+1_ = (*V*_1,3,0+1_, *X*_2,3,0_)

*=> U*_3,1_ = (1.5, 5)

Recombination of target vector_4_:

Suppose a randomly generated *randb* = {0.1, 0.9}

The 1^th^ dimension is smaller than *CR* (0.5).

Therefore,

*U*_4_,*_G_*_+1_ = (*V*_1,4,0+1_, *X*_2,4,0_)

*=> U*_4,1_ = (1, 4)

Recombination of target vector_5_:

Suppose a randomly generated *randb* = {0.6, 0.7}

No dimension is smaller than *CR* (0.5).

Therefore,

*U*_5_,*_G_*_+1_ = (*X*_1,5,0_, *X*_2,5,0_)

*=> U*_5,1_ = (2, 4.5)

Recombination of target vector_6_:

Suppose a randomly generated *randb* = {0.4, 0.6}

The 1^th^ dimensions are smaller than *CR* (0.5).

Therefore,

*U*_6_,*_G_*_+1_ = (*V*_1,6,0+1_, *X*_2,6,0_)

*=> U*_6,1_ = (1, 5)

**3.3.** Boundary constraints

In the recombination operation, the new recombination genome may extend the search outside of the initiated range of the problem search space and will not satisfy the rule that SNPs are mutually different. We use Eq. (6) to constrain each trial vector.

$$u_{j,i,G+1}=\left\{\begin{matrix} rand_{j}\times[0,\text{ }1)\times(upper-lower)+lower, & \text{if (}u_{j,i,G}<lower\text{ or }u_{j,i,G}>upper)\text{ or }(\exists!u_{j,i,G}\in U_{i,G}) \\ u_{j,i,G}\text{, } & \text{otherwise } \end{matrix}\right.$$

*U*_1,1_ = (1, 3)

*U*_2,1_ = (0, 3) => (**5**, 3)

*U*_3,1_ = (1.5, 5) => (2, 5)

*U*_4,1_ = (1, 4)

*U*_5,1_ = (2, 4.5) => (2, 5)

*U*_6,1_ = (1, 5)

**3.4.** Selection operation

The selection operation determines whether or not the trial vector *U_i_*,*_G_* should become a member of generation *G*+1. The trial vector *U_i_*,*_G_*_+1_ is compared to the target vector *X_i_*,*_G_* using the greedy criterion. If trial vector *U_i_*,*_G_*_+1_ yielded a better function value than *X_i_*,*_G_*, then *X_i_*,*_G_*_+1_ is set as *U_i_*,*_G_*_+1_; otherwise, *X_i_*,*_G_* is retained. Therefore, the fitness values of the target vectors and trial vectors can be calculated by fitness evaluation. Here, we example *X*_1,0_ = (1, 3) in the first CV as below:

Select 1-fold subset for testing data and the remaining as training data.

| No. | Control | | | | | No. | Case | | | | |
| --- | --- | --- | --- | --- | --- | --- | --- | --- | --- | --- | --- |
|  | SNP1 | SNP2 | SNP3 | SNP4 | SNP5 |  | SNP1 | SNP2 | SNP3 | SNP4 | SNP5 |
| **1-fold (testing data)** | | | | | | | | | | | |
| 1 | 0 | 0 | 1 | 0 | 0 | 1 | 0 | 2 | 0 | 0 | 0 |
| 2 | 2 | 0 | 0 | 0 | 0 | 2 | 1 | 0 | 0 | 0 | 0 |
| 3 | 1 | 2 | 1 | 0 | 0 | 3 | 2 | 1 | 0 | 2 | 1 |
| 4 | 0 | 2 | 0 | 0 | 1 | 4 | 1 | 1 | 1 | 0 | 0 |
| 5 | 2 | 0 | 0 | 1 | 0 | 5 | 0 | 2 | 0 | 0 | 0 |
| 6 | 1 | 1 | 0 | 0 | 0 | 6 | 2 | 0 | 0 | 0 | 0 |
| 7 | 2 | 0 | 0 | 0 | 0 | 7 | 1 | 2 | 0 | 0 | 0 |
| 8 | 2 | 1 | 1 | 0 | 0 | 8 | 2 | 0 | 1 | 0 | 0 |
| 9 | 1 | 2 | 0 | 1 | 1 | 9 | 1 | 1 | 0 | 1 | 1 |
| 10 | 1 | 0 | 0 | 0 | 0 | 10 | 2 | 2 | 1 | 0 | 0 |
| 2-fold (training data) | | | | | | | | | | | |
| 11 | 1 | 2 | 0 | 0 | 0 | 11 | 0 | 1 | 0 | 0 | 0 |
| 12 | 1 | 1 | 1 | 0 | 1 | 12 | 0 | 1 | 0 | 0 | 0 |
| 13 | 0 | 1 | 0 | 0 | 1 | 13 | 1 | 0 | 0 | 0 | 0 |
| 14 | 0 | 2 | 0 | 0 | 1 | 14 | 1 | 1 | 0 | 0 | 0 |
| 15 | 0 | 1 | 0 | 0 | 0 | 15 | 0 | 0 | 0 | 0 | 0 |
| 16 | 1 | 1 | 0 | 0 | 0 | 16 | 2 | 1 | 0 | 0 | 0 |
| 17 | 1 | 1 | 0 | 0 | 0 | 17 | 1 | 2 | 1 | 1 | 0 |
| 18 | 1 | 1 | 0 | 2 | 0 | 18 | 0 | 0 | 0 | 0 | 0 |
| 19 | 2 | 2 | 0 | 0 | 0 | 19 | 1 | 1 | 0 | 0 | 0 |
| 20 | 2 | 0 | 0 | 0 | 1 | 20 | 0 | 0 | 1 | 0 | 0 |
| 3-fold (training data) | | | | | | | | | | | |
| 21 | 1 | 1 | 0 | 0 | 0 | 21 | 1 | 1 | 0 | 0 | 0 |
| 22 | 1 | 1 | 1 | 0 | 0 | 22 | 2 | 1 | 1 | 1 | 1 |
| 23 | 2 | 0 | 0 | 0 | 0 | 23 | 0 | 0 | 0 | 0 | 0 |
| 24 | 0 | 2 | 0 | 0 | 0 | 24 | 1 | 0 | 0 | 0 | 0 |
| 25 | 0 | 0 | 1 | 1 | 0 | 25 | 1 | 0 | 0 | 0 | 0 |
| 26 | 0 | 0 | 1 | 0 | 0 | 26 | 1 | 0 | 0 | 0 | 0 |
| 27 | 1 | 0 | 0 | 0 | 0 | 27 | 0 | 0 | 0 | 0 | 0 |
| 28 | 1 | 2 | 0 | 0 | 1 | 28 | 0 | 2 | 0 | 0 | 0 |
| 29 | 1 | 1 | 0 | 0 | 0 | 29 | 0 | 0 | 0 | 0 | 0 |
| 30 | 1 | 1 | 1 | 1 | 0 | 30 | 1 | 1 | 0 | 0 | 0 |
| 4-fold (training data) | | | | | | | | | | | |
| 31 | 1 | 0 | 0 | 0 | 0 | 31 | 0 | 0 | 0 | 0 | 0 |
| 32 | 0 | 2 | 0 | 0 | 0 | 32 | 1 | 0 | 0 | 1 | 1 |
| 33 | 0 | 0 | 0 | 0 | 1 | 33 | 1 | 1 | 0 | 0 | 0 |
| 34 | 0 | 2 | 0 | 0 | 0 | 34 | 2 | 1 | 0 | 0 | 0 |
| 35 | 1 | 1 | 0 | 0 | 1 | 35 | 2 | 0 | 0 | 0 | 0 |
| 36 | 1 | 1 | 0 | 0 | 0 | 36 | 0 | 0 | 0 | 0 | 0 |
| 37 | 1 | 2 | 0 | 0 | 0 | 37 | 1 | 1 | 1 | 1 | 1 |
| 38 | 0 | 2 | 0 | 0 | 0 | 38 | 0 | 1 | 0 | 0 | 0 |
| 39 | 0 | 1 | 0 | 0 | 0 | 39 | 0 | 2 | 0 | 1 | 1 |
| 40 | 1 | 2 | 0 | 0 | 0 | 40 | 1 | 0 | 0 | 1 | 2 |
| 5-fold (training data) | | | | | | | | | | | |
| 41 | 2 | 2 | 0 | 0 | 0 | 41 | 1 | 0 | 1 | 0 | 0 |
| 42 | 1 | 1 | 0 | 0 | 0 | 42 | 0 | 1 | 0 | 1 | 1 |
| 43 | 0 | 1 | 0 | 1 | 0 | 43 | 0 | 0 | 0 | 0 | 0 |
| 44 | 1 | 1 | 0 | 0 | 1 | 44 | 2 | 1 | 0 | 0 | 0 |
| 45 | 1 | 1 | 0 | 1 | 0 | 45 | 1 | 1 | 1 | 0 | 0 |
| 46 | 0 | 0 | 0 | 0 | 1 | 46 | 2 | 0 | 0 | 0 | 0 |
| 47 | 1 | 0 | 0 | 0 | 0 | 47 | 1 | 0 | 0 | 1 | 0 |
| 48 | 1 | 0 | 0 | 0 | 0 | 48 | 1 | 1 | 1 | 0 | 0 |
| 49 | 0 | 2 | 0 | 0 | 0 | 49 | 0 | 1 | 0 | 0 | 0 |
| 50 | 1 | 0 | 0 | 0 | 1 | 50 | 1 | 0 | 0 | 1 | 1 |

Generate all feasible multi-factor classes in a feasible combination *p*:

We denote ‘0’ as ‘AA’, ‘1’ as ‘Aa’ and ‘2’ as ‘aa’ in SNP1, and denote ‘0’ as ‘BB’, ‘1’ as ‘Bb’ and ‘2’ as ‘bb’ in SNP3. The all feasible multi-factor classes are represented as below:

|  | AA | | Aa | | aa | |
| --- | --- | --- | --- | --- | --- | --- |
|  | cases | controls | cases | controls | cases | controls |
| BB | 1^th^-1 | 1^th^-0 | 2^th^-1 | 2^th^-0 | 3^th^-1 | 3^th^-0 |
| Bb | 4^th^-1 | 4^th^-0 | 5^th^-1 | 5^th^-0 | 6^th^-1 | 6^th^-0 |
| bb | 7^th^-1 | 7^th^-0 | 8^th^-1 | 8^th^-0 | 9^th^-1 | 9^th^-0 |

Thus, the samples can be classified into the matching classes. For example: A sample (No. 48 in case group) has SNP1=1 (Aa) and SNP3=1 (Bb, indicating the sample is classified into 5^th^-1 class. Therefore, we can get a table using training data.

|  | AA | | Aa | | aa | |
| --- | --- | --- | --- | --- | --- | --- |
|  | cases | controls | cases | controls | cases | controls |
| BB | 14 | 12 | 12 | 19 | 5 | 4 |
| Bb | 2 | 2 | 6 | 2 | 1 | 0 |
| bb | 0 | 0 | 0 | 1 | 0 | 0 |

**Step 1.** Determine high/low risk within the multi-factor class using training data.

Each multi-factor class is determined as a high-risk when the ratio is higher than or equal to 1, otherwise class is determined as a low-risk. The ratio is calculated by Equation 7.

| $\mathop{\theta_{a}}\limits^{\wedge} =\frac{n_{+0}\times n_{a1}}{n_{+1}\times n_{a0}}$ |  |
| --- | --- |

|  | AA | Aa | aa |
| --- | --- | --- | --- |
| BB | 1.167 | 0.632 | 1.25 |
| Bb | 1 | 3 | >1 |
| bb |  | <1 |  |

Thus, each class can be determined high/low risk.

|  | AA | Aa | aa |
| --- | --- | --- | --- |
| BB | High | Low | High |
| Bb | High | High | High |
| bb |  | Low |  |

**Step 2.** Determine high/low risk within the multi-factor class using testing data.

Each multi-factor class is determined as step 3. The ratio is calculated by Equation 8.

| $\mathop{\theta_{a}}\limits=\frac{n_{+0}\times t_{a1}}{n_{+1}\times t_{a0}}$ |  |
| --- | --- |

|  | AA | Aa | aa |
| --- | --- | --- | --- |
| BB | 1 | 1 | 1 |
| Bb |  | 1 | <1 |
| bb |  |  | >1 |

Thus, each class can be determined high/low risk.

|  | AA | Aa | aa |
| --- | --- | --- | --- |
| BB | High | High | High |
| Bb |  | High | Low |
| bb |  |  | High |

**Step 3.** Comparison of the multi-factor class between the risks determined by training data and determined by testing data, and evaluate the *TP*, *FP*, *FN* and *TN*.

Training model

|  | AA | Aa | aa |
| --- | --- | --- | --- |
| BB | High | Low | High |
| Bb | High | High | High |
| bb |  | Low |  |

Testing model

|  | AA | Aa | aa |
| --- | --- | --- | --- |
| BB | High | High | High |
| Bb |  | High | Low |
| bb |  |  | High |

Thus, TP is total number of cases among AA-BB, aa-BB, Aa-Bb and aa-Bb, FP is total number of controls among AA-BB, aa-BB, Aa-Bb and aa-Bb. Thus, FN is total number of cases among Aa-BB, TN is total number of controls among Aa-BB. The AA-Bb, Aa-bb and aa-bb do not be evaluated due to the null in either training model or testing model.

| Risk group by CMDR classifier | Disease group | |
| --- | --- | --- |
|  | Case | Control |
| High | TP | FP |
| Low | FN | TN |

| Risk group by CMDR classifier | Disease group | |
| --- | --- | --- |
|  | Case | Control |
| High | 6 | 7 |
| Low | 3 | 3 |

**Step 4.** Generate values of multi-objective by evaluate a feasible combination *p*.

Objective function 1:

| $\text{CCR}=0.5\times\left(\frac{TP}{TP+FN}+\frac{TN}{FP+TN}\right)$ |  |
| --- | --- |

CCR = 0.55

Objective function 2:

$\begin{matrix} NMI=\frac{H(y)-H(y|x)}{H(y)} \\ =\frac{\begin{matrix} 2\{(TP+FN+TN+FP)\log_{2}(TP+FN+TN+FP)+TP\log_{2}TP+FN\log_{2}FN+ \\ TN\log_{2}TN+FP\log_{2}FP-(TP+FP)\log_{2}(TP+FP)-(TP+FN)\log_{2}(TP+FN)- \\ (TN+FP)\log_{2}(TN+FP)-(TN+FN)\log_{2}(TN+FN)\} \end{matrix}}{\begin{matrix} 2\{(TP+FN+TN+FP)\log_{2}(TP+FN+TN+FP)- \\ \text{(}TP+FN\text{)log}_{2}\text{(}TP+FN\text{)}-\text{(}TN+FP\text{)log}_{2}\text{(}TN+FP\text{)\}} \end{matrix}} \end{matrix}$

*NMI* = 9.261E-4

**Step 5.** Repeat steps 1 to 4 until all CV fold done.

Thus, we can get 5 CCR values of SNP1-SNP3.

|  | CCR | *NMI* |
| --- | --- | --- |
| 1-fold CV | 0.550 | 9.261E-4 |
| 2-fold CV | 0.500 | 0.016 |
| 3-fold CV | 0.450 | 0.007 |
| 4-fold CV | 0.500 | 0.007 |
| 5-fold CV | 0.500 | 0.030 |

**Step 6.** Compute averages of CCR values and of NMI values in all CV fold within a feasible combination.

Thus, we can get aCCR and aNMI of SNP1-SNP3.

aCCR = (0.55 + 0.5 + 0.45 + 0.5 + 0.5) / 5 = 0.5

aNMI = (9.261E-4 + 0.016 + 0.007 + 0.007 + 0.03) / 5 = 0.061

Therefore,

**Equation:**

| Target vector_1_ fitness value:  *X*_1,0_ = (1, 3), aCCR = 0.500, aNMI = 0.061  *U*_1,1_ = (1, 3), aCCR = 0.500, aNMI = 0.061 | Target vector_2_ fitness value:  *X* _2,0_ = (2, 3), aCCR = 0.524, aNMI = 0.038  *U*_2,1_ = (5, 3), aCCR = 0.500, aNMI = -2.15 |
| --- | --- |
| Target vector_3_ fitness value:  *X* _3,0_ = (1, 5), aCCR = 0.510, aNMI = 0.036  *U*_3,1_ = (2, 5), aCCR = 0.458, aNMI = 0.107 | Target vector_4_ fitness value:  *X* _4,0_ = (3, 4), aCCR = 0.580, aNMI = 0.048  *U*_4,1_ = (1, 4), aCCR = 0.560, aNMI = 0.044 |
| Target vector_5_ fitness value:  *X* _5,0_ = (2, 4), aCCR = 0.494, aNMI = 0.039  *U*_5,1_ = (2, 5), aCCR = 0.458, aNMI = 0.107 | Target vector_6_ fitness value:  *X* _6,0_ = (3, 5), aCCR =0.500, aNMI = -2.15  *U*_6,1_ = (1, 5), aCCR = 0.510, aNMI = 0.036 |

| In target vector_1_:  ∵*X*_1_ = *U*_1_  ∴*X*_1_ is not replaced by *U*_1_  => *X*_1,1_ = (1, 3) | In target vector_2_:  ∵*X*_2_ dominates *U*_2_  ∴*X*_2_ is not replaced by *U*_2_  => *X*_2,1_ = (2, 3) |
| --- | --- |
| In target vector_3_:  ∵*X*_3_ is not dominated by *U*_3_  ∴*X*_3_ is not replaced by *U*_3_  => *X*_3,1_ = (1, 5) | In target vector_4_:  ∵*X*_4_ dominates *U*_4_  ∴*X*_4_ is not replaced by *U*_4_  => *X*_4,1_ = (3, 4) |
| In target vector_5_:  ∵*X*_5_ is not dominated by *U*_5_  ∴*X*_5_ is not replaced by *U*_5_  => *X*_5,1_ = (2, 4) | In target vector_6_:  ∵*X*_6_ is dominated by *U*_6_  ∴*X*_6_ is replaced by *U*_6_  => *X*_6,1_ = (1, 5) |

**4**. Evaluate whether stopping criteria are met.

The stopping criteria is not met. Therefore, the next generation is implemented.

**2. Pareto operation**

Pareto set filter operator has two steps.

(1) Comparison of a *X* ∈ population and *s_j_* for all indexes *j* ∈ {1, 2, …, *i*} in *S.* If that *X* is not dominated by any *s_j_*, then *X* is added into *S*; otherwise, *X* is not added. All target vectors are added into storage because the storage *S* = {∅}.

Therefore,

*S*= {*s*_1_={1, 3}, *s*_2_={2, 3}, *s*_3_={1,5}, *s*_4_={3, 4}, *s*_5_={2,4}, *s*_6_={1,5}}

(2) Comparison of a s*_j_* for index *j* ∈ {1, 2, …, *i*} and s*_k_* for other indexes *k*, *k* ∈ {1, 2, …, *i | k j* } in *S*. If that s*_j_* is dominated by any s*_k_*, then s*_j_* is discarded.

Therefore,

*S*={ *s*_1_={1, 3}, *s*_2_={3, 4}}

**3. Mutation operation, recombination operation, boundary constraints, selection operation, and evaluating whether stopping criteria are met.**

3.1. Mutation operation

Mutation operation of target vector_1_:

Suppose *randb* = {0.2, 0.7, 0.6}, thus *r*_1_ is randomly selected from Pareto storage and *r*_2_ and *r*_3_ are randomly selected from population. Suppose *r*_1_ = 1, *r*_2_ = 2 and *r*_3_ = 6 and *F* set to 0.5.

*V_i,G+_*_1_ = *X_r_*_1_*_,G_* + *F‧*(*X_r_*_2_*_,G_* – *X_r_*_3_*_,G_*)

*=> V*_1,1+1_ = *s*_1_ + 0.5*‧*(*X*_2,1_ – *X*_6,1_)

=> *V*_1,2_ = (1, 3) + 0.5*‧*{(2, 3) – (1, 5)}

=> *V*_1,2_ = ((1 + 0.5*‧*(2 – 1)), (3 + 0.5*‧*(3 – 5)))

=> *V*_1,2_ = (1.5, 2)

Mutation operation of target vector_2_:

Suppose *randb* = {0.6, 0.2, 0.4}, thus *r*_2_ and *r*_3_ are randomly selected from Pareto storage and *r*_1_ is randomly selected from population. Suppose *r*_1_ = 4, *r*_2_ = 2 and *r*_3_ = 1 and *F* set to 0.5.

*V_i,G+_*_1_ = *X_r_*_1_*_,G_* + *F‧*(*X_r_*_2_*_,G_* – *X_r_*_3_*_,G_*)

*=> V*_2, 1+1_ = *X*_4,1_ + 0.5*‧*(*s_2_* – *s_1_*)

=> *V*_2, 2_ = (3, 4) + 0.5*‧*{(3, 4) – (1, 3)}

=> *V*_2, 2_ = ((3 + 0.5*‧*(3 – 1)), (4 + 0.5*‧*(4 – 3)))

=> *V*_2, 2_ = (4, 4.5)

Mutation operation of target vector_3_:

Suppose *randb* = {0.1, 0.6, 0.4}, thus *r*_1_ and *r*_3_ are randomly selected from Pareto storage and *r*_2_ is randomly selected from population. Suppose *r*_1_ = 1, *r*_2_ = 5 and *r*_3_ = 2 and *F* set to 0.5.

*V_i,G_*_+1_ = *X_r_*_1_,*_G_* + *F‧*(*X_r_*_2_*_,G_* – *X_r_*_5_*_,G_*)

*=> V*_3,1+1_ = *s_1_* + 0.5*‧*(*X*_5,1_ – *s_2_*)

=> *V*_3,2_ = (1, 3) + 0.5*‧*{(2, 4) – (3, 4)}

=> *V*_3,2_ = ((1 + 0.5*‧*(2 – 3)), (3 + 0.5*‧*(4 – 4)))

=> *V*_3,2_ = (0.5, 3)

Mutation operation of target vector_4_:

Suppose *randb* = {0.8, 0.6, 0.7}, thus *r*_1_, *r*_2_ and *r*_3_ are randomly selected from population. Suppose *r*_1_ = 1, *r*_2_ = 2 and *r*_3_ = 5 and *F* set to 0.5.

*V_i,G_*_+1_ = *X_r_*_1_*_,G_* + *F‧*(*X_r_*_2_*_,G_* – *X_r_*_3_*_,G_*)

*=> V*_4,1+1_ = *X*_1,1_ + 0.5*‧*(*X*_2,1_ – *X*_5,1_)

=> *V*_4,2_ = (1, 3) + 0.5*‧*{(2, 3) – (2, 4)}

=> *V*_4,2_ = ((1 + 0.5*‧*(2 – 2)), (3 + 0.5*‧*(3 – 4)))

=> *V*_4,2_ = (1, 2.5)

Mutation operation of target vector_5_:

Suppose *randb* = {0.1, 0.3, 0.3} but the storage size is two; therefore, *r*_1_ and *r*_2_ are randomly selected from Pareto storage and *r*_1_ is randomly selected from population. Suppose *r*_1_ = 2, *r*_2_ = 1 and *r*_3_ = 3 and *F* set to 0.5.

*V_i,G_*_+1_ = *X_r_*_1_*_,G_* + *F‧*(*X_r_*_2_*_,G_* – *X_r_*_3_*_,G_*)

*=> V*_5,1+1_ = *s_2_* + 0.5*‧*(*s_1_* – *X*_3,1_)

=> *V*_5,2_ = (3, 4) + 0.5*‧*{(1, 3) – (1, 5)}

=> *V*_5,2_ = ((3 + 0.5*‧*(1 – 3)), (4 + 0.5*‧*(3 – 5)))

=> *V*_5,2_ = (2, 3)

Mutation operation of target vector_6_:

Suppose *randb* = {0.1, 0.6, 0.7}, thus *r*_1_ is randomly selected from Pareto storage and *r*_2_ and *r*_3_ are randomly selected from population. Suppose *r*_1_ = 1, *r*_2_ = 4 and *r*_3_ = 1 and *F* set to 0.5.

*V_i,G_*_+1_ = *X_r_*_1_*_,G_* + *F‧*(*X_r_*_2_*_,G_* – *X_r_*_3_*_,G_*)

*=> V*_6,1+1_ = *s_1_* + 0.5*‧*(*X*_4,1_ – *X*_1,1_)

=> *V*_6,2_ = (1, 3) + 0.5*‧*{(3, 4) – (1, 3)}

=> *V*_6,2_ = ((1 + 0.5*‧*(3 – 1)), (3 + 0.5*‧*(4 – 3)))

=> *V*_6,2_ = (2, 3.5)

**3.2.** Recombination operation

Recombination of target vector_1_:

Suppose a randomly generated *randb* = {0.6, 0.2}

The 2^th^ dimension is smaller than *CR* (0.5).

Therefore,

*U*_1_,*_G_*_+1_ = (*X*_1,1,1_, *V*_2,1,2_)

*=> U*_1,2_ = (1, 2)

Recombination of target vector_2_:

Suppose a randomly generated *randb* = {0.2, 0.6}

The 1^th^ dimension is smaller than *CR* (0.5).

Therefore,

*U*_2_,*_G_*_+1_ = (*V*_1,2,2_, *X*_2,2,1_)

*=> U*_2,2_ = (4, 3)

Recombination of target vector_3_:

Suppose a randomly generated *randb* = {0.4, 0.8}

The 1^th^ dimension is smaller than *CR* (0.5).

Therefore,

*U*_3_,*_G_*_+1_ = (*V*_1,3,2_, *X*_2,3,1_)

*=> U*_3,2_ = (0.5, 5)

Recombination of target vector_4_:

Suppose a randomly generated *randb* = {0.1, 0.9}

The 1^th^ dimension is smaller than *CR* (0.5).

Therefore,

*U*_4_,*_G_*_+1_ = (*V*_1,4,2_, *X*_2,4,1_)

*=> U*_4,2_ = (1, 4)

Recombination of target vector_5_:

Suppose a randomly generated *randb* = {0.6, 0.7}

No dimension is smaller than *CR* (0.5).

Therefore,

*U*_5_,*_G_*_+1_ = (*X*_1,5,1_, *X*_2,5,1_)

*=> U*_5,2_ = (2, 4)

Recombination of target vector_6_:

Suppose a randomly generated *randb* = {0.3, 0.6}

The 1^th^ dimensions are smaller than *CR* (0.5).

Therefore,

*U*_6_,*_G_*_+1_ = (*V*_1,6,2_, *X*_2,6,1_)

*=> U*_6,2_ = (2, 5)

**3.3.** Boundary constraints

*U*_1,2_ = (1, 2)

*U*_2,2_ = (4, 3)

*U*_3,2_ = (0.5, 5) => (1, 5)

*U*_4,2_ = (1, 4)

*U*_5,2_ = (2, 4)

*U*_6,2_ = (2, 5)

**3.4.** Selection operation

| Target vector_1_ fitness value:  *X*_1,1_ = (1, 3), aCCR = 0.531, aNMI = 0.061  *U*_1,2_ = (1, 2), aCCR = 0.644, aNMI = 0.131 | Target vector_2_ fitness value:  *X* _2,1_ = (2, 3), aCCR = 0.524, aNMI = 0.038  *U*_2,2_ = (4, 3), aCCR = 0.580, aNMI = 0.048 |
| --- | --- |
| Target vector_3_ fitness value:  *X* _3,1_ = (1, 5), aCCR = 0.510, aNMI = 0.036  *U*_3,2_ = (1, 5), aCCR = 0.510, aNMI = 0.036 | Target vector_4_ fitness value:  *X* _4,1_ = (3, 4), aCCR = 0.580, aNMI = 0.048  *U*_4,2_ = (1, 4), aCCR = 0.560, aNMI = 0.044 |
| Target vector_5_ fitness value:  *X* _5,1_ = (2, 4), aCCR = 0.494, aNMI = 0.039  *U*_5,2_ = (2, 4), aCCR = 0.494, aNMI = 0.039 | Target vector_6_ fitness value:  *X* _6,1_ = (3, 5), aCCR =0.500, aNMI = -2.15  *U*_6,2_ = (2, 5), aCCR = 0.480, aNMI = 0.107 |

| In target vector_1_:  ∵*X*_1_ is dominated by *U*_1_  ∴*X*_1_ is replaced by *U*_1_  => *X*_1,2_ = (1, 2) | In target vector_2_:  ∵*X*_2_ is dominated by *U*_2_  ∴*X*_2_ is replaced by *U*_2_  => *X*_2,1_ = (4, 3) |
| --- | --- |
| In target vector_3_:  ∵*X*_3_ = *U*_3_  ∴*X*_3_ is not replaced by *U*_3_  => *X*_3,1_ = (1, 5) | In target vector_4_:  ∵*X*_4_ is not dominated by *U*_4_  ∴*X*_4_ is not replaced by *U*_4_  => *X*_4,1_ = (3, 4) |
| In target vector_5_:  ∵*X*_5_ = *U*_5_  ∴*X*_5_ is not replaced by *U*_5_  => *X*_5,1_ = (2, 4) | In target vector_6_:  ∵*X*_6_ is not dominated by *U*_6_  ∴*X*_6_ is not replaced by *U*_6_  => *X*_6,1_ = (3, 5) |

**4**. Evaluate whether stopping criteria are met.

The stopping criteria is met. Therefore, final Pareto operation is implemented.

**2. Pareto operation**

(1) Comparison of a *X* ∈ population and *s_j_* for all indexes *j* ∈ {1, …, *i*} in *S.*

Therefore,

*S*= {*s*_1_={1, 3}, *s*_2_={3, 4}, ***s*_3_={1, 2}**}

(2) Comparison of a s*_j_* for index *j* ∈ {1, …, *i*} and s*_k_* for other indexes *k*, *k* ∈ {1, …, *i | k j* } in *S*

Therefore,

*S*={ *s*_1_={1, 2}}

The final result is the vector in Pareto storage. In this example, a best model is SNPs (*SNP*_1_, *SNP*_2_) in which the aCCR = 0.644 and the aNMI = 0.131.
